# Recent advances in green carbon dots (2015–2022): synthesis, metal ion sensing, and biological applications

**DOI:** 10.3762/bjnano.13.93

**Published:** 2022-10-05

**Authors:** Aisha Kanwal, Naheed Bibi, Sajjad Hyder, Arif Muhammad, Hao Ren, Jiangtao Liu, Zhongli Lei

**Affiliations:** 1 Key Laboratory of Applied Surface and Colloid Chemistry, School of Chemistry & Chemical Engineering, Shaanxi Normal University, Xi’an, 710119, China; 2 Department of Chemistry, Shaheed Benazir Bhutto Women University, Charsadda Road, Larama, Peshawar, Pakistan; 3 Department of Botany, Government College Women University, Sialkot, Pakistan; 4 College of Pharmacy, Shaanxi University of Chinese Medicine, Xianyang, 712046, China

**Keywords:** bioimaging, carbon dots, carbon quantum dots, green synthesis, plant growth promotion, sensing

## Abstract

Carbon dots (CDs) show extensive potential in various fields such as sensing, bioimaging, catalysis, medicine, optoelectronics, and drug delivery due to their unique properties, that is, low cytotoxicity, cytocompatibility, water-solubility, multicolor wavelength tuned emission, photo-stability, easy modification, strong chemical inertness, etc. This review article especially focuses on the recent advancement (2015–2022) in the green synthesis of CDs, their application in metal ions sensing and microbial bioimaging, detection, and viability studies as well as their applications in pathogenic control and plant growth promotion.

## Introduction

Carbon dots (CDs) are a carbon-based nanomaterial with a few nanometers feature sizes. CDs consist of a carbon core, the surface of which is functionalized with various groups. Xu et al. accidentally discovered fluorescent carbon nanoparticles during electrophoretic purification of single-walled carbon nanotubes [1]. Sun et al. synthesized fluorescent carbon particles smaller than 10 nm, which were named “carbon dots” for the first time in 2006 [2]. Due to its significant fluorescent properties, this class of carbon nanomaterials has proved to be useful for applications in a variety of disciplines, including chemical or biological sensing, bioimaging, drug delivery, photodynamic therapy, electrocatalysis, and photocatalysis, with advantages over commonly used semiconductor dots or conventional fluorescent probes such as organic dyes. Moreover, CDs have unparalleled extraordinary properties, including cell compatibility, chemical inertness, emission at tunable wavelengths, low cost, high quantum yield (QY), water dispersibility, small size, tunability, high biocompatibility, strong photostability (resistance to photobleaching), and efficient photoluminescence. CDs have a broad spectrum of applications in the analytical, medical, biotechnology, biology, and theranostics domains [3–4]. The typical photoluminescence yield of CDs is less than 10%. Surface-passivating chemicals are used to improve the photoluminescence. Surface passivation with different functional groups generates surface defects, which produces fluorescence and also generates new active sites for modification for specific applications. CDs can be chemically modified by many heteroatoms, including N, P, and S, and many other chemicals that increase their functional properties [5–6]. These exceptional optical and physicochemical properties make them ideal for transdisciplinary research. Fluorescent CDs can be manufactured using inexpensive, naturally rich carbon sources in an environmentally friendly manner. By adjusting the surface chemistry of CDs, the solubility and QY can be improved. The size of CDs and chemical functionality present on their surface can be discreetly tuned to change the electronic structure for their luminous features.

Various molecular precursors have been used earlier for the production of CDs, including ethylene glycol [6], phytic acid [7], phenylenediamine [8], ammonium citrate [9], citric acid [10], ethylene diamine tetra acetic acid [11], carbon nanotubes [12], and graphite [13]. Additionally, graphite, nanodiamonds, and activated carbon can be applied as precursor for the fabrication of CDs [14]. Meanwhile, a variety of green carbon precursors have been utilized for generating CDs, including fruits, their juices and peels [15–17], animal and animal-derived materials, such as milk and hair [18–20], and vegetables [21], flowers [22], and leaves [23]. The use of green, sustainable or waste materials for the production of CDs is congruous with the objectives of a sustainable development strategy. Owing to the recycling and re-use of organic waste products, environmental friendliness, and low-cost, green synthesis of CDs is preferred over other conventional methods.

There are, in general, two synthetic pathways for the formation of CDs, that is, “top-down” and “bottom-up” methods. In the top-down method, large carbon structures (such as carbon nanotubes or graphite) are decomposed into CDs. The top-down methods include arc discharge, laser abrasion [24], chemical and electrochemical oxidation, and ultrasonic synthesis. In the bottom-up methods, CDs are formed from molecular precursors by various techniques such as hydrothermal treatment [25–29], microwave synthesis [30], and pyrolysis [31].

**Figure 1:**
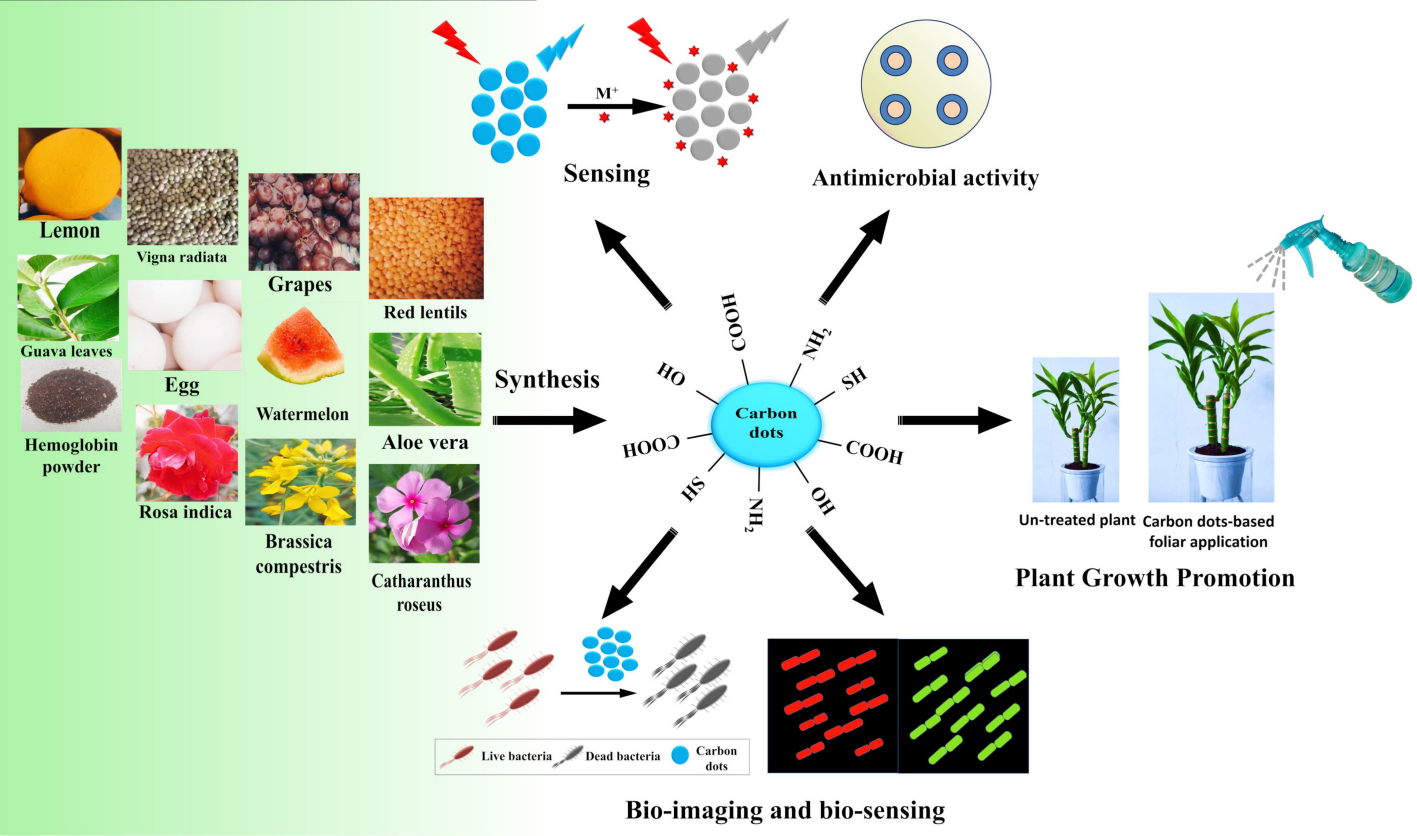
Illustration of the environmentally friendly sources employed for the synthesis of CDs and their applications from 2015–2022.

A tremendous amount of work has been done regarding the synthesis and different techniques for improving synthesis, characterization, yield, and applications of CDs There are several outstanding review articles on different applications, such as photochemical and electrochemical applications [32], photocatalysis [33], optoelectronics [34], wastewater treatment [35], food safety applications [36], tumor marker detection [37], bioanalytical studies [38], biomedical [39–40] and biotechnological applications [3], biosensing and bioimaging [31–32], and fluorescence [41] and photoluminescence processes [42]. Many reviews about CDs obtained from natural resources have been published. Sharma et al. reported an excellent review article on the green synthesis of CDs in 2017 [43]. Recently, Tejwan et al. [44] and Lin et al. [45] also reported review articles about synthesis and applications of CDs obtained from green precursors. Meng et al. reviewed CDs made from biomass and their applications [46]. A study on recent advancements in the synthesis of CDs from natural resources and their applications in biomedicine and multisensing platforms was also published by Bag and co-workers [47]. The review presented here is focused on the latest progress in the field since 2015. It describes the synthesis of CDs, the effects of surface states on optical properties, the characterization of CDs, metal ion sensing, and biological and agricultural applications of CDs, that is, microbial bioimaging, detection, and viability studies, pathogen control, and plant growth promotion (Figure 1).

## Review

### Green synthesis of carbon dots

Green synthesis of CDs mainly utilizes biomass. Biomass synthesis makes use of natural raw materials (organisms, waste material, protein products, or natural polymers), instead of reaction precursors usually used in the traditional methods, and also requires external energy supply. Using diverse raw materials, CDs with different structures and properties can be obtained. Usually, a temperature of 100–200 °C is required, which is much lower than that required in the traditional methods, and the synthesis is carried out in aqueous media. Generally, hydrothermal or solvothermal treatments, ultrasonication, microwave irradiations, and microwave-assisted hydrothermal/pyrolysis are used in the green synthesis of CDs [41]. Hydrothermal methods convert the raw material into carbonized matter. Although relatively simple, the procedure takes several hours. Microwave irradiation, in contrast, provides homogenous and effective heating and speed up the reaction to merely a few minutes. Hence, this approach is considered the fastest and simplest amongst the synthesis methodologies and has become widely used.

The fluorescence emissions of CDs are usually blue and green (i.e., in the low-wavelength region). Since biomass is abundant in carbon and oxygen, the resulting CDs have carbonyl groups on their surface. Excited electrons resulting from n–π* transitions emit blue fluorescence on radiative recombination. In general, blue fluorescence arise as a result of π–π* transition of the carbon core, whereas, green fluorescence may correspond to n–π* transitions of the edge states [48–49].

The “top-down” approach involves breaking down bulky carbonaceous materials, such as carbon fibers, carbohydrates, proteins, and carbon soot, through chemical or physical methods. The carbon containing material is oxidized and broken down into CDs using oxidants such as sulfuric acid and nitric acid. As green methods are limited regarding the raw materials, the “top-down” method is not very common in green approaches [3,50].

The “bottom-up” method consist of carbonization of smaller organic molecules. This method basically involves four phases, that is, condensation of the molecules followed by polymerization, carbonization, and passivation. Small molecules are condensed into intermediate chains and then polymerized into clusters of carbonaceous material. Carbonization of this material at elevated temperatures leads to the formation of carbon cores. The residual groups on the surface act as surface-passivating agents and can be manipulated to ameliorate surface luminescence properties [38]. Biomass is rich in small organic compounds suitable for carbonization at elevated temperature and, hence, “bottom-up” approaches are extensively used for the green synthesis of CDs.

In this review, CDs have been classified into various categories based on their precursor materials, including plant sources, animal extracts, and food materials. We focus on the CDs obtained using various green precursors and their modification with or without different surface passivizing agents.

### Plant sources

Synthesis of CDs from plant-based sources has the potential to be scaled up and comes with a number of benefits, including reduced chemical exposure, cost-effectiveness, renewability of sources, waste reduction, and ample source availability. It is thus environmentally friendly and advantageous [51–52]. Plant parts such as roots, stem, leaves, fruits, flowers, and seeds have been used for the production of CDs. Several low-value plant materials can also be converted into functional materials with excellent biocompatibility by manufacturing CDs from these plant components. Plant-based precursors that contain heteroatoms (nitrogen and sulfur) are preferred over carbon sources that demand supplementary heteroatoms for the synthesis of CDs [53].

**Without surface-passivating agent:** Plants are rich in biomolecules such as carbohydrates and proteins, which makes them a better option based on the fact that surface functionality of CDs can be achieved without adding extra substances for doping, modification and surface passivation [54]. A summary of CDs synthesized from carbon-rich plant extract without surface-passivating agents and their quantum yield is provided in Table 1. Liu et al. used grass as a natural carbon source for the first time to prepare CDs [55]. Bhamore and co-workers reported fluorescent CDs without any surface-passivating agent by using a green precursor *Pyrus pyrifolia* fruit through a simple hydrothermal method at 180 °C for a period of 6 h [56]. *Gynostemma* has been used to prepare fluorescent CDs through a simple calcination method without any harmful substances or any surface modification [57].

**Table 1:** Summary of CDs synthesized from carbon-rich plant extracts without surface-passivating agents with their quantum yield.

S. No	Precursor of CDs	Synthetic Method	Size (nm)	QY (%)	Ref

1	biomass waste	hydrothermal method at 200 °C for 8 h	2.60	8.13	[58]
2	*Manilkara zapota*	ultrasonication at 100 °C for 60 min	1.9 ± 0.3, 2.9 ± 0.7 and 4.5 ± 1.25	5.7, 7.9, 5.2	[48]
3	*Abelmoschus manihot*	hydrothermal at 220 °C for 4 h	9	30.8	[22]
4	*Prosopis juliflora*	hydrothermal method at 200 °C for 1 h	5.8	5	[23]
5	*Pyrus pyrifolia*	hydrothermal method at 180 °C for 6 h	2.0 ± 1.0	10.8	[56]
6	*Gynostemma*	calcination method at 400 °C for 4 h	2.5	—	[57]
7	*Borassus flabellifer*	thermal pyrolysis at 300 °C for 2 h	3 to 8 ±1	13.97	[59]
8	lemon juice/orange juice	hydrothermal method at 280 °C for 12 h	3–5	14.8 to 24.8	[60]
9	lychee waste	solvothermal at 180 °C for 5 h	3.13	23.5	[61]
10	spices	hydrothermal method at 200 °C for 12 h	3.5 ± 0.1	43.6	[62]
11	pseudo-stem of banana	hydrothermal treatment at 180 °C for 2 h	1–3	48	[63]
12	Cotton linter	microwave-assisted hydrothermal process in 5 min	10.14	—	[64]
13	*Brassica compestris*	hydrothermal reaction at 240 °C for 20 h	1.9	21	[65]
14	*Setcreasea purpurea* boom	pyrolysis at 300 °C for 2 h	3.9	18	[66]
15	*Aloe Vera*	pyrolysis method (160–250 °C) and time (10–30 min)	6–8	12.3	[67]
16	date palm fronds	one step carbonization method 300 °C	35	33.7	[68]
17	whey	pyrolysis at 220 °C	4	≈11.4	[69]
18	*Nigella sativa* seeds	hydrothermal method at 120 °C for 12 h	4	—	[18]
19	palmyra palm leaf	hydrothermal method at 180 °C for 12 h	5–10	—	[70]
20	guava leaf	hydrothermal method at 160 °C for 1 h	4–7	34	[71]
21	*Murraya koenigii* (curry leaves)	hydrothermal method at 180 °C for 4 h	2–8	5.4	[72]
22	roasted gram	pyrolysis at 200 °C (C-1) and 450 °C (C-2) for 8 h	5.5 and 2	—	[73]
23	rice fried *Codonopsis pilosula* (CP)	Ultrasonic-assisted solvent extraction at room temp for 4 h	9.60 and 11.54	12.8	[74]
24	rose pigments	hydrothermal method at 200 °C for 2 h	32	48	[75]
25	milk	hydrothermal treatment at 180 °C for 4 h	10	10	[76]
26	crop biomasses	hydrothermal process at 140 °C	2–5	—	[77]
27	corn stalk shell	hydrothermal process in NCW at 270 °C, pressure of 5 MPa for 10 min	1.2 to 3.2	16	[78]
28	banana peel	hydrothermal process at 200 °C for 24 h	5	20	[79]
29	*Calotropis gigantea*	microwave at 900 W	2.7 to 10.4	4.24	[80]
30	*Morus nigra*	hydrothermal process at 200 °C for 24 h	4.5	24	[81]
31	ginkgo kernels	hydrothermal process at 220 °C for 12 h	2.7	37.8	[82]

**Figure 2:**
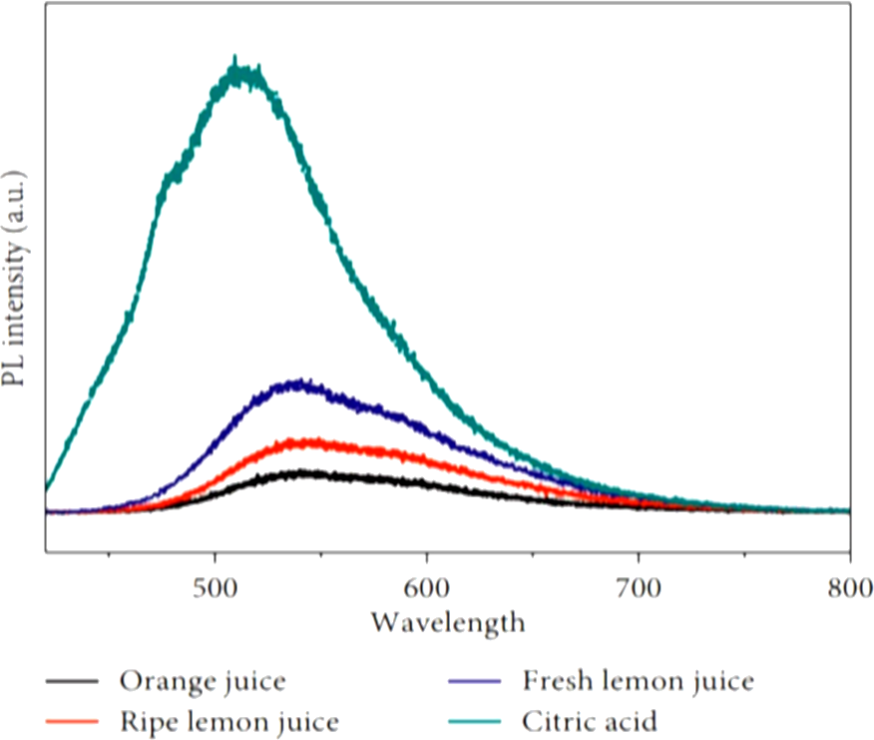
Luminescence of CDs synthesized from citric acid and various green sources. Figure 2 was reproduced from [60] (© 2020 B. T. Hoan et al., published by Hindawi, distributed under the terms of the Creative Commons Attribution 4.0 International License, https://creativecommons.org/licenses/by/4.0).

A new method to recycle biomass was used by Wang et al. They synthesized CDs via simple hydrothermal methods by using ordinary biomass waste as carbon source, namely orange peel, ginkgo leaves, paulownia leaves, and magnolia flowers [58]. Water-soluble CDs using *Manilkara zapota* fruit as a natural source of carbon were reported where sulfuric acid and phosphoric acid were used to regulate the emission of CDs, producing CDs with blue, green, and yellow emission. For the blue, green, and yellow emitting CDs, the obtained QY values were 5.7%, 7.9%, and 5.2%, respectively. The reported average size was 1.9 ± 0.3 nm for the blue emitting CDs, 2.9 ± 0.7 nm for the green emitting CDs, and 4.5 ± 1.25 nm for the yellow emitting CDs [48]. A new type of fluorescent CDs from the flowers of *Borassus flabellifer* (male tree) through a green thermal pyrolysis method without adding any chemicals was synthesized [59]. A high QY of up to 13.97% was obtained at an optimized temperature of 300 °C. Hoan et al. used lemon juice to produce highly luminous CDs via a simple, low-cost hydrothermal route. This novel study explained how hydrothermal time and source type affects the luminescence of CDs [60]. To study the effect of citric acid on the precursors, different precursors (such as ripe lemon juice, fresh lemon juice, and orange juice) were tested. It was found that the photoluminescence (PL) intensity of citric acid was higher than that of lemon juice, which, in turn, was higher than that of orange juice. This outcome is the result of lemon juice having a greater citric acid concentration than orange juice. Compared to ripe lemon juice, fresh lemon juice had a higher PL intensity (Figure 2). Because in the case of the ripe lemon juice, a significant reduction in the quality of the components occurred, which might have led to reduced PL emission. It was also found that longer hydrothermal times could lead to more carbonization. Apart from this, as the temperature increased from 150 to 280 °C, QY was found to increase from 14.86 to 24.89%. These outcomes might be explained by the efficient carbonization of naturally occurring acidic components as the hydrothermal temperature of the reaction rises [60]. The resulting CDs showed a direct relationship between PL intensity and heating temperature and time. These CDs had good green emission. The maximum emission wavelength clearly depends on the excitation wavelength at low temperatures. However, at high hydrothermal temperature, the luminous peak was almost completely independent of the excitation wavelength. The irregular size of the particles at low temperatures is the first reason. CDs of different sizes have different bandgaps. Particles of the same size will prioritize the emission when light of a particular wavelength is projected into CDs. Different sized particles will emit radiation when different wavelengths are projected. However, at high hydrothermal temperatures, uniformity of the particle size of the CD prevents the wavelength from being influenced by the excitation wavelength. The surface states are another factor. It is well known that surface functional groups, such as carbonyl and carboxylic groups, can produce their own energy levels. Hence, there will be a variety of routes for electrons to move from the excited state to the ground state of photon emission. Numerous functional groups can be found on the surface of CDs at low temperatures, but at high temperatures, the COOH, C–H, and C–O–C groups are eliminated and the C=O group takes their place. This explains why CDs generated at high hydrothermal temperatures exhibit excitation-independent luminescence.

Sahoo et al. used the bark of lychee, without any other chemical, to synthesize CDs with a QY of 23.5% and the reported CDs were employed as a sensing probe for Fe^3+^ ions [61]. Devi et al. utilized *Carica papaya* waste as a natural source for a simple pyrolysis route and obtained a good QY of 23.7%. The reported CDs have a chromium sensitivity of 0.708 ppb in water [83]. Recently, a further increase in QY was obtained up to 30.8% by using *Abelmoschus manihot* flowers. It was found that 2,4,6-trinitrophenol (TNP) can quickly and sensitively quench the fluorescence intensity of these CDs. The linear response of TNP ranges from 25 nM to 40 μM and the LOD was 5 nM [22]. A good increase in QY was also obtained by Vasimalai et al. using various spices, such as cinnamon, red chili, turmeric, and black pepper, for a simple hydrothermal method. They found that black pepper yields CDs with the highest QY up to 43.6% [62].

Vandarkuzhali et al. also obtained a very high quantum yield of 48% using pseudo-stem of banana [63]. The source used here consists of cellulose, hemicellulose, and lignin, and hydrothermal treatment was carried out. Li et al. reported a very new approach to synthesize two types of CDs with different selectivities from Hongcaitai (*Brassica compestris* L. var. *Purpurea Bailey*) [65]. The two types were obtained based on the difference in solubility in ethanol. The ones that were soluble in ethanol were called “CDs-A” and the insoluble ones were called “CDs-B”. Different characterizations were carried out that revealed that the surfaces of these two CDs have different functional groups resulting in different selectivity. CDs-A and CDs-B were utilized to detect ClO^−^ and Hg^2+^ in tap water and river water, respectively.

Using cotton linters as a green source, water-dispersed fluorescent CDs were reported, obtained through a microwave-assisted hydrothermal method. This method provides CDs for fast, low-cost, and convenient cancer imaging applications. The particle size of the CDs was 10.14 nm as calculated by TEM [64].

Zhai et al. compared different synthetic methods to find the most suitable route to obtain fluorescent CDs from the green precursor *Setcreasea purpurea* boom [66]. The different methods used were hydrothermal method, and pyrolysis in a vacuum tube furnace or a muffle furnace. Due to incomplete carbonization and oxidation in the hydrothermal method and pyrolysis in the muffle furnace, respectively, pyrolysis in a vacuum tube furnace was selected as a suitable synthesis method to prevent over-oxidation of CDs. It was found that blue fluorescent CDs with high QY were obtained at 300 °C with 2 h reaction time. Increased temperatures, however, may cause a complete carbonization process as well as small CDs, which can lead to higher QY.

A simple and easy preparatory method that does not require complex post-chemical and ultrafiltration treatments, dialysis, and centrifugation was reported using table sugar. The resulting CDs aggregate and scatter light and help to visually detect Pb^2+^ ions by the turbidimetry method. It was found that there is a direct linear relationship between the concentration of Pb^2+^ ions and the turbidity [84].

*Aloe vera* extract, without any other chemical reagent, was used to prepare CDs using a one-step pyrolysis method by Devi et al. Different characterization results demonstrated that the CDs displayed excitation-independent behavior and had surface groups such as carboxyl and hydroxy groups. *Aloe vera* has intrinsic antimicrobial properties, so the bactericidal activity of these CDs was investigated by the agar well diffusion method, and the sensing ability towards Fe^3+^ was also reported [67]. Kavitha et al. used date palm fronds with no other chemical reagent. The lignin content of the leaves and shafts of date palm leaves is 25 g/100 g, which helped to obtain mesoporous CDs of high quality having high storage and photostability [68]. Leaves of *Prosopis juliflora* as a natural and inexpensive precursor yielded CDs that acted as a dual fluorescence sensor for both Hg^2+^ ions and chemet drugs [23].

An acid oxidation approach was applied to synthesize nanosized fluorescent CDs of various colors by using *Ananas comosus* without any passivating agent by Gupta et al. The synthesized CDs showed three emission peaks at 438, 516, and 543 nm when excited at 325, 417, and 425 nm, respectively. Their selectivity for various metal ions indicated that only Fe^3+^ ions significantly quenched the emission intensity at 438 nm and the CDs were used as fluorescence sensors to detect Fe^3+^ ions [85]. Devi et al. adopted an eco-friendly, simple, and green synthesis strategy to prepare CDs by using whey (a major dairy waste). NMR analysis showed that during pyrolysis, polymerization of lactose, which is the main component of the green source used, takes place [69].

Sharma et al. reported *Nigella sativa* seeds for the preparation of CDs that act as a dual sensor for tetracycline and ʟ-lysine. The fluorescence of CDs is quenched by adding tetracycline and regained by introducing ʟ-lysine [18].

Palmyra leaves were utilized by Athinarayanan et al. for the production of CDs. The cellular toxicity of the CDs was analyzed, and the CDs were found to have excellent biocompatibility with cells [70].

Ramanarayanan and Swaminathan utilized guava leaves to prepare CDs, which were then utilized for the synthesis of a CD-TiO_2_ nanocomposite. The CD-TiO_2_ nanocomposite possesses good photocatalytic ability to degrade methylene blue dye [71].

**Figure 3:**
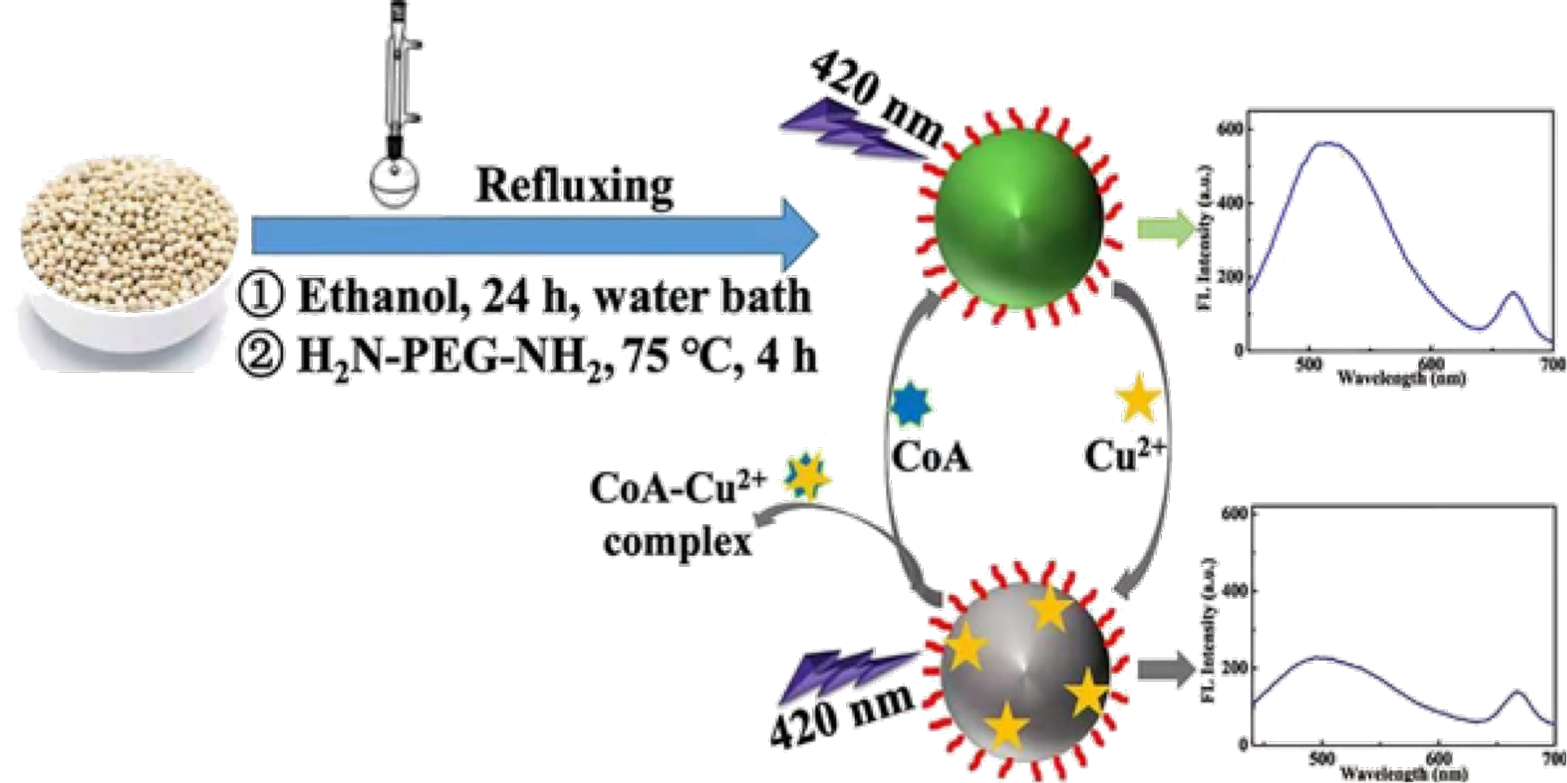
Strategy for the synthesis of ratiometric CDs obtained from white pepper. Figure 3 was reprinted from [86], Food Chemistry, vol. 315, by R. Long; Y. Guo; L. Xie; S. Shi; J. Xu; C. Tong; Q. Lin; T. Li, “White pepper-derived ratiometric carbon dots for highly selective detection and imaging of coenzyme A”, article no. 126171, Copyright Elsevier (2020), with permission from Elsevier. This content is not subject to CC BY 4.0.

White pepper as a natural precursor was reported by Long et al. to prepare CDs via refluxing in anhydrous alcohol for 24 h. The prepared CDs exhibit dual emission at 520 and 668 nm by using the same excitation wavelength of 420 nm. The red emission around 668 nm was hardly affected by metal ions and amino acids and acts as a reference while the green emission around 520 nm was quenched by Cu^2+^ ions and enhanced by CoA (Figure 3). The reported CDs were used as an “off–on” ratiometric fluorescence nanosensor for the detection of CoA [86].

Another green precursor, *Murraya koenigii* (curry leaves), was used to synthesize CDs via hydrothermal route. The reported CDs showed good selectivity for Cd^2+^ ions with a wide linear range and limit of detection of 0.01–8 μM and 0.29 nM, respectively [72].

Chaudhary et al. reported CDs using roasted gram as a green precursor, which act as a humidity sensor. Two different carbonization temperatures (200 and 450 °C) were used to prepare CDs, namely C-1 and C-2, respectively, via pyrolysis. To examine the interaction of functional groups present on the CDs with water vapor, theoretical modeling was also carried out by using the DFT-based B3LYP hybrid functional at 6–31G diffused and polarized basis sets. An excellent agreement was found between theoretical data and experimental results [73].

An ultrasonic-assisted solvent extraction approach was employed to synthesize CDs from rice fried *Codonopsis pilosula* (CP). The CP-CDs possess good sensitivity and selectivity towards the detection of Cr^6+^ ions. Linear range and limit of detection obtained were 0.03−50 μM and 15 nM, respectively [74].

Shekarbeygi et al. synthesized CDs from aqueous and alcoholic extracts of blue, yellow, and red rose flowers through a hydrothermal approach. They also studied the outcome of different synthesis methodologies on the optical properties of the prepared CDs. The CDs obtained from the alcoholic extract of yellow petals were more stable and had a high quantum yield. The reported CDs were efficiently employed for the detection of diazinon [75].

Flax straw was recently used for the synthesis of CDs via a hydrothermal method. The obtained CDs acted as fluorescence on-off-on sensors for the detection of Co^2+^ or Cr^6+^ ions and ascorbic acid, respectively [87]. Using a hydrothermal process, a new form of CD material was produced from common crop wastes, such as corn straw, wheat straw, and rice straw by Ding et al. These CDs were utilized to detect Fe^3+^, which could be useful in areas of environmental remediation and medical diagnosis [77]. A hydrothermal technique employing near-critical water has been utilized recently to develop a simple, cost-effective, and environmentally friendly synthetic route for CDs from corn stalk shell [78]. This process transformed biomass, which was previously thought to be waste material, into carbon nanomaterials with tremendous potential. Another method for the synthesis of biocompatible fluorescent CDs from the extract of leaves of the medicinal plant *Calotropis gigantea*, also known as crown flower was developed. The resulting CDs were applied as a fluorescent probe for bioimaging [80].

**With surface-passivating agents:** To obtain CDs with increased electron density, fluorescent properties, and QY, researchers are focusing on synthesizing CDs containing surfactants that contain nitrogen, phosphorus, or sulfur [16,88]. Usually, CDs synthesized from a single precursor without any doping agent have low QYs and are inadequate for bioimaging and other applications. Numerous researches claim that co-doping, or the simultaneous doping of two or more distinct atoms, can mitigate the drawbacks of CDs. The optical characteristics and applications of CDs may be enhanced by doping them with nitrogen, sulfur, and other elements. Due to the synergistic interaction between the doped heteroatoms in CDs, the co-doping with heteroatoms has started to attract greater attention since it can produce novel electronic structures. The electronic structure of CDs can be modified to produce n-type or p-type carriers by adding atomic impurities, such as nitrogen, boron, sulfur, or phosphorus. The QY of CDs could also be considerably enhanced by heteroatom doping, according to current research.

Xie et al. used ethylenediamine (EDA) as a surface-passivating agent to obtain hydrophilic N-CDs via a facile hydrothermal method from highland barley. The reaction was maintained at 200 °C for 24 h. A high QY of 14.4% was obtained by using an optimal amount of 1.33 mL of EDA. Because nitrogen-containing groups could passivate the surface-active sites of the CDs, a greater QY was obtained. Thus, the PL characteristics of the N-CDs were improved, and as a result, the QY of N-CDs was greater than that of the majority of N-CDs derived from biomass [89].

Bandi et al. reported N-CDs synthesized via a hydrothermal method by using *Lantana camara* berries and EDA as carbon and nitrogen source, respectively. The synthesis consisted of many steps, including hydrolysis, dehydration, and decomposition, through which carbohydrates and glycosides are converted into small molecules. The obtained molecules were then converted into N-CDs by passing through polymerization, aromatization, and carbonization processes. Optimized conditions of 180 °C, 3 h time, and 80 μL EDA yielded N-CDs with a QY up to 33.15% [90].

A very good QY up to 53% was obtained by using glutathione, as a dopant for both S and N, and a green source of celery leaves to synthesize CDs. Low-cost celery leaves containing folic acid with many –COOH and –NH_2_ groups contribute to high QYs. The reported CDs were novel fluorescent paper sensors and showed remarkable sensitivity and selectivity in the detection of nitrophenol [4].

**Figure 4:**
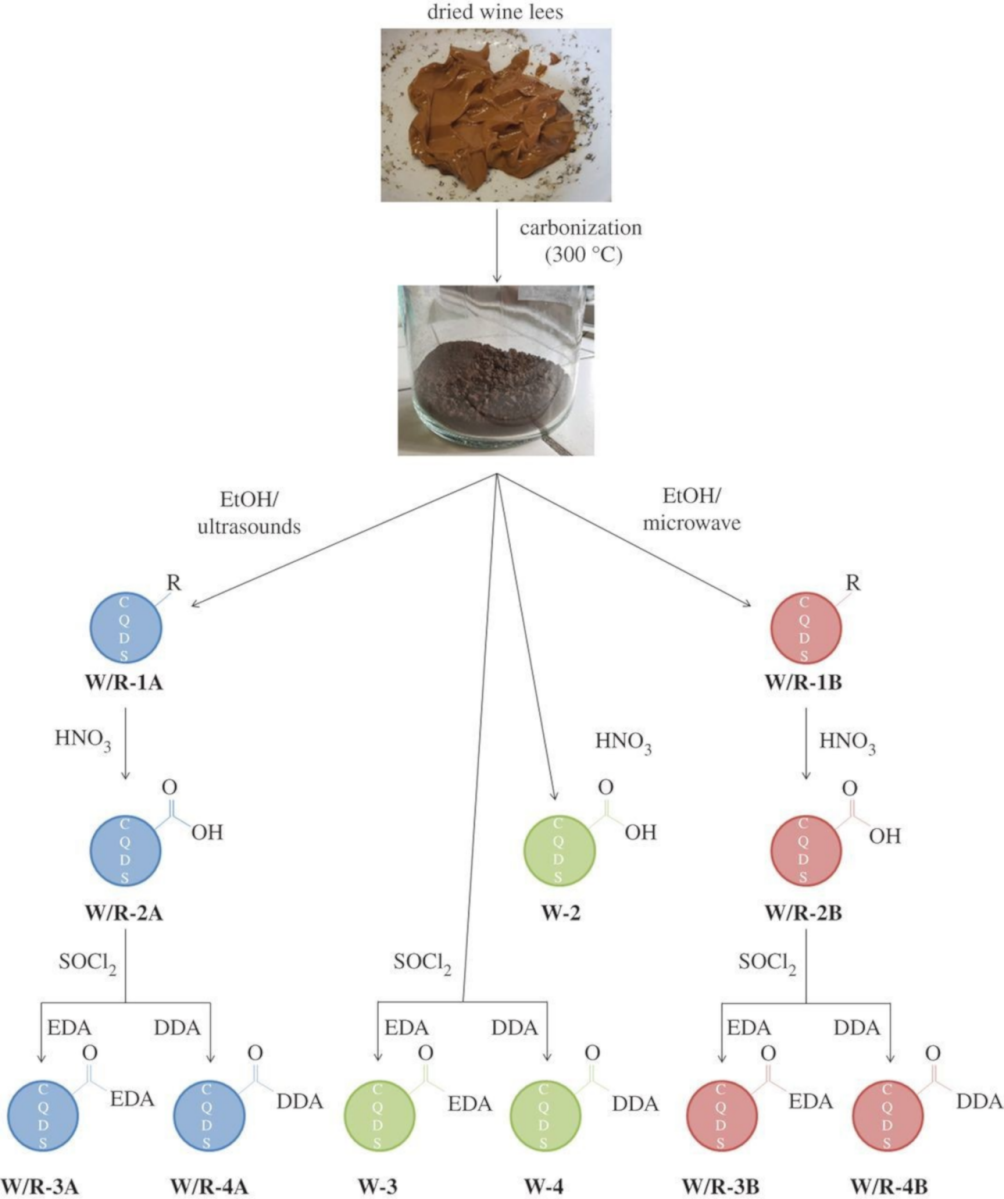
Graphical illustration of the procedure followed with wine lees. Figure 4 was reproduced from [91] (© 2017 M. Varisco et al., published by the Royal Society, distributed under the terms of the Creative Commons Attribution 4.0 International License, https://creativecommons.org/licenses/by/4.0).

Varisco et al. reported CDs from wine lees via combustion and used two extraction procedures, namely ultrasonication and microwave-assisted extraction, from the black mass. The wine lees consist of different compounds that can act as reactants for the preparation of CDs, that is, easily degradable organic compounds to make the CD core and long-chain carboxylic acids to act as passivating agents. To increase the number of carboxylic acids and amine-containing molecules on the CD surface, oxidation in HNO_3_ and reaction with SOCl_2_ were performed (Figure 4). The carboxylic groups react with EDA to increase the QY and with dodecyl amine (DDA) to obtain CDs dispersible in polar solvents [91].

CDs doped with nitrogen and sulfur (N,S-CDs) were synthesized using rose petals as a natural precursor, and ʟ-cysteine and EDA as N and S dopants, respectively, by Sharma et al. Cysteine and EDA help in hydrolysis and dehydration, respectively, which are part of the bottom-up approach. After completion of aromatization, the N,S-doped CDs are made from nuclear bursts [5]. Pal et al. used branched-chain PEI (bPEI) as a surface passivator and curcumin as a green precursor to synthesize hydrophilic CDs [92]. Aqueous ammonia was used as a nitrogen dopant for many natural source materials such as *Hylocereus undatus* (*H. undatus*), *Chionanthus retusus* (*C. retusus*), *Phyllanthus emblica* (*P. emblica*), and *Phyllanthus acidus* (*P. acidus*) to obtain N-CDs [93,96]. A good QY up to 31.7% was obtained by using EDA as N-dopant with orange juice as carbon source to give N-CDs via hydrothermal decomposition [17].

Das et al. have adopted a new strategy using κ-carrageenan as the carbon source and lemon juice as the sulfur source. Surface quaternization was performed with benzalkonium chloride to synthesize luminescent CDs. The surface functionalities were determined using small-angle neutron scattering, in which incident neutrons interact elastically with the specimen and give information about the surface and mass of the specimen [14].

Empty fruit bunch carboxymethylcellulose as carbon source and EDA as nitrogen dopant to synthesize N-CDs via one-pot hydrothermal carbonization approach have been used. Three different operating parameters, that is, synthesis temperature (230–270 °C), synthesis time (2–6 h), and EDA mass (10%–23.3%) were studied using response surface methodology. The highest values of temperature, time, and EDA mass were found satisfactory to get high QY values up to 22.9%. The factor that has the greatest influence on the QY was found to be the optimized temperature, followed by time and EDA mass [97]. Linear polyethyleneimines (LPEI) were used by the same group instead of EDA to synthesize fluorescent N-CDs and got an increase in QY up to 47% [98].

Blue luminous CDs, based on carrots and aqueous trisodium phosphate (TSP) as precursors, were prepared by a convenient reflux method. The obtained CDs were globular and about 3–8 nm in size as revealed by transmission electron microscopy [21].

Cassava peels as a natural carbon precursor and poly(ethylene glycol) (PEG) as a surface passivizing agent was used by Putro et al. to prepare CDs via the hydrothermal method [99]. Tu et al. used non-toxic fungal biomass *Ganoderma lucidum* along with EDA and diammonium hydrogen phosphate to synthesize water-soluble CDs and N,P-CDs through a facile hydrothermal method. The reported CDs and N,P-CDs presented high sensitivity and selectivity toward 2,4-dinitrophenol and 4-nitrophenol. The obtained QY for CDs and N,P-CDs were 3.54% and 11.41%, respectively [100].

Surendran et al. used honey, garlic, and ammonia as green source, sulfur source, and nitrogen source, respectively to prepare N,S-CDs via a simple hydrothermal technique. The Z-scan methodology was used for non-linear optical characterization and the agar well diffusion methodology was used to explore the antimicrobial performance of CDs against foodborne pathogens [101].

**Figure 5:**
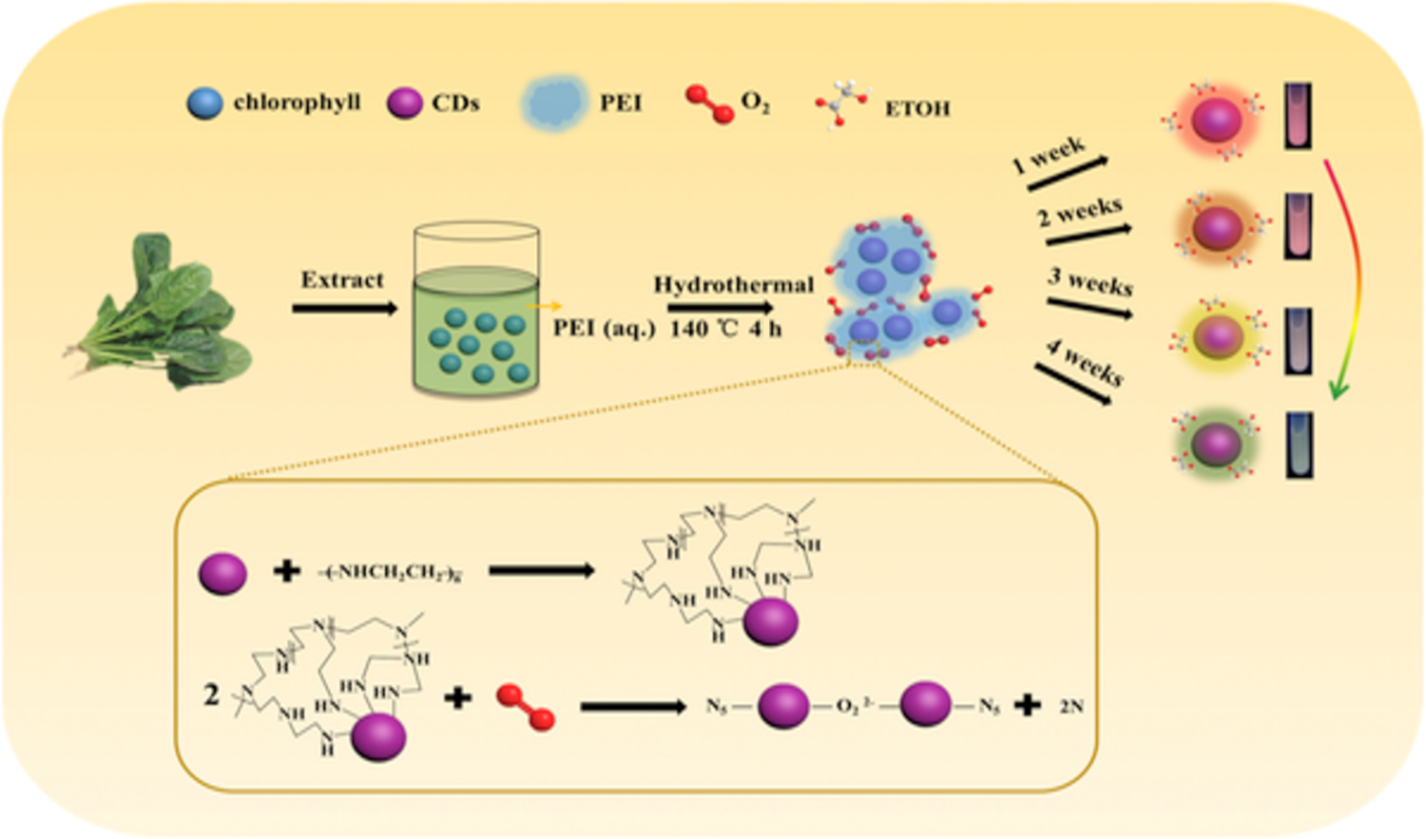
Schematic representation of the whole process. Figure 5 was reprinted from [102], Journal of Luminescence, vol. 227, by X. Xu; L. Cai; G. Hu; L. Mo; Y. Zheng; C. Hu; B. Lei; X. Zhang; Y. Liu; J. Zhuang, “Red-emissive carbon dots from spinach: Characterization and application in visual detection of time”, article no. 117534, Copyright Elsevier (2020), with permission from Elsevier. This content is not subject to CC BY 4.0.

Recently, a hydrothermal technique to build effective CDs using ginkgo kernels has been employed [82]. A unique technique was used to evaluate nitrites in corn sausage, ham sausage, preserved Szechuan pickle, and hot dog samples, yielding good results. In addition, the CDs had high water solubility, fluorescence stability, decreased cytotoxicity, and excellent biocompatibility with MCF7 cells, so they were also successful in bioimaging MCF7 cells. Xu et al. reported red emitting CDs using spinach as a natural source. PEI was used for the modification of the surface of CDs. They observed the change in PL characteristics of CDs during a period of one to four weeks and concluded that when amino-rich CDs come in contact with oxygen in the air, agglomeration of CDs is induced, and, hence, luminescence changes slowly from red to green color (Figure 5) [102]. A summary of CDs synthesized from carbon-rich plant extracts with different surface-passivating agents and their quantum yield is provided in Table 2.

**Table 2:** Summary of CDs synthesized from carbon-rich plant extracts with different surface-passivating agents and their quantum yields.

S. No	Precursor of CDs	Synthetic method	Surface-passivating agent	Size (nm)	QY (%)	Ref

1	celery leaves	hydrothermal method at 200 °C for 4 h	glutathione	2.08	53	[4]
2	*Rosa indica*	hydrothermal treatment at 180 °C for 5 h	EDA and ʟ-cysteine.	4.51–1.46	—	[5]
3	edible carrot	reflux	TSP	3–8	**—**	[21]
4	*Carica papaya*	pyrolysis	EDTA	7	23.7	[83]
5	table sugar	microwave assisted synthesis at 120 °C for 3 min	ammonia solution	3.5	2.5	[84]
6	white pepper	reflux method at 75 °C for 4 h	H_2_N-PEG-NH_2_	6.3	10.4	[86]
7	flax straw	hydrothermal method at 160 °C for 10 h	EDA	2.2	20.7	[87]
8	*Lantana camara* berries	hydrothermal 180 °C for 3 h	EDA	5 ± 3	33.15	[90]
9	curcumin	hydrothermal synthesis at 200 °C temp for 12 h	branched PEI (bPEI)	4–5	**—**	[92]
10	*Hylocereus undatus*	hydrothermal at 180 °C for 12 h	aqueous ammonia	2.5	**—**	[93]
11	*Phyllanthus emblica*	hydrothermal method at 180 °C for 12 h	aqueous NH_3_	4.08	**—**	[95]
12	*Phyllanthus acidus*	hydrothermal method at 180 °C for 8 h	aqueous ammonia	4.5 ± 1	14	[96]
13	orange juice	hydrothermal decomposition method at 200 °C for 4 h	EDA	0.5–3.0	31.7	[17]
14	oil palms empty fruit bunch	hydrothermal treatment at 260 °C for 2 h	polyethyleneimines (LPEI)	3.4	47	[97]
15	oil palms empty fruit bunch	hydrothermal treatment at 270 °C for 6 h	EDA	4.7	22.9	[98]
16	*Cassava* peels	hydrothermal at 160°C for 4 h	PEG	**—**	**—**	[99]
17	*Ganoderma lucidum*	hydrothermal treatment at 200 °C for 6 h	diammonium hydrogen phosphate and EDA	2.95 and 3.12	3.54 and 11.41	[100]
18	natural honey	hydrothermal method at 200 °C for 6 h	ammonia	8.29	≈4.192	[101]
19	spinach	hydrothermally method at 140 °C for 4 h	polyethylenimine (PEI)	3	**—**	[102]
20	*Lycii fructus*	hydrothermal treatment at 200 °C for 5 h	ammonia solution	3.3	17.2	[29]
21	*Chionanthus retusus*	hydrothermal-carbonization method at 180°C for 6 h	ammonia solution	5 ± 2	9	[94]

The majority of CDs have a low QY, which are insufficient for bioimaging and other applications. Heteroatom doping has been suggested as a way to enhance the fluorescence characteristics of CDs. Mostly, surface-passivating agents are used to dope CDs with nitrogen and phosphorus to increase their QY. Various plant extracts rich in such ingredients have been used. A list of the reported heteroatom-doped CDs made from natural materials is shown in Table 3. It can be categorized into three groups based on the distinct doped atoms: nitrogen, nitrogen/sulfur, and nitrogen/phosphorus. Hydrothermal treatment as well as pyrolysis, microwave heating, and acid oxidation have been extensively used to synthesize the heteroatom-doped CDs. Nitrogen-doped CDs have been found to have high QY because nitrogen atom doping helps to stabilize the surface defects of CDs and enhances fluorescence emission. Moreover, owing to its five valence electrons and an atomic size that is similar to carbon, nitrogen is a common dopant and the most frequently employed method of enhancing PL properties of CDs. By introducing electrons into CDs and altering the internal electronic states, nitrogen atoms significantly enhance the fluorescence characteristics of these molecules. The N-CDs produced perform exceptionally well in biomedical applications, including bioimaging and biosensing. A huge number of synthesis procedures have been investigated in order to produce CDs from environmentally friendly materials that contain nitrogen and carbon. *Magnolia liliiﬂora* was used to obtain N-CDs via an easy hydrothermal approach by Atchudan et al. The hydrothermal temperature and time used were 240 °C and 12 h, respectively. The obtained size was about 4 ± 1 nm and the quantum yield was up to 11% [103]. Huang et al. achieved an increase in QY up to 27% by using a bauhinia flower to synthesize N-CDs via a facile microwave method, without any further surface passivation or modification [104]. *Prunus cerasifera* fruit was used to synthesize highly luminescent CDs via a hydrothermal method. The reaction temperature and time used were 200 °C and 20 h, respectively. Characterization techniques such as TEM, FTIR, and XPS were used to study the CDs which were almost spherical and had high nitrogen content [49].

**Table 3:** Summary of doped CDs synthesized from plant extracts with their quantum yield.

S. No	Precursor	Synthesis method	Size (nm)	Doped atom	QY (%)	Ref

1	*Magnolia liliiﬂora*	hydrothermal treatment at 240 °C for 12 h	4 ± 1	nitrogen	11	[103]
2	*Bauhinia* flower	microwave, 1000 W for 10 min	3.4	nitrogen	27	[104]
3	*Prunus cerasifera*	hydrothermal method at 200 °C for 20 h	3–5	nitrogen	—	[49]
4	seaweed *(Sargassum fluitans)*	hydrothermal method at 180 °C for 5 h	2–8	nitrogen	18.2	[105]
5	watermelon juice	hydrothermal at 180 °C for 3 h	3–7	nitrogen	10.6	[16]
6	*Azadirachta indica*	hydrothermal treatment at 150 °C temp for 4 h	3.2	nitrogen	27.2	[106]
7	grass	hydrothermal at 180 °C for 3 h	3 to 5	nitrogen	4.2	[55]
8	banana peel waste	24 h at 200 °C	5	nitrogen	20	[79]
9	*Lonicera maackii*	hydrothermal process at 230 °C for 5 h	2–3	nitrogen	10.6	[107]
10	lily bulbs	microwave treatment for 6 min	3.15	nitrogen, phosphorus	17.6	[108]
11	*Dunaliella salina*	hydrothermal synthesis at 200 °C for 3 h	4.7	nitrogen, phosphorus	8	[109]
12	ginkgo leaves	hydrothermal treatment at 180 °C for 12 h	2.22	nitrogen, sulfur	—	[82]
13	*Allium fistulosum*	hydrothermal treatment at 220 °C for 3 h	4.22	nitrogen, sulfur	10.48	[110]
14	gardenia fruit	hydrothermal method at 180 °C for 5 h	2.1	nitrogen, sulfur	10.7	[53]

Godavarthi et al. used seaweed (*Sargassum fluitans*) to synthesize N-CDs. ^1^H NMR and ^13^C NMR were used to analyze *Sargassum fluitans* and showed that amino acids acted as a nitrogen source, for the preparation of N-CDs. The reported N-CDs were used as a fluorescence probe to detect DNA, and gel electrophoresis was used to compare its efficiency with some traditional organic fluorophores [105]. *Lycii fructus* was used by Sun et al. to develop an efficient method to prepare CDs by using a hydrothermal approach. The reported CDs showed good water solubility and excellent bio-compatibility, and the obtained QY was 17.2% [29]. Grass was used as a natural source to prepare immensely photoluminescent N-CDs through a hydrothermal method. Six different dyes, that is, acid blue, acid red, eosin Y, eriochrome black T, methyl orange, and methylene blue underwent degradation in the presence of radiation, which confirmed the catalytic activity of the product. Adsorption of heavy ions from water samples was investigated to find the activity on the surface of the product, and it was found that the mentioned CDs can remove Cd^2+^ ions by 37% and Pb^2+^ ion by 75% from the water sample [111]. Watermelon juice-based N-CDs with a calculated fluorescence QY of 10.6% were also reported, which were found to selectively detect Fe^3+^ ions. These N-CDs/Fe^3+^ systems could be used to sense cysteine, based on fluorescence “turn on” effects (see below Figure 9). The reported N-CDs showed temperature-dependent fluorescence behavior and were investigated to be used as a nanoscale thermometer for determining the intracellular temperature [16]. Numerous research groups have been actively examining co-doped N,S-CDs. Because the sulfur atom can supply energy or emissive trap states for photostimulated electron capture, which alters the electronic structure of CDs, N,S-CDs have drawn more interest in recent years. Li et al. reported a simple and economical one-pot hydrothermal carbonization route to prepare N,S-CDs by using ginkgo leaves as a natural precursor. XPS results demonstrated that the reported CDs were having elemental contents of 1.2% S, 60.4% C, 34.6% O, and 3.8% N [112]. Wei et al. reported N,S-CDs using *Allium fistulosum* as a green precursor. The prepared CDs revealed a diameter of about 4.22 nm by TEM and the QY obtained was up to 10.48% [110]. *Azadirachta indica* leaves was used to prepare fluorescent N-CDs by Yadav et al. The QYs obtained were as high as 27.2% [106]. Sun et al. used gardenia fruit as a green source to prepare N,S-CDs. The fluorescence intensity of the N,S-CDs was quenched by adding Hg^2+^ ions and recovered when cysteine was introduced to the system. Hence, an "on–off–on" sensor was developed that could detect Hg^2+^ and cysteine in a linear range of 2–20 μM and 0.1–2.0 μM for Hg^2+^ and Cys, respectively [53]. When compared to un-doped CDs, CDs co-doped with nitrogen and phosphorus (N,P-CDs) display novel and surprising features. After doping with N, the CDs become n-type semiconductors. In contrast to nitrogen, phosphorus atoms are larger than carbon atoms. As a result, it has the potential to act as an n-type donor and create substitutional defects in the carbon cluster, changing the electronic and optical characteristics of CDs with great impact on polarizability, quantum yield, and electrochemical properties. Lily bulbs as a green source to synthesize N,P-CDs via a facile, fast, and eco-friendly one-pot microwave-assisted method was reported by Gu et al. Lily bulbs rich in carbohydrates, proteins, lipids, and amino acids, can be easily used to prepare such CDs [108]. The microalgae *Dunaliella salina* was used to prepared low-cost N,P-CDs without any external agent. The algal biomass consists of amino acids and proteins, and XPS revealed that the CDs contain pyrrolic nitrogen and phosphate groups showing that these molecules were doped into the lattice of N,P-CDs [109]. Using banana peel waste as carbon and nitrogen source, novel CDs have been prepared using a simple hydrothermal carbonization technique in an ecofriendly approach by Atchudan et al. [79]. Their excitation-dependent fluorescence characteristics have been used successfully as a fluorescent probe in multicolor imaging applications of nematodes. Bi et al. described the synthesis of green fluorescent N-CDs from *Lonicera maackii* fruits using a one-step hydrothermal technique. The CDs could not only detect Fe^3+^ but also overcome the limitations of short-wavelength fluorescence CDs from natural materials, providing a basis for future applications in other disciplines [107]. Atchudan et al. reported a hydrothermal synthesis technique that yields CDs from *Morus nigra* (black mulberry) fruit juice [81]. This low-cost synthesis approach provides an effective, robust, and eco-friendly nanoscale sensor for the measurement of Fe^3+^. The CDs were also found to be appropriate FL probes for imaging human colon cancer (HTC-116) cells. Recently, Lin et al. proposed a unique antimicrobial compound for the preservation of Atlantic mackerel by synthesizing antimicrobial CDs through a hydrothermal synthesis approach using food materials as the precursor, including onion, ginger, garlic, and mackerel [113]. The sulfur content of the CDs based on onion and garlic was higher than that of the CDs derived from ginger and fish. Surprisingly, the onion-based CDs had the best antibacterial efficacy against *Pseudomonas fragi*, as well as good pH stability.

### Animal extract

**Figure 6:**
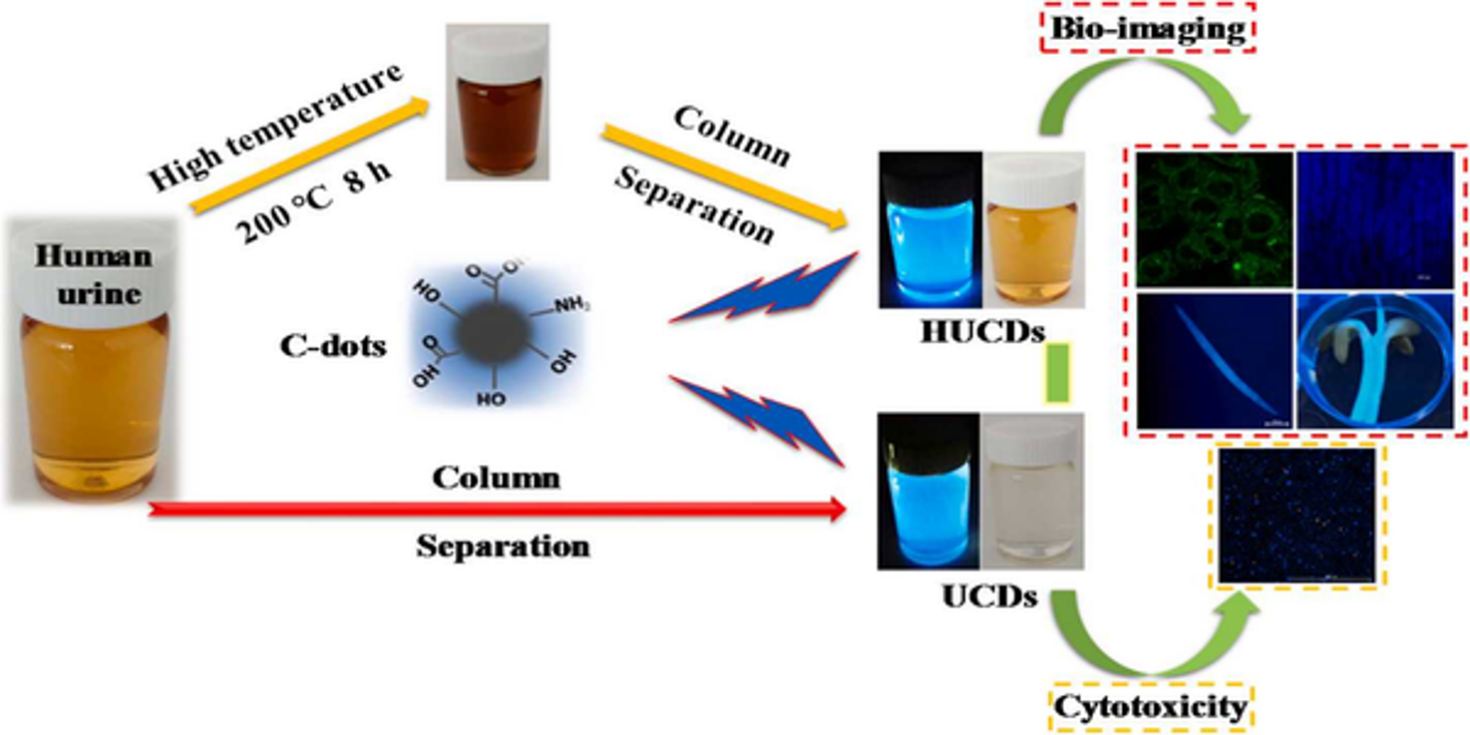
Graphical illustration for the synthesis of CDs from human urine and their application for bioimaging. Figure 6 was reprinted from [116], Methods, vol. 168, by X. Zhang; J. Li; J. Niu; X. Bao; H. Zhao; M. Tan, “Fluorescent carbon dots derived from urine and their application for bioimaging”, pages 84-93, Copyright Elsevier (2019), with permission from Elsevier. This content is not subject to CC BY 4.0.

Dehvari et al. converted seafood waste into valuable materials by using crab shell as a natural precursor to form highly fluorescent N-CDs by a sono-chemical approach. A N-CDs/folic acid nanoprobe was synthesized by conjugating N-CDs with folic acid, which could be used to target folate-receptor cancer cells [114]. Pork-based CDs without any surface-passivating agent were synthesized. Pork meat, itself, is composed of many organic molecules that can provide multiple heteroatoms in the CDs. The resulting CDs have excellent advantages such as a high QY of 17.3% and good stability to chemicals [115]. Zhang et al. obtained urine-based CDs through a simple sephadex filtration approach and a via a hydrothermal route (Figure 6). Different characterization techniques revealed that both types of CDs consisted of carbon and oxygen and indicated the existence of different functional groups such as amino, carboxylate, carbonyl, and hydroxy groups. The cytotoxicity study showed good biocompatibility and applicability in both in vitro and in vivo imaging [116].

Recently, milk was used by Al-Hashimi et al. as a natural precursor to synthesize N-CDs via a solvothermal method. These nanodots served as fluorophore in the inner filter effect (IFE) sensing platform to detect tetracycline in pharmaceutical doses [76]. Highly water-soluble and blue emitting CDs were synthesized from hemoglobin by Chakraborty et al., which can be used to detect H_2_O_2_ [117].

### Food materials

**Figure 7:**
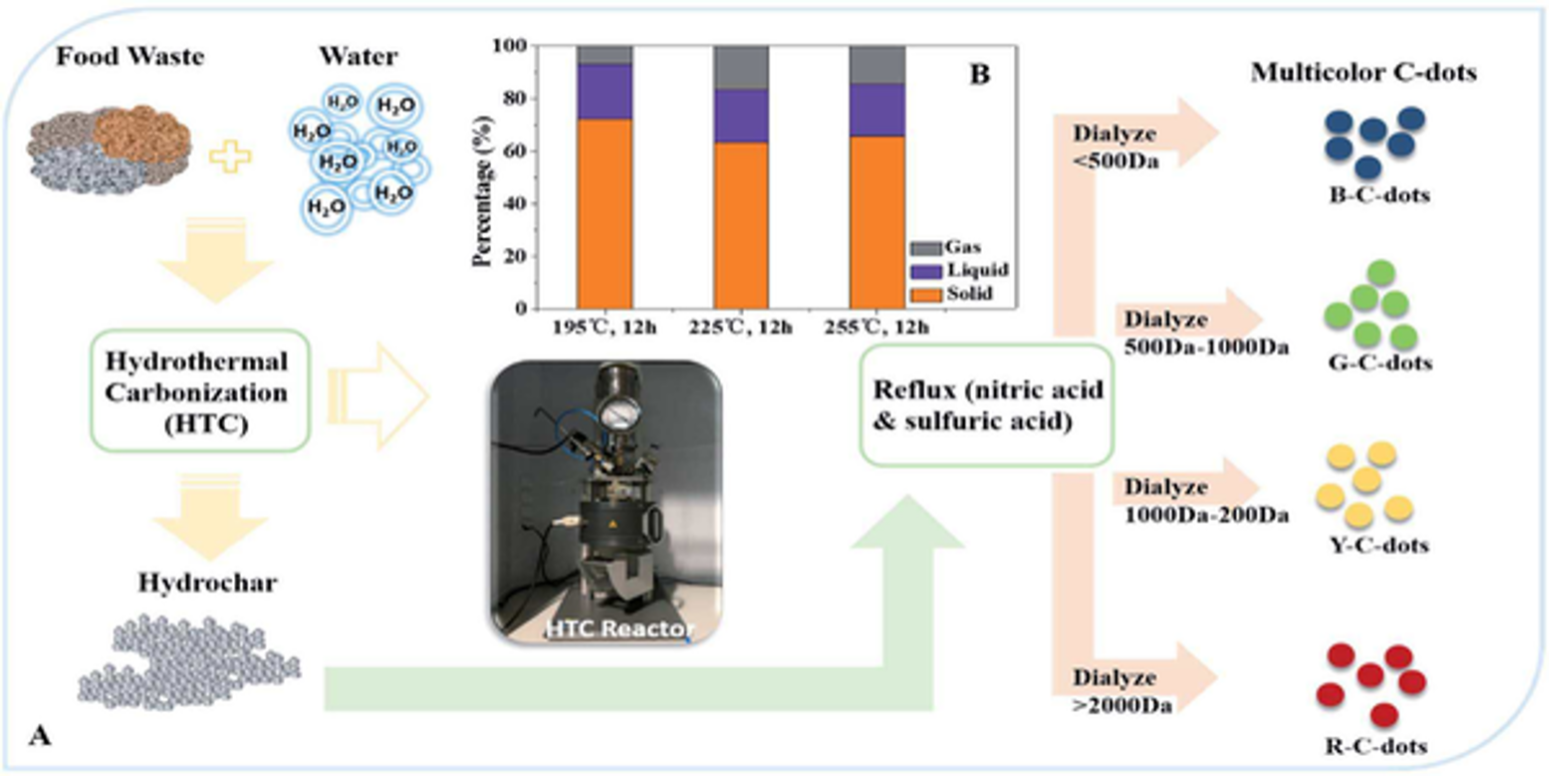
(A) Flow chart of multicolor CDs production. (B) Carbon distribution in gas, liquid, and solid phase from the new precursor production process. Figure 7 was reproduced from [118] (“Multicolor carbon nanodots from food waste and their heavy metal ion detection application“, © 2018 Y. Zhou et al., published by The Royal Society of Chemistry, distributed under the terms of the Creative Commons Attribution Noncommercial 3.0 Unported License, https://creativecommons.org/licenses/by-nc/3.0/). This content is not subject to CC BY 4.0.

Different food materials such as beans are also a good source of carbohydrates, which can be easily used to synthesize CDs. Hydrochar was produced from food waste via hydrothermal carbonization and utilized to synthesize CDs by Zhou et al. Specific temperatures (195, 225, and 255 °C) were used for 12 h, followed by the use of vacuum filtration to separate the hydrothermal product. The hydrochar was refluxed in nitric and sulfuric acid, and dialysis membranes of different sizes were used to get CDs emitting four different colors, that is, blue, green, yellow, and red (Figure 7A,B) [118]. EDTA was used in a solution containing different ions for quenching iron ions specifically.

Researchers have employed different beans to synthesize high-quality CDs. Jia et al. used black soya beans as a natural resource to prepare N-CDs by a one-step pyrolysis method. The obtained N-CDs had good photoluminescence characteristics. The reported QY was as high as 38.7 ± 0.64% [119]. Kaur et al. obtained a very high QY up to 58% by using EDA as nitrogen source with *Vigna radiata* (mung bean) to produce N-CDs. The reaction conditions EDA concentration and time were optimized to increase the QY. The concentration of EDA used per 10 mL of *Vigna radiata* extract ranged from 200 to 2000 μL, and the time range was from 5 to 36 h. A direct relationship between the concentration of EDA and the fluorescence intensity was found [120]. Khan et al. used red lentils as a carbon source in a simple, one-step, inexpensive preparation of water-soluble N-CDs. The reported N-CDs showed bright blue fluorescence under an ultraviolet lamp with a wavelength of 365 nm, and the QY was up to 13.2% [121].

Sesame as a natural source was also used to synthesize highly efficient CDs naturally doped with N, P and Ca ions by Yu et al. The reported CDs have a good QY up to 48.5% and emit strong blue fluorescence [122]. Soni et al. synthesized CDs, co-doped with nitrogen and sulfur, from palm shell powder as a natural precursor with trifilic acid. The obtained CDs had a graphite-like structure, a narrow size distribution, and showed intense green fluorescence. These CDs had fluorescence characteristics independent of the excitation wavelength and were used as fluorescent probes with LOD values of 0.079, 0.165, and 0.082 μM for 4-nitrophenol, 2,4-dinitrophenol and 2,4,6-trinitrophenol, respectively [123]. CDs synthesized from animal extracts and food materials, are summarized in Table 4.

**Table 4:** Summary of CDs synthesized from animals extracts and food materials, with their quantum yields.

S. No	Precursor	Synthetic method	Size (nm)	QY (%)	Ref

1	pork	hydrothermal method at 200 °C for 10 h	3.5	17.3	[115]
2	crab shells	ultra sonicationat 70 °C for 12 h	8	14.5	[114]
3	urine	urine based CDs (sephadex filtration method)	2.5	4.8%	[116]
		urine based CDs (hydrothermal method) 200 °C for 8 h	5.5	17.8%	
4	hemoglobin	muffle furnace 120 °C for 1 h	≈4.0	≈73	[117]
5	black soya beans	pyrolysis at 200 °C for 4 h	5.16 ± 0.30	38.7 ± 0.64	[119]
6	*Vigna radiate*	hydrothermal at 180 °C for 24 h	<10	58	[120]
7	red lentils	hydrothermal at 200 °C for 5 h	6	13.2	[121]
8	sesame	hydrothermal process at 150 to 200 °C for 1–5 h	25	48.5	[122]
9	palm shell	hydrothermal method at 150 °C for 10 h	4–10	—	[123]

### Natural polymer-based CDs

Numerous natural polymers, including proteins and polysaccharides, have been used to obtain CDs (Table 5). Chen and co-workers used starch as a source to synthesize graphene quantum dots (CDs) via a one-pot hydrothermal method. They also described the reaction mechanism, which involves hydrolyzation of starch to form glucose, and then glucose is condensed to form CDs by ring-closure condensation. These CDs have been effectively used in bioimaging of cervical cancer cells as a suitable PL probe [124]. Wen et al. have created an incredibly eco-friendly and cost-effective method for creating highly luminous CDs from cotton by pyrolysis and microwave treatments. The CDs exhibit high fluorescence QY, great biocompatibility, low toxicity, and adequate stability. The CDs have found applications in various fields, including multicolor imaging, patterning, and sensing, as a result of their advantageous characteristics [125]. Han and co-workers extracted cow milk-derived CDs (CM-CDs) from aqueous solution using ethyl acetate to create amphiphilic CM-CDs (ACMCDs). A unique ACMCD-Ag/polymethylmethacrylate antibacterial film was produced utilizing the solvent casting process after the ACMCDs were supported by silver nanoparticles, employing them as both a reducing agent and a template. The nanocomposite antibacterial film is anticipated to have a lot of potential applications such as food packaging, water purification, and disinfecting sanitary equipment because of its superior antibacterial, light-admitting, and flexible features [28]. In another report, CDs were prepared from egg white using a one-step hydrothermal method. Carbonization, N-doping, and surface functionalization all occurred simultaneously during the hydrothermal reaction. The CDs were employed as probes for detecting metal ions and in live-cell imaging, and they had a high quantum yield of 61% [126]. Chen et al. proposed a quick method for producing highly luminous CDs by hydrothermally treating lignin with H_2_O_2_. It is well recognized that under the photoassisted catalysis of Fe^3+^/Fe^2+^ in water, H_2_O_2_ can be split into hydroxyl radicals, and the ensuing radical is an incredibly potent oxidizing species. The treatment time lasted from 10 to 60 min, with 40 min yielding the highest CD luminescence. The resulting CDs (2–10 nm) have excellent penetration into HeLa cells, minimal cytotoxicity, high water solubility and photostability [127]. Fiber-rich peanut shells were used to produce fluorescent CDs (1.8–4.2 nm) that had a QY of 10.6% and better photostability than rhodamine B. The CDs were produced using a straightforward one-pot pyrolysis procedure at 400 °C for 4 h, and they were effectively used for Cu^2+^ detection, which was attributed to the fluorescence quenching effect of the ions [128]. A unique and quick microwave-assisted method that requires two steps was reported by Pires for the synthesis of CDs with an average size of 9 nm from an aqueous solution of a polysaccharide, that is, raw cashew gum. Through the autohydrolysis of cashew gum, some monomer units are produced in the first phase (partial depolymerization in solution), and a trace amount of 5-hydroxymethyl furfural can be produced. The formation of a polyfuranic structure through polycondensation and polymerization is followed by aromatization, carbonization, and nuclear fusion in the second phase. As a result, a composite of partially depolymerized cashew gum and CDs was produced [129]. Wu and co-workers used *Schisandra chinensis* polysaccharide as carbon source that also has a naturally nitrogen-containing structure for endogenous nitrogen-doping in a green synthesis of CD-based room-temperature phosphorescent (RTP) materials. The materials had lifetimes of up to 271.2 ms with a lower energy gap and 350 nm of excitation (0.32 eV). Furthermore, when iron ions (Fe^3+^) were introduced, they displayed appropriate quenching. Additionally, the generated CD-based RTP materials have extremely stable optical and physical characteristics, opening up a new avenue for using them as low-cost, environmentally friendly luminescent sensors for Fe^3+^ detection [130].

**Table 5:** Summary of CDs synthesized from various polymers, with their quantum yields and applications.

S. No.	Precursor of CDs	Synthetic method	Size (nm)	QY (%)	Application	Ref

1	starch	hydrothermal, 190 °C, 120 min	2.50–2.75	—	bioimaging	[124]
2	cotton	pyrolysis, 300 °C for 2 h	4.9 ± 0.10	14.8	multicolor imaging, patterning, and Fe^3+^ detection	[125]
3	cow milk	hydrothermal, 180 °C, 12 h	1–5	—	antimicrobial	[28]
4	egg white	hydrothermal, 220 °C, 48 h	2.1	61	Fe^3+^ detection bioimaging, optical devices	[126]
5	lignin	hydrothermal, 180 °C, 40 min	2 to 10	—	bioimaging	[127]
6	peanut shells	pyrolysis, 400 °C, 4 h	3.3	10.6	Cu^2+^ detection	[128]
7	raw cashew gum	microwave (800 W), 30–40 min	9 ± 3	8.7	—	[129]
8	*Schisandra chinensis*	hydrothermal, 200 °C, 8 h	2.31	—	Fe^3+^ detection	[130]

### Surface state effects on the optical features of CDs

The surface state of CDs, which is closely linked to their fluorescence emission and serves as the most commonly acknowledged luminescence mechanism until now, includes the level of surface functional groups, surface oxidation, and molecular fluorescence.

**Surface oxidation level:** Numerous investigations have demonstrated that the level of surface oxidation in CDs is what causes their luminescence. The amount of oxygen on the surface of CDs directly influences its redshifted emission. The number of surface defects increases with the surface oxidation level. The surface defects trap excitons, and the radiation from the recombination of trapped excitons causes the redshifted emission. Liu et al. reported the synthesis of highly photoluminescent CDs, which were then further separated into yellow emitting crystalline graphene quantum dots and green emitting amorphous carbon nanodots using a silica gel column. Even though they have the same chemical surface groups and particle size distribution, they both have varying levels of surface oxidation. The emission wavelength moved from 518 to 543 nm as the degree of surface oxidation increased, which gives an indication about the reduction of band gap between LUMO and HOMO [50].

**Surface functional groups:** A correlation between the surface states and the functional groups found on the surface of CDs has been established by many researchers. Functional groups such as C=O and C=N are strongly associated with the fluorescence of CDs. Diverse fluorophores or energy levels can be introduced into CDs by different surface functional groups. Wang et al. demonstrated that by adjusting the functional groups on the surface of CDs, the emission wavelength could be dramatically altered. In some unique edge states, which were made up of many carbon atoms and functional groups (such as carbonyl) on the edge of the carbon backbone, the emission centers of CDs were equally distributed. Therefore, by affecting the emission centers, the functional groups on the surface of CDs may ultimately alter the fluorescence [131].

**Molecular fluorescence:** According to recent research, most of the emission from CDs is caused by fluorescent impurities, which are produced during the bottom-up chemical synthesis, or molecular fluorescence. Yang et al. proved that 1,2,3,5-tetrahydro-5-oxo-imidazol[1,2-a]pyridine-7-carboxylic acid (IPCA), which is a fluorescent molecule, was responsible for the bright blue fluorescence of CDs synthesized using citric acid and EDA. It has been established that the CDs are a mixture of IPCA, polymers, and carbon cores [132]. Essner and co-workers carried out the synthesis of CDs using citric acid (paired with urea or ethylenediamine as a nitrogen source). They showed that luminous impurities formed as byproducts during the synthesis of CDs mostly contribute to fluorescence emission by eliminating the molecular fluorophores [133]. By using fluorescence correlation spectroscopy and time-resolved electron paramagnetic resonance spectroscopy, Righetto and co-workers proved that free molecules are the source of the fluorescence in CDs. In the excitation range from 320 to 450 nm, CDs were produced by the emission of small fluorescent molecules dispersed in solution. In contrast, poorly emitting carbon cores predominate in the emission at excitation levels above 480 nm. Since small organic molecules are free in solution, it follows that even when carbon cores do exist, the origin of the fluorescence is attributed to these molecules [134].

### Characterization of CDs

**Figure 8:**
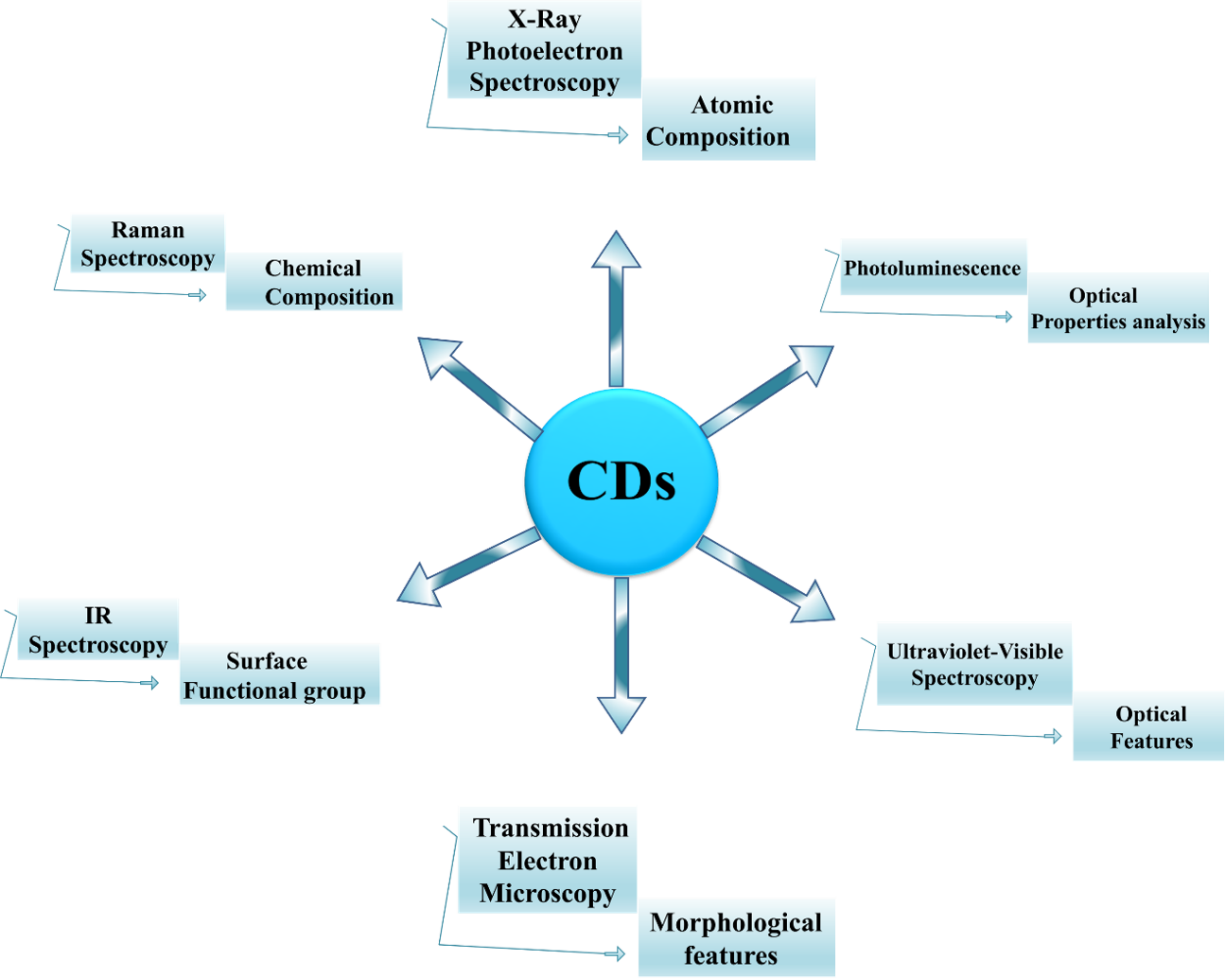
Structural characterization techniques used to study CDs.

There are several published analytical methods for characterization, illustrating physical characteristics, demonstrating the crystalline structure, and determining type and sufficiency of functionalization groups attached on the surface of the CDs (Figure 8). A brief description is provided in this review.

#### Transmission electron microscopy

Because of its great resolution of 0.1–0.2 nm, TEM can be used to determine the morphology of CDs. Since CDs have a particle size of less than 10 nm, high-resolution techniques such as TEM are frequently used to investigate the morphology and the particle size distribution. High-resolution TEM (HR-TEM) can also be utilized to examine the intricate structure of the CDs. HR-TEM can reveal the fine structure of CDs and whether they are crystalline or amorphous. Additional morphological data can be obtained using SAED patterns. The phrase “interlayer spacing” is used to describe lattice fringes produced through HRTEM analysis with a fringe width spacing around 0.34 nm, whereas the term “in-plane lattice spacing” is used for a fringe width of 0.24 nm.

#### Photoluminescence

Due to the vast range of applications, photoluminescence, which results from the quantum confinement effect, is now regarded as the most intriguing aspect of CDs. Because of their unique optical characteristics, CDs may be able to reflect impacts from particles of different sizes, shapes, internal structures, and compositions. CDs also has a variety of emissive sites spread across the surface. The emission spectrum of CDs typically ranges from the visible to the near-infrared region, with a characteristic red shift as the excitation wavelength increases, which is regarded as a remarkable characteristic of the PL of CDs. Despite a lot of research in this area, the precise mechanism of PL emission in CDs is still unknown.

#### Fourier-transform infrared spectroscopy

FTIR spectroscopy is a method for determining the presence of functional groups on the surface of CDs that is based on the detection of electromagnetic radiation absorption at wavelengths between 4000 and 400 cm^−1^. The majority of the CD surfaces are rich in hydroxy, carbonyl, ether, and carboxylic acid groups because the synthesis process involves partial oxidation of the carbon source [135]. Therefore, exploration of functional groups is essential for characterization. Using elemental doping and composite fabrication, the structure of CDs has been modified to enhance analytical performance. The degree of surface passivation can be assessed using FTIR analysis.

#### Ultraviolet–visible spectroscopy

CDs made using a variety of procedures often exhibit strong UV absorption. However, the positions of the UV absorption peaks change significantly depending on the synthesis method. In general, CDs show strong optical absorbance in the UV range between 260 and 320 nm, with a tail that extends into the visible spectrum. Pure CDs often contain two absorption peaks, that is, one for the n–π* transition of surface functional groups such as carbonyl, hydroxy, ester, and carboxyl groups, and one for the π–π* transition of aromatic sp^2^ domains [38]. However, surface groups and synthetic process have a significant impact on where the absorption peaks are located. Doping heteroatoms or incorporating them into a composite material has a significant impact on the absorption wavelength of CDs because of the change in the π–π* energy level [136]. The commonly occurring broad absorption spectra of CDs are caused by the surface defects.

#### X-ray photoelectron spectroscopy

For surface chemical investigation and characterization of nanoscale materials, X-ray photoelectron spectroscopy is an effective analytical tool. The electrical structure, elemental composition, and oxidation states of the elements in a material can all be determined using this method, which depends on the photoelectric effect [137]. Surface chemical modification, core–shell topologies, and various components of CDs can all be investigated using XPS.

#### Raman spectroscopy

Raman analysis is frequently used to measure the vibrational modes and provide structural data about molecules. Raman analysis can be used to assess the optical and electrical characteristics, crystalline or amorphous nature, and other characteristics of CDs. Usually, CDs exhibit two distinctive Raman peaks called the D band and the G band. Owing to the oscillations of vastly disordered sp^2^-hybridized graphitic dangling bonds on the termination plane, the D band is typically detected at a wavelength of about 1352 cm^−1^. The E_2_g mode of the 2D hexagonal lattice of graphite correlates to the vibrations of the sp^2^-linked carbon atoms that cause the G band, which is visible at about 1585 cm^−1^ [27].

### Applications as metal ion sensors

As CDs possess unique photoluminescence and excitation-based emissions, quenching and reversal of quenching processes can be used as an analytical tool for detection of certain analytes. CDs have been widely used as sensors for metal ions such as Fe^3+^ [49,59,85,138–141], Fe^2+^ [25], Hg^2+^ [109,142–143], Cu^2+^ [86,108,144–146], Cd^2+^ [147], Cr^6+^ [52,148–152], Co^2+^ [153], Au^3+^ [154], As^3+^ [155–156], Pb^2+^ [157], and Ag^+^ [158]. This sensing application is based on the principle that fluorescence is either quenched or increases when the functional groups on the surface of the CDs interact with the metal ions creating new electron–hole pairs via energy transfer. Different sensing/quenching mechanisms include inner filter effect (IFE), fluorescence resonance energy transfer (FRET), electron transfer (ET), static quenching effect (SQE), dynamic quenching effect (DQE), aggregation-induced emission enhancement (AIEE) effect, and aggregation-induced emission quenching (AIEQ) effect.

**Static quenching** occurs when fluorophore/CDs interact with the quencher and form a ground non-fluorescent complex [54,94,119].

**Dynamic quenching** occurs when an excited state of CDs returns to ground state by energy/charge transfer between the quencher and the CDs. This quenching only affects excited states, and, hence, no difference is observed in the absorption spectra of CDs [89,97,104].

**Fluorescence resonance energy transfer (FRET)** is a process where photonic energy of a fluorophore/CDs is transferred to another fluorophore/CDs, which then transmits it. FRET occurs between CDs and quencher in their excited and ground state, respectively, when the emission spectrum of CDs overlap with the absorption spectrum of the quencher [19].

**The inner filter effect (IFE)** occurs when there is a good spectral overlap between the absorption spectrum of the quencher and the excitation/emission band of the fluorophore/CDs [113,120].

**Electron transfer (ET)** may cause quenching when metal ions combine with various functional groups on the surface of CDs and lead to electron transfer from CDs to the ions. CDs act as electron source and generate excited electrons on irradiation, which are then transferred to the electron-deficient metal ions, and, hence, quenching occurs [109].

**Aggregation-induced emission enhancement effect (AIEE)** may arise as a result of aggregation of CDs. The aggregation increases the π conjugation and hinders the rotational vibration of the functional groups, which enhances the radiative rate and lowers the non-radiative rate.

**Aggregation-induced emission quenching effect (AIEQ)** occurs when fluorescent CDs aggregate and the energy of the excited fluorophores is transferred to the ground-state fluorophores without radiative transition, leading to a decrease or even complete quenching of fluorescence [154].

Detection of metal ions and efficient monitoring of their concentrations in aqueous and various biological samples is important because of their biological role and the toxicity, above certain thresholds, to the environment, human health, plants, and animals. Detection through CDs is advantageous because of the ease of operation, and high selectivity and sensitivity.

#### Fe^3+^ ion sensing

Fe^3+^ ions are one of the most abundant metal ions and play a vital role in biological systems as well in environmental systems. Fe^3+^ ions participate in biological processes such as metabolism, electron transfer systems, and oxygen supply of hemoglobin. They accumulate in the form of ferritin in liver and spleen. Both, deficiency and excess of the ions lead to serious disorders, such as certain kind of cancers or anemia. Hence, it is essential to monitor the Fe^3+^ levels in the environment and in biological systems. Various CDs are sensitive and selective towards Fe^3+^ ions [97–99].

Highly stable and water-soluble CDs, prepared from *Boswellia ovalifoliolata* by hydrothermal carbonization, exhibited high selectivity towards Fe^3+^ ions through quenching, with a LOD of 0.41 µM, in addition to useful applications such as free radical scavenging and bioimaging. The quenching is believed to proceed through a dynamic quenching mechanism. Groups such as –OH and –COOH on the surface of CDs facilitate complexation with Fe^3+^ ions, electron transfer occurs from the excited state of the CDs to unoccupied orbitals of Fe^3+^ and, hence, quenching occurs [138].

*Citrus limetta*-based CDs synthesized through pyrolysis, were found to have a wide range of applications. The CDs performed well in sensing Fe^3+^ ions with a LOD of 19.8 ppb. Fluorescence quenching was observed, which was explained based on the presence of –COOH groups on the CDs having a high affinity for the metal ions, which leads to static quenching. Composites of these nanoscale lights or CDs with TiO_2_ nanofibers were found to be efficient for water splitting application. They also exhibited fast catalytic degradation of methylene blue, bactericidal behavior, and their general non-toxicity makes them good candidates for bioimaging as well [139].

**Figure 9:**
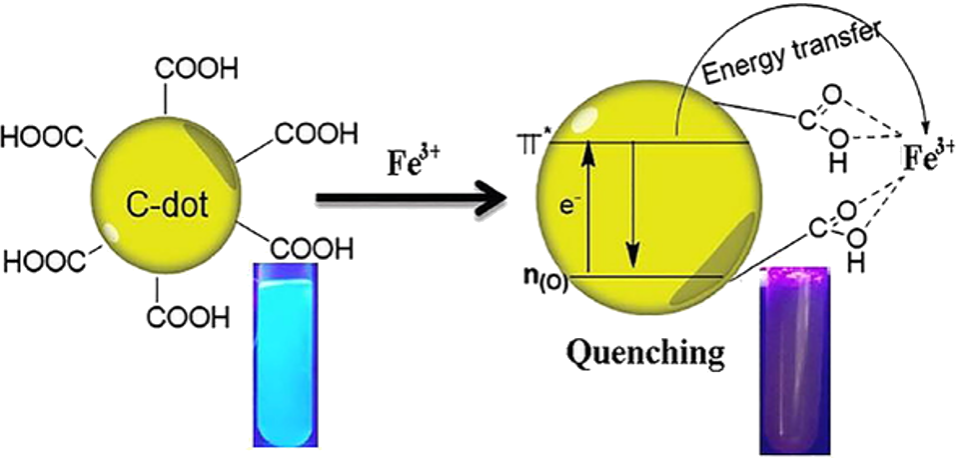
The proposed quenching mechanism of CDs prepared from betel leaves. Figure 9 was reprinted from [140], Materials Today: Proceedings, vol. 34, by D. Raja; D. Sundaramurthy, “Facile synthesis of fluorescent carbon quantum dots from Betel leafs (Piper betle) for Fe^3+^ sensing”, pages 488-492, Copyright Elsevier (2021), with permission from Elsevier. This content is not subject to CC BY 4.0.

Fluorescent CDs synthesized from betel leaves exhibit selective detection of Fe^3+^ ions with a LOD of 50–150 nM leading to the quenching of their fluorescence activity. The quenching process was explained based on charge transfer of –COOH on the CDs to the ferric ion (Figure 9) [140].

CDs and doped CDs (phosphorus and nitrogen) have been obtained from *Miscanthus grass*, where the N-doped CDs have the potential to selectively detect Fe^3+^ ions in the presence of other ions, with a LOD of 20 nM. Blue fluorescence was observed under UV, which was quenched when Fe^3+^ is added, and was explained based on a strong affinity of the metal ions to coordinate with amino and –COOH groups of the N-doped CDs. All CDs and doped CDs were found to exhibit excitation-dependent emission in addition to fluorescence [141].

Gupta et al. synthesized CDs from pine apple (*Ananas comosus*) through an acid oxidation approach. The reported CDs exhibited three colors under UV light, that is, blue (B-CDs), green (G-CDs), and yellow (Y-CDs). It was found that only B-CDs were sensitive to Fe^3+^ ions. The functional groups present on the B-CD surface coordinates with Fe^3+^ ions resulting in dramatic changes in the morphology and size of the B-CDs. From analyzing HR-TEM images and fluorescence spectra, it was concluded that fluorescence detection of Fe^3+^ occurs because of aggregation of B-CDs and the IFE [85].

Huang et al. used bauhinia flowers to synthesize N-CDs, which acted as a turn-off/on fluorescence sensor for the detection of Fe^3+^ and adenosine triphosphate (ATP), respectively. They described that the fluorescence quenching (turn-off) in case of Fe^3+^ ions occurs due to coordination of Fe^3+^ with surface functional groups of N-CDs, which resulted in non-radiative electron/hole recombination. Recovery of the fluorescence intensity (turn-on) by ATP is due its interaction with Fe^3+^ via Fe–O–P bonds [104]. The same mechanism for Fe^3+^ ion detection was also described by Ma et al. for CDs prepared via a hydrothermal route using *Prunus cerasifera* fruit as a carbon source [49].

Murugan and Sundramoorthy used *Borassus flabellifer* (male tree) as raw material to obtain CDs and used them to detect Fe^3+^ ions. Fe^3+^ have a 3d^5^ electron configuration. Thus, excited-state electrons of CDs can move to the half-filled 3d orbital of Fe^3+^ ions via coordination interaction resulting in non-radiative electron/hole recombination and fluorescence quenching [59].

The N-CDs prepared from water melon juice by Lu et al., acted as fluorescence turn-off/on sensor to detect Fe^3+^ and cysteine, respectively. The selectivity concerning Fe^3+^ was assigned to coordination of Fe^3+^ ions with hydroxy, amine, and carboxyl functional groups present on the N-CD surface resulting in interruption of radiative transition and fluorescence quenching. They used the N-CDs/Fe^3+^ system to sense cysteine because cysteine can compete with N-CDs regarding Fe^3+^ ions and lead to their removal and, hence, recovery of the fluorescence [16].

CDs derived from grapes and onions, which were found to have a reducing ability, were reported and employed for the determination of Fe^3+^. The CDs reduce Fe^3+^ ions to Fe^2+^ and, hence, provide a colorimetric approach for Fe^3+^ detection. The reducing ability of grape-based CDs was better than that of the onion-derived CDs [159].

Starch fermentation wastewater was used for the synthesis of S,N-CDs that showed selectivity towards Fe^3+^. It was investigated that the fluorescence of the S,N-CDs was quenched mainly because Fe^3+^ is a strong electron acceptor and, therefore, captures the excited electrons from the CDs with surface functional groups. The fluorescence quenching was reversed through the addition of phosphate at pH 7 [160].

Devi et al. prepared *Aloe vera*-derived CDs with Fe^3+^ sensing ability. Their selectivity towards Fe^3+^ is due to the presence of –COOH and –OH groups on the surface of the CDs. The fluorescence quenching was observed to decrease with increasing Fe^3+^ concentrations linearly [67].

Fluorescent N-CDs were synthesized by Atchudan et al. using *Magnolia liliiflora* as a carbon source, which exhibited excellent sensing towards Fe^3+^. The N-CDs were investigated to have a high number of nitrogen- and oxygen-containing functional groups. The sensing of Fe^3+^ was attributed to a mechanism where N-CDs get complexed with Fe^3+^ through functional groups, quenching the fluorescence [103].

Arumugham et al. developed carbon quantum dots using leaves of white *Catharanthus roseus* through a one-pot hydrothermal method, without employing any oxidizing agents and surface passivation. These water-soluble carbon quantum dots were sensitive towards Al^3+^ and Fe^3+^ ions. Quenching occurs when electrons are transferred from the excited CD to the empty orbitals of Fe^3+^, leading to non-radiative electron–hole recombination. With Al^3+^, the fluorescence increases as the ions coordinate with the CDs forming aggregates leading to aggregation-based high photoluminescence emission [161]. Antioxidant potential against 2,2-diphenyl-1-picrylhydrazyl was also measured and was found to improve with increasing concentration of the CDs. MCF-7 cells were treated with CDs and in vitro bioimaging was carried out using fluorescence microscopy under UV excitation. However, no significant cell decline was observed. In addition, they demonstrated non-toxicity towards both breast cancer cell lines and normal breast epithelial cells.

Bi et al. synthesized green fluorescent CDs from *Lonicera maackii* fruit through a hydrothermal process, which were efficient in Fe^3+^ detection in actual water samples with a LOD of 0.1–10 µM. The short fluorescence time indicated rapid electron transfer from the excited state to the empty d orbitals of Fe^3+^ causing quenching. Besides, the CDs have phenolic hydroxy groups containing lone pairs of electrons, which, on reaction with Fe^3+^, get transferred to its empty d orbitals resulting in non-radiative electrons/hole recombination. This process causes a further decrease in fluorescence [107]. Similarly, Ding et al. prepared CDs from typical crop waste such as wheat straw, corn straw, and rice straw using hydrothermal methods for the detection of Fe^3+^, displaying similar properties [77].

#### Fe^2+^ ion sensing

Shi et al. used cornstalk as a green precursor to prepared CDs for the detection of both Fe^2+^ ions and H_2_O_2_. They observed no change in fluorescence intensity when Fe^2+^ and H_2_O_2_ were added to an aqueous solution of CDs indicating that there was no effect on either Fe^2+^ or H_2_O_2_ alone. A significant decrease in fluorescence intensity was only observed when both were added together. The surface of prepared CDs is composed of plenty of hydroxy and oxygen functional groups. Thus, the reduction in fluorescence of CD was mainly attributed to the formation of metal hydroxide complexes on the CD surface with quenching probably occurring via electron or energy transfer with surface of CDs. They designed a CD–H_2_O_2_ system for Fe^2+^ ion detection and a CD–Fe^2+^ system for H_2_O_2_ detection and obtained LOD values of 0.18 and 0.21 μM for Fe^2+^ and H_2_O_2_, respectively [25].

#### Hg^2+^ ion sensing

**Figure 10:**
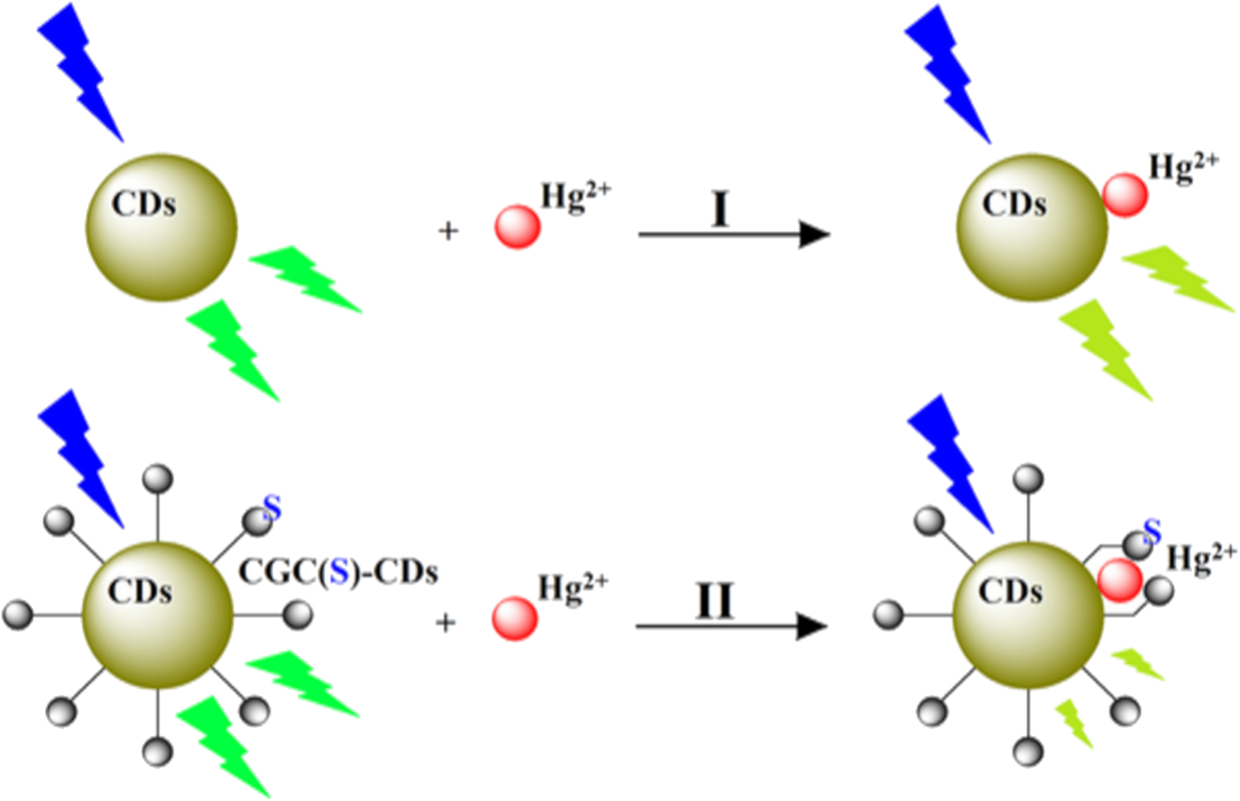
Detection mechanism of Hg^2+^ by using the CGCS-CDs as fluorescence probe. Figure 10 was reprinted from [142], Microchemical Journal, vol. 145, by G. Liu; H. Jia; N. Li; X. Li; Z. Yu; J. Wang; Y. Song, “High-fluorescent carbon dots (CDs) originated from China grass carp scales (CGCS) for effective detection of Hg(II) ions”, pages 718-728, Copyright Elsevier (2019), with permission from Elsevier. This content is not subject to CC BY 4.0.

Mercury is a heavy metal and highly toxic to humans and the environment. High levels of Hg^2+^ severely damage the central nervous system and other vital organs, and even lead to death. Mercury appears in Hg^2+^ form in water. Besides, elemental and organic mercury compounds are also found in polluted waters. The allowed level in drinking water is 1 µg/L. Liu et al. reported highly fluorescent CDs from China grass carp scales using a one-step hydrothermal method, which showed high sensitivity towards Hg^2+^ ions owing to the presence of sulfhydryl groups. The Hg^2+^ strongly coordinates with the sulfur atoms of the cysteine in the CDs, forming a hairpin structure S–Hg^2+^–S and, thus, quenching their fluorescence (Figure 10). A linear decrease in fluorescence intensity was observed with increased concentration of mercury ions with a LOD of 0.014 µmol/L. The low cytotoxicity and enhanced cell-permeability of the CDs make them sensible probes for Hg^2+^ detection in environmental and biological systems [142].

Highly stable, fluorescent, nitrogen-doped CDs obtained from citrus lemon juice were reported by Tadesse et al. The CDs were prepared via a hydrothermal method and showed high sensitivity and high selectivity towards Hg^2+^ ions with a LOD of 5.3 nM. The presence of oxygen- and nitrogen-containing groups on the surface of CDs facilitates coordination to Hg^2+^ by donating electron pairs to the metal ion, quenching the fluorescence of CDs. The quenching was ascribed to non-radiative electron–hole annihilation. Besides, human breast adenocarcinoma cells were tested for biocompatibility with these CDs. Very low cytotoxicity values were observed and, hence, the CDs could be used for bioimaging applications [143].

**Figure 11:**
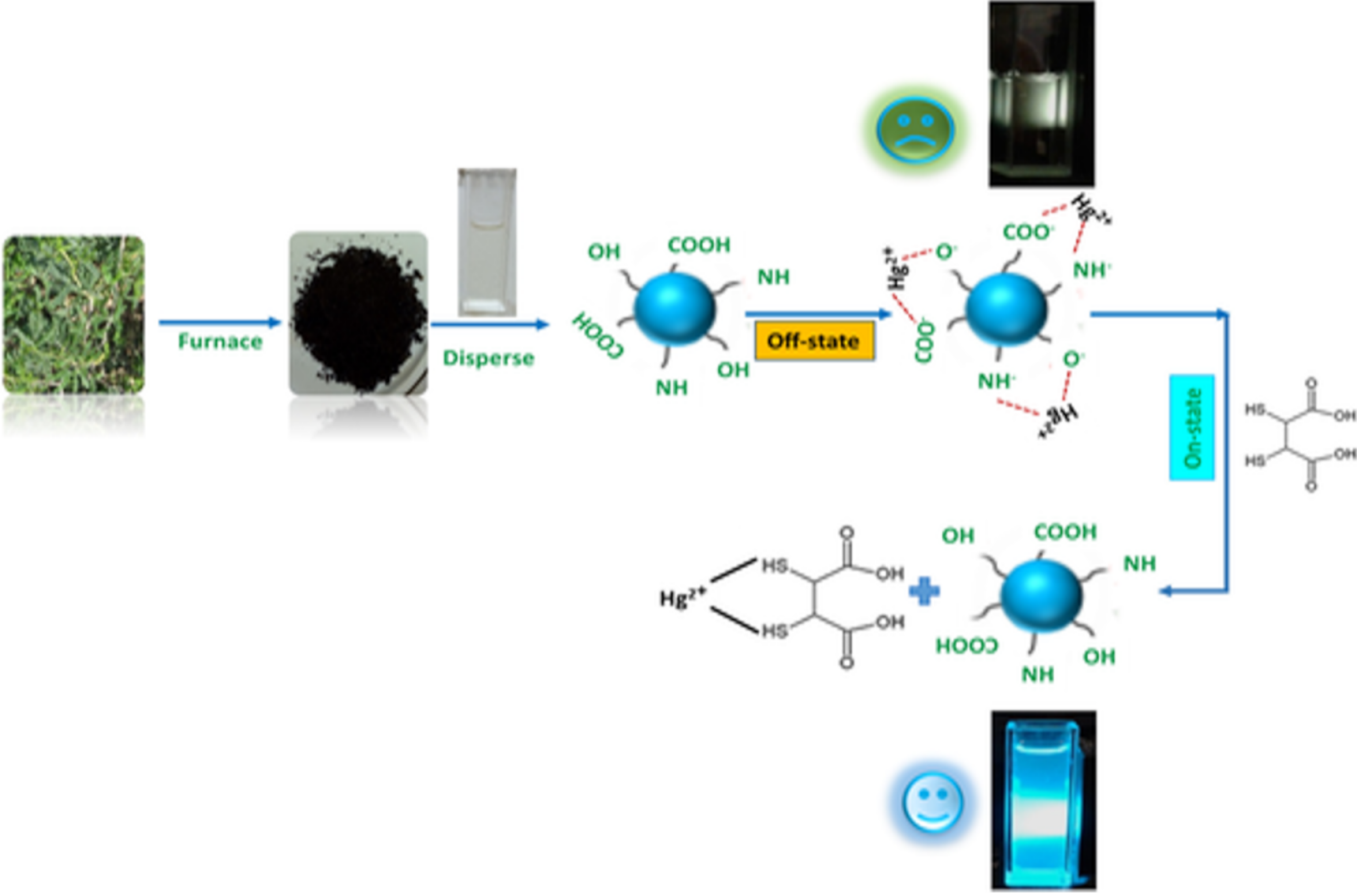
Schematic representation of off-on ﬂuorescence of CDs prepared from *Prosopis juliflora.* Figure 11 was reprinted from [23], Materials Science and Engineering: C, vol. 98, by N. Pourreza; M. Ghomi, “Green synthesized carbon quantum dots from *Prosopis juliflora* leaves as a dual off-on fluorescence probe for sensing mercury (II) and chemet drug”, pages 887-896, Copyright Elsevier (2019), with permission from Elsevier. This content is not subject to CC BY 4.0.

Pourreza et al. used *Prosopis juliﬂora* leaves to prepare CDs, which act as an off-on fluorescence sensor for Hg^2+^ ions and chemet detection, respectively. The addition of Hg^2+^ ions caused fluorescence quenching because of electrostatic interaction and non-radiative recombination of excitations via the transfer of electron between Hg^2+^ ions and oxygen-carrying groups present on the CD surface. When the Hg–CD system was treated with chemet, the fluorescence intensity of CDs was regained because of the strong interaction of Hg^2+^ ion with chemet (Figure 11) [23].

Li et al. reported the synthesis of N-CDs by using orange juice as a green source and EDA as a surface-passivating agent and used the CDs to detect Hg^2+^ ions. When the concentration of Hg^2+^ ions was increased, a decline in fluorescence intensity of N-CDs occurred and the full width at half maximum of the N-CDs emission peak slowly broadened because a complex formed between Hg^2+^ and carboxyl groups on the surface of the N-CDs [17].

Singh et al. used *Dunaliella salina* to synthesize N,P-CDs, which acted as a sensor for both Hg^2+^ and Cr^6+^ ions. The quenching through Hg^2+^ and Cr^6+^ occurred through dynamic quenching and IFE + dynamic quenching mechanisms, respectively [109].

Li et al. used Hongcaitai (*Brassica compestris L. var. purpurea Bailey***)** to prepare CDs that were categorized into two parts because of solubility differences. The one that was soluble in ethanol was named “CDs-A” and was used for ClO^−^ detection. The ethanol-insoluble CDs were named “CDs-B” and were used for Hg^2+^ detection. At pH 3, the strong oxidant HClO is mainly present, resulting in significant fluorescence quenching because of the oxidation of hydroxy groups present on the surface of the CDs. With the increase of pH, the concentration of ClO^−^, with lower oxidation strength as HClO, increased, which led to a decrease in fluorescence quenching. The fluorescence quenching by Hg^2+^ was assigned to its interaction with sulfur-containing functional groups on the surface of CDs-B, which resulted in non-radiative electron transfer [65].

Highland barley-derived N-CDs were produced via a hydrothermal process, where EDA was used for N-doping. The fluorescence intensity of the N-CDs diminished with increased concentration of Hg^2+^, which is likely due to a strong chelation of the metal ions by the –COOH groups on the surface of N-CDs causing static quenching [89].

Fluorescent N,S-CDs derived from gardenia fruit by Sun et al. via a hydrothermal method were highly stable in a wide pH range, high concentrations of sodium chloride, and under ultraviolet radiation. N,S-CDs exhibited on/off response towards cysteine and Hg^2+^. The fluorescence quenching was credited to the formation of Hg^2+^–S bonds, leading to a static quenching mechanism. Addition of cysteine resulted in a reversal of the quenching phenomenon because the interaction of the metal ions with sulfhydryl of the cysteine is stronger [53].

#### Cu^2+^ ion sensing

Copper is an essential trace element with different biological roles. However, high concentrations above a normal level of 100–150 μg/dL (15.7–23.6 μM) may cause toxicity causing Menkes and Wilsons diseases, gastrointestinal damage, and certain neurodegenerative diseases. Copper may enter the human body through drinking water polluted with Cu^2+^ ions above the permissible levels of 1.3 ppm. Many researchers have investigated CDs for efficient and easy-to-use Cu^2+^ sensing, suitable for use in aqueous and biological environments.

Polyolefin residues, resulting from pyrolytic degradation of waste plastic, are rich in carbon, and devoid of other heteroatoms, hence, a potential carbon source to be used for the preparation of CDs. Waste polyolefin residues, as a precursor, are advantageous in terms of reduced production cost. CDs as a probe for Cu^2+^ ions have been investigated. Kumari et al. obtained green fluorescent CDs from the pyrolytic residues of polyolefins via chemical oxidation without using any surface-passivating agents. The CDs exhibited green emission under UV light at 365 nm. These CDs exhibited high selectivity for Cu^2+^ ions in the presence of many other ions, under normal room temperature, with a detection limit of 1–8 µM/L. These CDs could also be used for a visual detection of Cu^2+^ ions, as a color change under UV light from a darker shade of green to a lighter one occurred upon addition of Cu^2+^ ions. Similarly, fluorescence quenching was detected with increased Cu^2+^ addition in tap water and mineral water samples, despite the presence of interfering minerals. The quenching was suggested to occur through a dynamic quenching mechanism, where Cu^2+^ ions coordinate to the carbonyl groups on the surface of the CDs [144]. These CDs also proved to be promising probes for breast cancer cell imaging (MDA-MB 468), owing to their significant cell permeability and reduced cytotoxicity.

Banana juice is rich in carbohydrates and, hence, a precursor for CDs. Chaudhary et al. reported on N,S-CDs prepared from banana juice via a hydrothermal method, exhibiting high fluorescence (blue emission) at pH 6. A decrease in fluorescence was observed below pH 6 and above 8. The N,S-CDs had high solubility in aqueous media with a wide range of detection for copper ions (1–800 µg/mL), observed in the form of fluorescence quenching. The N,S-CDs have a net negative charge and functional groups, such as hydroxy, carboxylic, and carbonyl groups, which assist in binding to Cu^2+^ ions, leading to formation of coordination bonds. This leads to a decrease in fluorescence activity, showing up as a redshift in the emission spectrum [162].

Biocompatible and highly stable blue CDs, derived from radish, were prepared through a hydrothermal method. The CDs were used for sensing of Cu^2+^ in water samples and vapors of acetic acid through an opto-electronic nose with CDs as sensing material. On addition of Cu^2+^ ions, fluorescence quenching was observed with a LOD of 0.16 µM. Owing to their high electron-donating ability, the –COOH groups on the CDs undergo complexation with Cu^2+^ ions, resulting in fluorescence quenching. A filter paper-based sensor incorporated with CDs was prepared with an improved detection limit of 6.8 µM. A measurement of the acetic acid concentration in a series of acetic acid/methanol mixtures was performed, with a limit of detection of around 15%. Vinegar samples were also tested accurately. A cytotoxicity assay of the CDs against MCF-7 breast cancer cells showed their biocompatibility with high cell viability, suggesting prospective applications in cell-imaging, bio-sensing, and drug delivery [145].

*Eleusine coracana*-derived green CDs were investigated as turn-off sensor for Cu^2+^. The strong affinity and selectivity of the CDs towards the metal ions were attributed to the presence of oxygen-rich functional groups, mainly –COOH, –NH, and –OH, on the surface of CDs. The Cu^2+^ ions are good electron acceptors and coordinate to the functional groups. Moreover, the paramagnetic Cu^2+^ ions adsorb in a relatively facile way on aromatic C=C bonds of the CDs, causing effective quenching [146].

Lily bulbs were used by Gu et al. to prepared N,P-CDs. The synthesized CDs were used to detect Cu^2+^ ions due to their dynamic affinity for different functional groups present on CDs surface, that is–COOH, –OH, and –NH_2_. A LB–CD complex is formed, which promises to boost charge shift and inhibits radiative recombination of excitons, resulting in a unique fluorescence quenching effect [108].

White pepper has been used as a green precursor for dual-emission CDs, which exhibited high selectivity towards coenzyme A. Emission at 520 nm (green) turned on the Cu^2+^-aided sensing, keeping the red emission at 668 nm as a reference. The fluorescence activity reduced slowly with an increase in the ion concentration from 0 to 65 µM. However, no significant decrease was observed when the concentration increased from 65 µM to 80 µM. When 65 µM Cu^2+^ were added to CDs, the absorption wavelength did not change, but intensity and fluorescence lifetime dropped. This indicated an interaction between the surface functional groups of the CDs and Cu^2+^. With the addition of CoA, fluorescence was restored, which was attributed to a strong conjugation between the phosphate and amino groups of CoA and Cu^2+^. The intensity of the fluorescence was observed to vary linearly with the concentration of coenzyme A [86].

**Figure 12:**
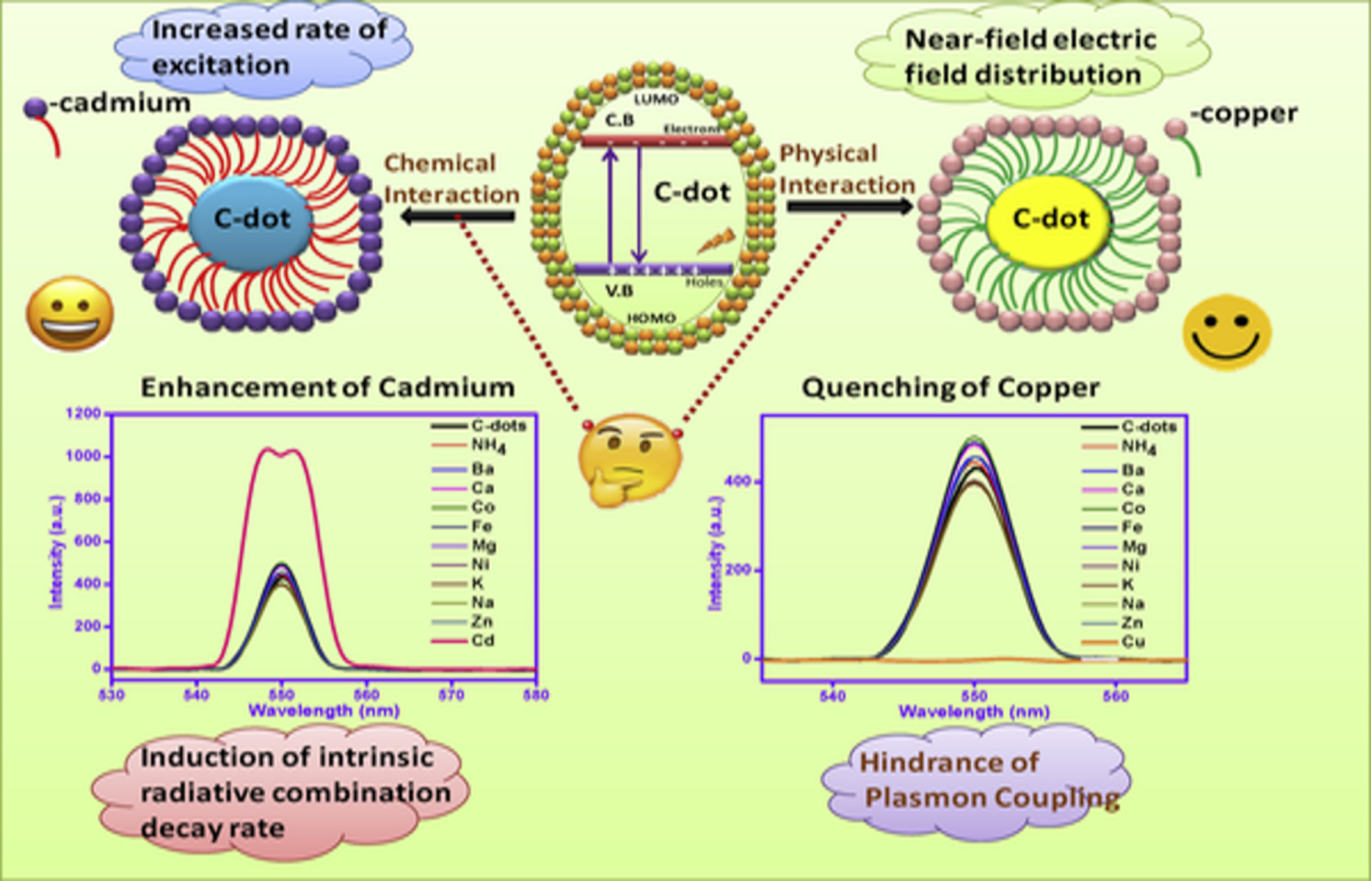
Schematic illustration of the sensing mechanism of CDs in the presence of Cd^2+^ and Cu^2+^ ions. Figure 12 was reprinted from [51], Materials Today Chemistry, vol. 16, by P. Chauhan; S. Dogra; S. Chaudhary; R. Kumar, “Usage of coconut coir for sustainable production of high-valued carbon dots with discriminatory sensing aptitude toward metal ions”, article no. 100247, Copyright Elsevier (2020), with permission from Elsevier. This content is not subject to CC BY 4.0.

Highly fluorescent CDs from coconut coir synthesized by a hydrothermal method demonstrated strong biocompatibility towards bacteria, various fungal strains, aquatic animals, and some plants when in low concentration. A sensor based on the synthesized CDs showed selectivity towards Cd^2+^ ions with a LOD of 0.18 nM (turn on) and Cu^2+^ ions (turn off) with a LOD of 0.28 nM in different media including sewage and groundwater systems. The fluorescence quenching in the presence of Cu^2+^ occurs due to a strong affinity of the metal ion for the hydroxy, carboxyl, and amino groups on the CDs, which leads to complex formation and non-radiative charge–hole annihilation. The emission intensity increased in the presence of Cd^2+^ ions, which was attributed to the induction of an intrinsic radiative recombination of the CDs (Figure 12). Furthermore, the chemical interaction between the surface of CDs and the Cd^2+^ ions also has an influence on the excitation of CDs [51].

#### Cd^2+^ ion sensing

Cadmium ions are highly poisonous heavy metal ions used for dying, electroplating, and in the semiconductor industries. The Cd^2+^ ions are toxic to animals and human beings and mainly affect liver, kidneys, lungs, and central nervous system. Cadmium poisoning manifests itself in the form of breath shortage, general weakness, pneumonia, and death in extreme cases. According to WHO, the permissible level of Cd^2+^ ions in drinking water is 3 ppb.

*Murraya koenigii*, commonly known as curry leaves were used for the synthesis of CDs by Pandey et al., which exhibited quenching with Cd^2+^ ions (LOD = 0.29 nM). The Cd^2+^ ions have vacant d orbitals, which bond with the –COOH and amino groups on the CDs through LMCT, leading fluorescence quenching. High selectivity for Cd^2+^ ions was exhibited even in the presence of other interfering metal ions such as Zn^2+^ [72].

Rice husk-based CDs prepared by Zainal Abidin et al. were functionalized with amino and carboxyl groups using EDA and ascorbic acid and denoted as N-CD and C-CDs, respectively. The PL of the C-CDs is reduced/quenched when an increased amount of ascorbic acid is added, which causes protonation of the CDs. Lowering of PL with increasing ascorbic acid concentration indicates reduced recombination of holes and electrons on the surface of the CDs. An increase in emission wavelength is observed, which is due to increased protonation of the negative CDs, leading to extensive electrostatic interactions among the CDs and agglomeration. A reverse trend was observed with the N-CDs and EDA. Both N-CDs and C-CDs exhibit a linear response to Cd^2+^, that is, a decrease in PL activity with increase in Cd^2+^ concentration. The quenching was thought to be due to electrostatic interactions between the metal cations and the negatively charged CDs, which are rich in oxygen-containing groups, leading to agglomeration. Moreover, electron transfer between the CDs and Cd^2+^ may result in reducing the latter to Cd. The quenching due to –COOH groups is stronger than that due to amino groups, because of high electronegativity of–COOH [147].

#### Pb^2+^ ion sensing

**Figure 13:**
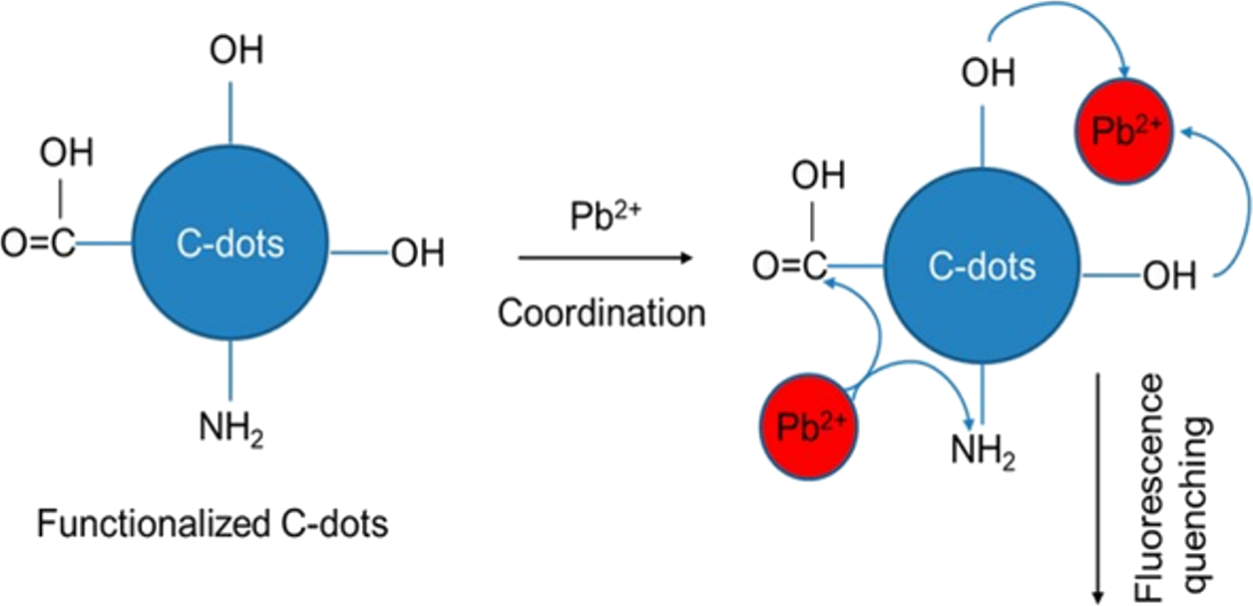
Schematic representation of Pb^2+^ ion sensing using oyster mushroom-derived CDs. Reprinted with permission from [157], Copyright 2020 American Chemical Society. This content is not subject to CC BY 4.0.

Lead is a heavy metal and is hazardous to human health. Lead poisoning causes serious damage to nervous system, kidneys, brains, and other vital organs. Pb^2+^ ions from water pipes containing lead enter into water when corrosion occurs due to acidic water. According to WHO, the upper limit of lead in drinking water is 0.05 mg·L^−1^. Boobalan et al. used oyster mushrooms (*Pleurotus* species) as a green precursor to prepare CDs for the detection of Pb^2+^ ions. They reported that quenching of CDs is because of the formation of a complex between Pb^2+^ ions and hydroxy and carboxyl groups present on the surface of the CDs (Figure 13) [157].

*Ocimum sanctum* (Tulsi leaves)-based fluorescent CDs were found to detect Pb^2+^ ions with excellent sensitivity and selectivity. The amine groups on the surface of CDs have great binding affinity with the vacant d orbital of the metal ion. Donation of an electron pair from the nitrogen of the functional groups to the empty d orbital of Pb^2+^ takes place and results in quenching of the fluorescence of CDs [163].

Table sugar-based CDs were reported, which acted as an efficient naked-eye sensor for lead ions. Small concentrations of Pb^2+^ ions, even at ppb levels turned the CD solutions turbid, which was explained by the formation of aggregates. Large aggregates scatter light and, hence, appear turbid. No other ions exhibited such behavior with these CDs [84].

#### Cr^6+^ ion sensing

Chromium has great importance in metallurgy, textile, dyes and leather tanning. However, it is highly toxic and carcinogenic. Two oxidation states, Cr(VI) and Cr(III) are more common and possess entirely different properties. The Cr(VI) compounds, due to their high solubility cause adverse effects in living organisms, whereas, the Cr(III) species are less toxic and important micronutrients. The permissible limit of the chromate(VI) (CrO_4_^2−^), according to WHO, is 0.05 mg·L^−1^.

Athika et al. synthesized CDs from denatured milk by a hydrothermal method, which demonstrated sensitivity towards Cr^6+^ with a detection limit of 14 mM in various water samples. Fluorescence quenching of the CDs was observed on adding Cr^6+^ mainly due to the inner filter effect. The CD electrode was found to exhibit a capacitance of 95 F·g^−1^ and stability over 1000 cycles [148]. *Hibiscus sabadariffa*-derived CDs prepared through a hydrothermal method were found to be suitable for bioimaging of breast cancer cells in addition to sensing Cr^6+^ ions [52].

CDs and N-CDs originated from groundnuts synthesized through a hydrothermal method showed remarkable selectivity towards certain metal ions. The CDs showed selective detection of Hg^2+^, Fe^3+^, and Cr^6+^, whereas the N-CDs sensed Cr^6+^ ions with a LOD of 0.1 mg/L. The fluorescence excitation/emission spectra showed that Cr^6+^ blocks the excitation wavelength of the N-CDs and also absorbs the emission intensity of CDs. Non-radiative electron–hole annihilation is also possible. The quenching mechanism in N-CDs was reversed with humic acid and glutathione. Humic acid and glutathione reduce Cr^6+^ to Cr^3+^, which reverses quenching as the N-CDs are not reactive towards Cr^3+^. The CDs were biocompatible with low cytotoxicity values, making them suitable for bioimaging of MCF-7 cells [149].

**Figure 14:**
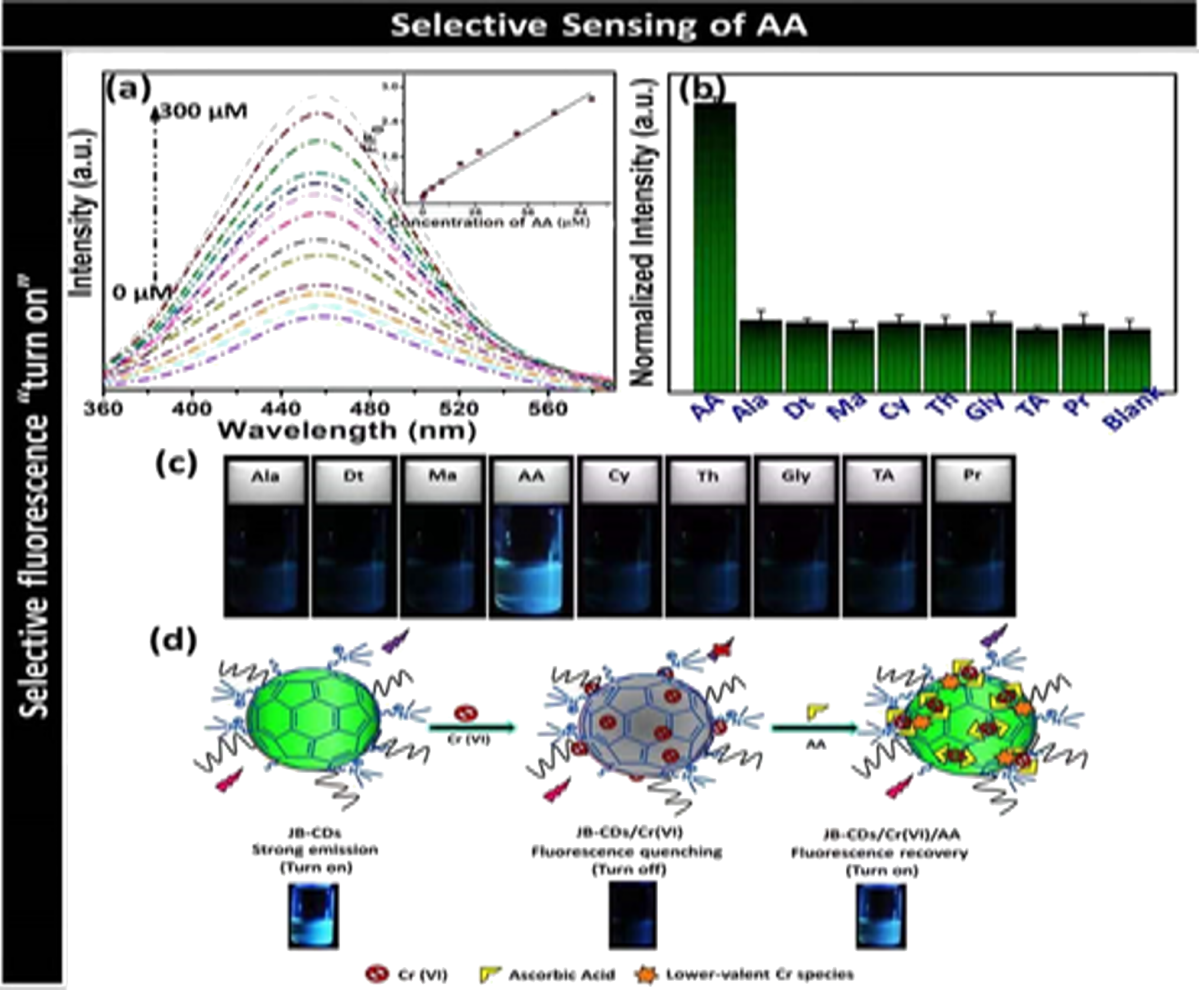
Fluorescence quenching and restoration of jute caddies based modified CDs, in the presence of Cr^6+^ and AA. Reprinted with permission from [150], Copyright 2020 American Chemical Society. This content is not subject to CC BY 4.0.

Das et al. proposed a sustainable way to use jute caddies to synthesize CDs by a sonochemical approach. The CDs modified with benzalkonium chloride were reported to detect Cr^6+^ by luminescence quenching and selectively restore its fluorescence to detect ascorbic acid (Figure 14). The IFE was proposed to be a possible quenching mechanism because a significant spectral overlap between the absorption band of Cr^6+^ and the excitation and emission spectra of the CDs occurs when AA was introduced into the CD/Cr^6+^ system. AA reduces Cr^6+^ to Cr^3+^ or some lower oxidation state, which helped to eliminate the IFE and to restore the fluorescence intensity. Thus, a fluorescence turn-off/on sensor was developed [150].

Luo et al. used luffa sponge to synthesize CDs by a chemical oxidation approach. The fluorescence quenching effect of Cr^6+^ on the CDs was studied. The results showed that the addition of Cr^6+^ changed the intensity of the characteristic absorption peaks of CDs and led to the static quenching of the fluorescence. The IFE was used to explain the fluorescence quenching. The Cr^6+^ ions show absorption at 260, 360, and 440 nm. The excitation spectra of CDs exhibit prominent excitation and emission bands at 360 and 473 nm, respectively. There is an obvious overlap in the 360 nm excitation spectra, which indicates that Cr^6+^ can shield the excitation light of CD. Therefore, an increase in Cr^6+^ concentration may result in a stronger fluorescence quenching of CD [151]. The same mechanism was also proposed by Shreya Bhatt et al. to detect Cr^6+^. They used tulsi leaves to synthesize CDs and reported a LOD and linear range of 4.5 ppb and 1.6 to 50 μM, respectively [152].

**Figure 15:**
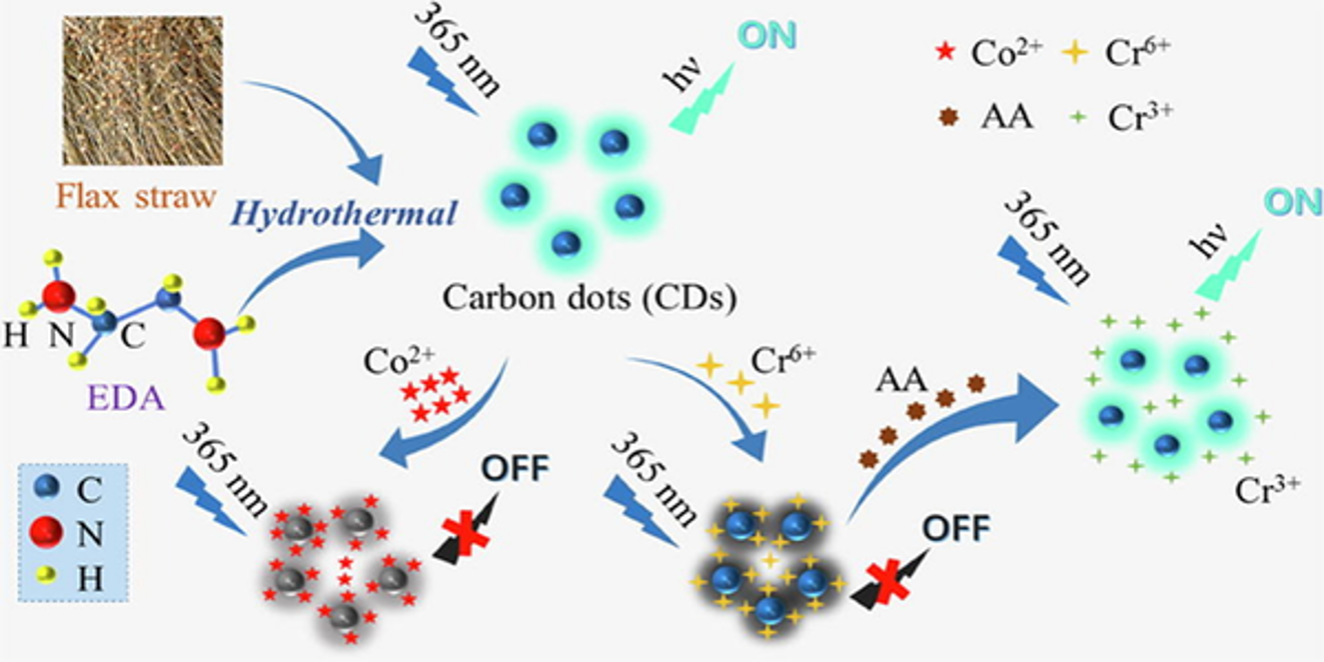
Schematic illustration of the CD synthesis from flax straw and the detection of Co^2+^, Cr^6+^, and AA. Figure 15 was reprinted from [87], Journal of Colloid and Interface Science, vol. 579, by G. Hu; L. Ge; Y. Li; M. Mukhtar; B. Shen; D. Yang; J. Li, “Carbon dots derived from flax straw for highly sensitive and selective detections of cobalt, chromium, and ascorbic acid”, pages 96-108, Copyright Elsevier (2020), with permission from Elsevier. This content is not subject to CC BY 4.0.

Recently, Hu used flax straw as a green source to synthesize CDs by a hydrothermal method. The reported CDs possess “on-off” fluorescence behavior in the presence of Co^2+^ or Cr^6+^ ions, which is further protracted to “on-off-on” behavior for ascorbic acid detection (Figure 15). The “on-off” fluorescence behavior is based on static quenching and the IFE and “on-off-on” fluorescence behavior occurs because ascorbic acid can reduce Cr^6+^ to Cr^3+^ due to which the IFE weakens and fluorescence of CDs recovers [87].

CDs prepared from dried rice fried *Codonopsis pilosula* by Qiu et al. were found to be highly selective and sensitive towards Cr^6+^ in water bodies and industrial affluents. The absorption band of Cr^6+^ overlapped with the excitation band of the CDs pointing to an IFE process [74].

**Figure 16:**
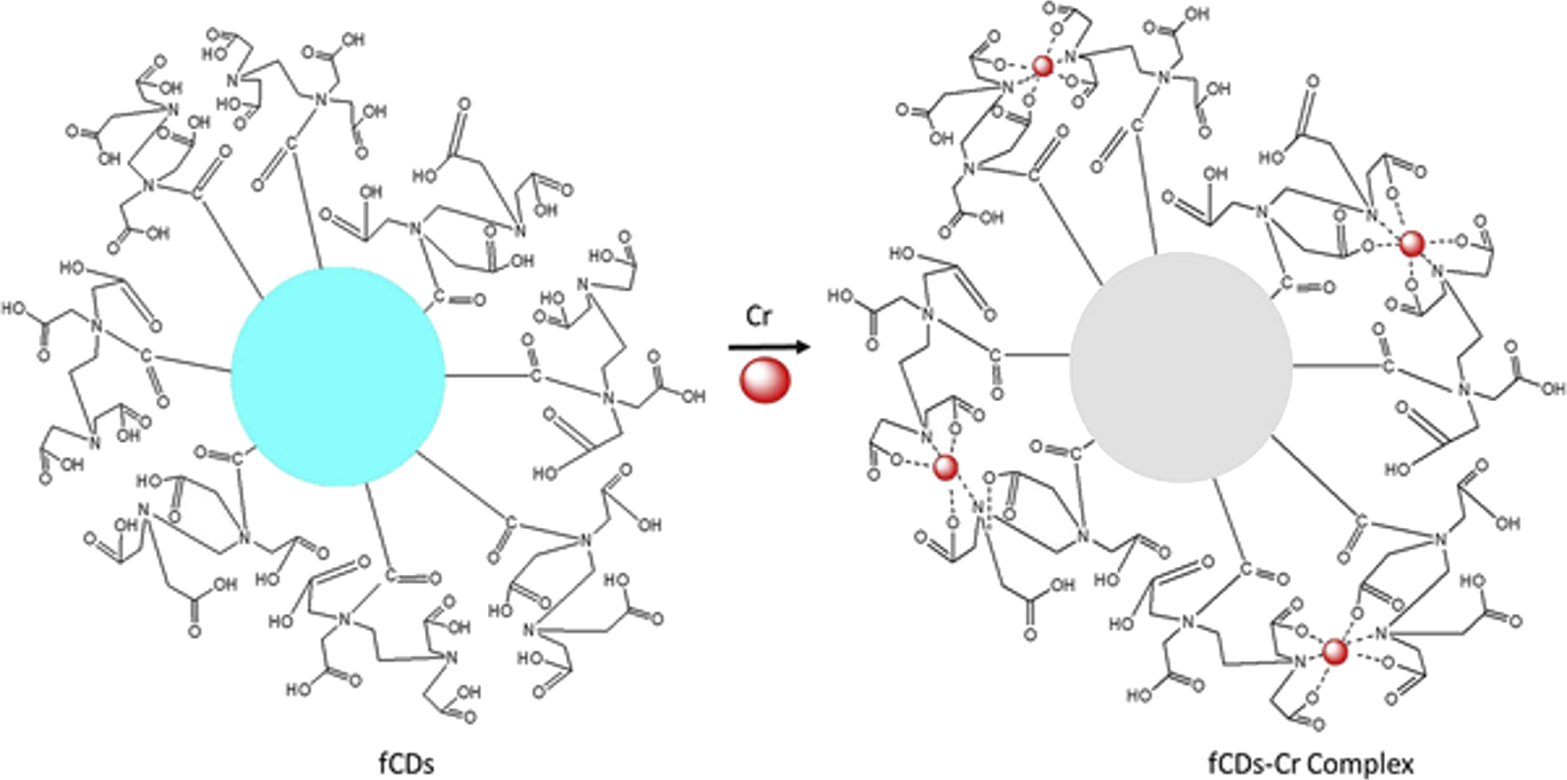
Proposed mechanism of chromium sensing with f-CDs. Figure 16 was reprinted from [83], Sensors and Actuators B: Chemical, vol. 283, by P. D.; L. Singh; A. Thakur; P. Kumar, “Green synthesis of glowing carbon dots from *Carica papaya* waste pulp and their application as a label-freechemo probe for chromium detection in water”, pages 363-372, Copyright Elsevier (2019), with permission from Elsevier. This content is not subject to CC BY 4.0.

CDs synthesized from *Carica papaya* waste via pyrolysis were utilized for detecting the content of Cr^6+^ and Cr^3+^ in water. Functional groups, such as carbonyl and carboxylic groups, on the CDs help in their modification with EDTA, producing EDTA-functionalized CDs (fCDs). The fCDs, then coordinate with the chromium ions through oxygen and nitrogen of the functionalized ethylene diamine (Figure 16) [83].

#### Co^2+^ ion sensing

Cobalt is an essential trace metal, which controls the production of red blood cells and also regulates many other vital processes involving the catalytic activity of some important enzymes. Besides, it is an important component of vitamin B_12_. Excessive levels of cobalt give rise to low blood pressure, asthma, dermatitis, and myocardial infarction. The permissible levels of cobalt in water for irrigation and livestock are 0.05 and 1 mg·L^−1^, respectively.

Kelp-based CDs with ethylenediamine as nitrogen dopant prepared via microwave irradiation were found to be highly pH sensitive. High pH values resulted in decreased fluorescence, with a linear pattern in the range of 3–8. They also showed selectivity and sensitivity towards Co^2+^ ions (LOD = 0.39 µmol·L^−1^) with an immediate color change from transparent to yellowish-brown. The fluorescence was found to be quenched linearly with increasing concentration of Co^2+^ ions. A good spectral overlap of the absorption spectra of the metal ions and emission spectra of the CDs suggested IFE or FRET to be in action. However, the fluorescence lifetime decay data was the same with and without metal ions, which suggested that the FRET mechanism was not possible. Thus, IFE was the predicted mechanism for the quenching process [153].

#### Au^3+^ ion sensing

Gold is a widely used noble metal in catalysis and medicine. However, it induces harmful effects in the human body and other biological systems owing to its strong affinity and reactivity towards DNA and some enzymes. The adverse effects include deterioration of the peripheral nervous system, nephrotoxicity, and liver damage [114–115].

Arumugam et al. reported CDs synthesized from denatured sour milk and used them to detect gold ions by simply mixing the aqueous dispersion of CDs with ascorbic acid (AA). The addition of Au^3+^ did not disturb the fluorescence intensity of CD, which ruled out the possibility of reduced electron transfer or metal ion-induced aggregation. In the current case, the CD/AA system fluorescence quenching can be attributed to the AA-mediated reduction of Au^3+^ to AuNPs and ensuing IFE or FRET. The LOD obtained by the said system was approximately 0.95 μM [19].

**Figure 17:**
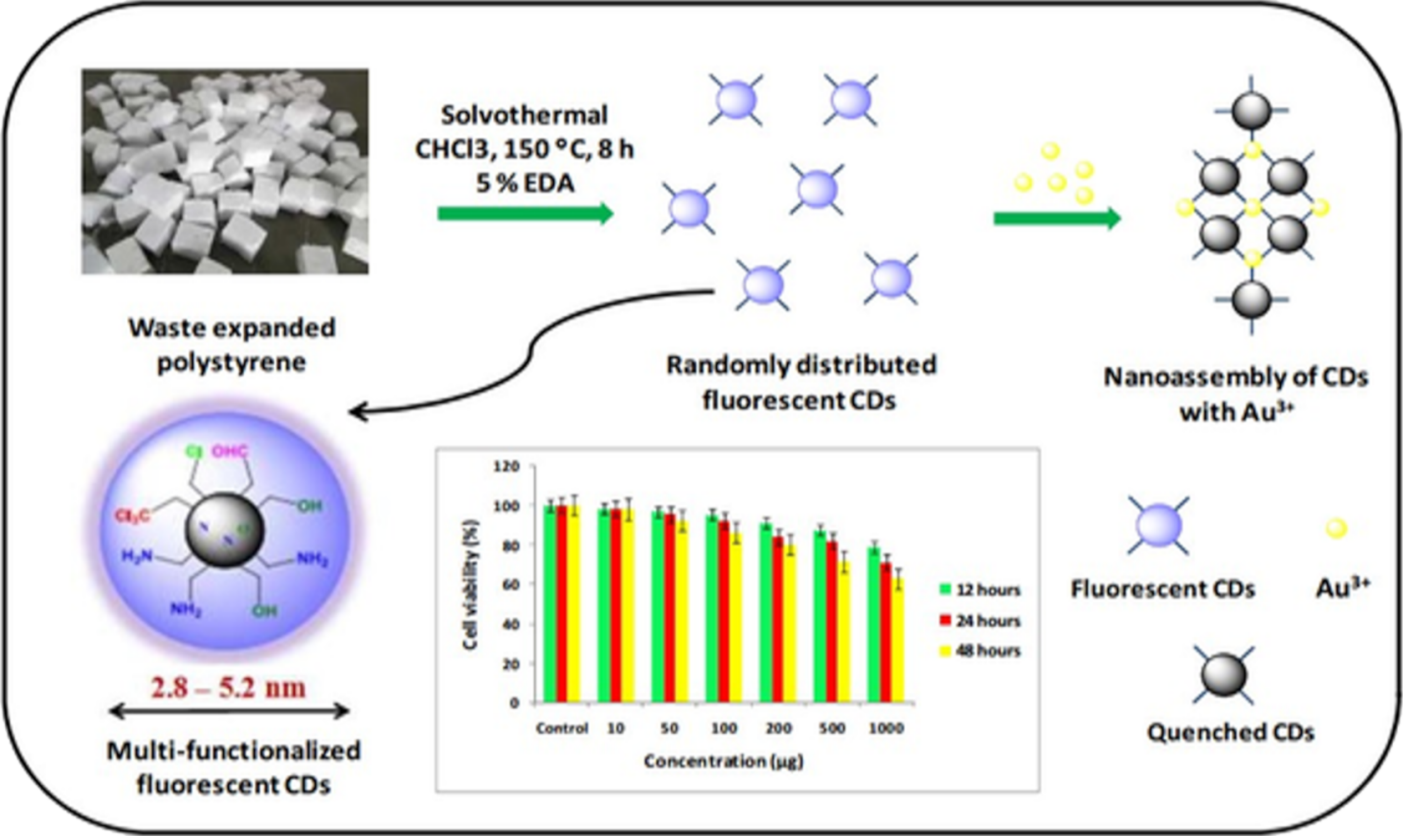
Highly luminescent, multi-functionalized, cell-viable CDs were prepared from waste expanded polystyrene and demonstrated as an efficient probe for Au^3+^ sensing. Reprinted with permission from [154], Copyright 2020 American Chemical Society. This content is not subject to CC BY 4.0.

Ramanan et al. also designed a sensor for Au^3+^ from waste expanded polystyrene (EPS). A detailed exploration suggests that PL quenching is because of “coordination-induced aggregation” (Figure 17). The LOD and linear range obtained were 53 nM and 0 to 35 µM, respectively [154].

#### As^3+^ ion sensing

Arsenic toxicity manifests itself by disrupting cellular energy pathways, where arsenic deactivates enzymes. It also adversely affects DNA synthesis and repair. Arsenic poisoning is associated with nausea, vomiting, and diarrhea. The allowed limit of Ar^3+^ in drinking water is 10 µg·L^−1^, as recommended by WHO.

**Figure 18:**
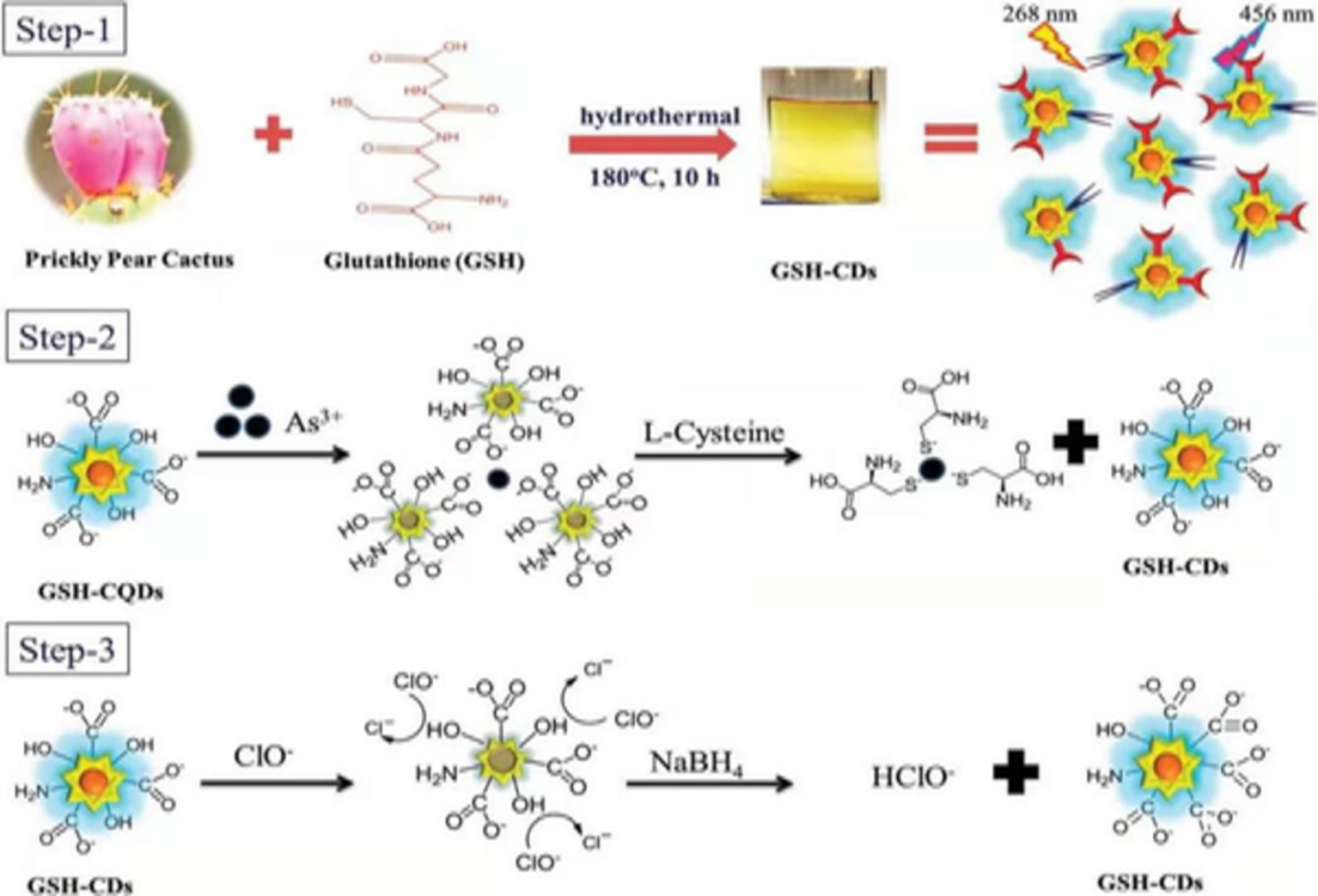
Schematic illustration of the design and working principle of prickly pear cactus-based CDs. Reproduced from [156] (“Green synthesis of surface-passivated carbon dots from the prickly pear cactus as a fluorescent probe for the dual detection of arsenic(iii) and hypochlorite ions from drinking water “, © 2018 K. Radhakrishnan et al., published by The Royal Society of Chemistry, distributed under the terms of the Creative Commons Attribution 3.0 Unported License, https://creativecommons.org/licenses/by/3.0/).

Prickly pear cactus-based surface-passivated CDs were reported by Radhakrishnan et al. using a hydrothermal method. The synthesized CDs exhibited a turn-off response to As^3+^ and ClO^−^. Glutathione was used a surface-passivating agent that contains various functional groups. The resulting CDs have a negative charge due to the presence of –COOH, –C=O, and –OH groups and, hence, efficiently chelate the As^3+^ ions, quenching fluorescence through IFE (Figure 18) [156].

Ramezani et al. synthesized CDs to detect As^3+^ by using quince fruit (*Cydonia oblonga*) as a green source. Determination of As^3+^ is based on its oxidation by MnO^4−^. Oxidants, such as KMnO_4_, are reported to generate holes in CD. This process rises the number of holes in the CDs and increases electron–hole annihilation. As a result, the energy release in the form of chemiluminescence (CL) emission and fluorescence quenching at the maximum emission wavelength of CDs was obtained. Adding As^3+^ to MnO^4−^ before mixing with CD increases photoluminescence compared to solely permanganate. This increase was linear with As^3+^ concentration in the range of 0.1 to 2 μg·L^−1^ [155].

#### Ag+ ion sensing

*Cryptococcus podzolicus*-based blue fluorescent CDs exhibited remarkable selectivity and sensitivity towards Ag^+^, 2,4-dinitrophenol, and 4-nitrophenol. It was established that a static quenching mechanism dominated when Ag^+^ was added to the fluorescent CDs, whereas IFE dominated with 2,4-dinitrophenol and 4-nitrophenol. The absorption spectra of the CDs were different with and without Ag^+^, indicating the formation of a new compound. Hence, static quenching could be predicted. No change was detected in the absorption spectra of CDs with or without 2,4-dinitrophenol and 4-nitrophenol, and, hence, no SQ could occur. The absorption bands of the nitrophenols overlapping with the excitation spectra condoned the inference that IFE might be the possible quenching mechanism [158]. Table 6 shows the color of various green source-derived CDs, their fluorescence under UV light, LOD, linear range, and sensing strategies for different metal ions.

**Table 6:** Green CDs used for sensing metal ions, against their limit of detection, linear ranges, and sensing strategy.

No.	Source	Color under UV light	Sensing	Source of analyte	LOD	Linear range	Sensing strategy	Ref

1	*Boswellia ovalifoliolata*	blue	Fe^3+^	simulated water sample	0.41 µM	up to 500 μM	ﬂuorometry	[138]
2	*Citrus limetta*	blue	Fe^3+^	simulated water sample	19.8 ppb	50–100 ppb	fluorometry	[139]
3	betel leaves	blue	Fe^3+^	simulated water sample	50 nM	50–150 nM	fluorometry	[140]
4	*Miscanthus grass*	blue	Fe^3+^	simulated water sample	20 nM	0.02 to 2000 mM	fluorometry	[141]
5	*Ananas comosus*	blue/green/ yellow	Fe^3+^	urine, drugs, and plasma	0.03 μM	0.05–500 μM	ﬂuorometry	[85]
6	*Bauhinia flower*	blue	Fe^3+^/ATP	tap water and human serum	0.01 μM/ 0.005 μM	10–350 μM/ 0.01 to 450 μM	ﬂuorometry	[104]
7	*Prunus cerasifera*	blue	Fe^3+^	simulated water sample	—	0–0.5 mM	ﬂuorometry	[49]
8	*Borassus flabellifer*	blue	Fe^3+^	simulated water sample	10 nM	0 to 30 nM	ﬂuorometry	[59]
9	watermelon juice	blue	Fe^3+^/cys	simulated water sample	0.16 μM/ 0.27 μM	0–300 μM/ 0–250 μM	ﬂuorometry	[16]
10	grape and onion	–	Fe^3+^	tap water and river water	0.1 mM	4.6–160 mM	colorimetry	[159]
11	starch	green	Fe^3+^	waste water treatment plant, lake water and tap water	3.2 nM	0.01 to 16 µM	ﬂuorometry	[160]
12	*Aloe vera*	blue	Fe^3+^	simulated water sample	33 ppb	70 ppb–10 ppm	ﬂuorometry	[67]
13	*Magnolia liliiflora*	blue	Fe^3+^	simulated water sample	1.2 μM	1–1000 μM	ﬂuorometry	[103]
14	*Catharanthus roseus*	green	Fe^3+^	simulated water sample	—	0 to 0.4 μg·L^−1^	ﬂuorometry	[161]
15	*Lonicera maackii*	green	Fe^3+^	tap water and urine samples	0.08 μM	0.1–10 μM	fluorometry	[107]
16	crop biomass	blue	Fe^3+^	—	—	0–500 μM	fluorometry	[77]
17	cornstalk	blue	Fe^2+^ and H_2_O_2_	simulated water sample	0.18 μM and 0.21 μM	0 to 18.0 μM (Fe^3+^)	ﬂuorometry	[25]
18	china grass carp scales	blue	Hg^2+^	lake water	0.014 μM	0.014–30 μM	ﬂuorometry	[142]
19	citrus lemon juice	blue	Hg^2+^	tap water and packed water sample	5.3 nM	0.01 to 0.05 mM	ﬂuorometry	[143]
20	*Prosopis juliﬂora*	blue	Hg^2+^/chemet	river, waste water samples from saponification company and petrochemical company, and serum samples	1.26 ng·mL^−1^/ 1.4 ng·mL^−1^	5–500 ng·mL^−1^ and 2.5–22.5 ng·mL^−1^	ﬂuorometry	[23]
21	orange juice	blue	Hg^2+^	tap water	—	0.0–32.0 μM	ﬂuorometry	[17]
22	*Dunaliella salina*	blue	Hg^2+^/Cr^2+^	distilled water	0.018 μM	0.03–0.20 μM/ 0.03–0.18 μM)	ﬂuorometry	[109]
23	*Hongcaitai*	blue	Hg^2+^/ClO^−^	tap water, river water	0.06 μM/ 0.015 μM	0.2–15 μM/ 0.05–15 μM	ﬂuorometry	[65]
24	highland barley	blue	Hg^2+^	simulated water sample	0.48 µM	10–160 µM	ﬂuorometry	[89]
25	*Gardenia fruit*	blue	Hg^2+^/Cys	simulated water sample	320 nM/ 271 nM	2–20 μM/ 0.1–2.0 μM	ﬂuorometry	[53]
26	polyolefin residues	green	Cu^2+^	bottled mineral water and tap water	6.33 nM	1–8.0 M	fluorometry	[144]
26	banana juice	blue	Cu^2+^	simulated water sample	0.3 μg·mL^−1^	1–800 μg·mL^−1^	fluorometry	[162]
27	radish	blue	Cu^2+^, acetic acid vapors	tap water and river water	0.16 μM/ 15.5%	—	fluorometry, optical electronic nose	[145]
28	*Eleusine coracana*	blue	Cu^2+^	tap and river water	10 nM	0 to 100 μM	ﬂuorometry	[146]
29	lily bulbs	blue	Cu^2+^	tap water and lake water	12.8 nM	0.05–2.0 μM	ﬂuorometry	[108]
30	white pepper	green	coenzyme A, Cu^2+^	pig liver samples	8.75 nM	0–150 µM	ﬂuorometry	[86]
31	coconut coir	blue	Cu^2+^, Cd^2+^	deionized water, tap water, sewage water, and ground water	0.28 nM/ 0.18 nM	0.28–0.93 nM/ 0.18–0.61 nM	fluorometry	[51]
32	*Murraya koenigii*	blue	Cd^2+^	tap water and pond water	0.29 nM	0.01–8 μM	ﬂuorometry	[72]
33	rice husks	green	Cd^2+^	simulated water sample	—	—	ﬂuorometry	[147]
34	oyster mushroom (*Pleurotus species*)	green	Pb^2+^	simulated water sample	58.63 μM	10–200 μM	colorimetry	[157]
35	*Ocimum sanctum*	green	Pb^2+^	pond water, tap water and mineral water	0.59 nM	0.01–1.0 M	ﬂuorometry	[163]
36	table sugar	blue	Pb^2+^	tap water, well water and lake	67 nM	—	turbidimetry	[84]
37	denatured milk	blue	Cr^6+^	drinking water	14 µM	—	fluorometry	[148]
38	*Hibiscus sabadariffa*	blue	Cr^6+^	simulated water sample	—	—	fluorometry	[52]
39	*Groundnuts*	—	Cr^6+^	simulated water sample	0.1 ppm (1.9 μM)	—	ﬂuorometry	[149]
40	jute caddies	blue	Cr^6+^/AA	tap water	0.03 μM	1–140 μM	ﬂuorometry	[150]
41	luffa sponge	green	Cr^6+^	tap water	—	—	ﬂuorometry	[151]
42	tulsi leaves	blue	Cr^6+^	tap water	4.5 ppb	1.6 to 50 μM	ﬂuorometry	[152]
43	flax straw	blue	Co^2+^/Cr^6+^/ AA	tap water, river water	0.38/0.19/ 0.35 μM	0–500/0.5–80/ 0–200 μM	ﬂuorometry	[87]
44	rice fried *Codonopsis pilosula* (CP)	blue	Cr^6+^	river, tap and lake water	15 nM	0.03–50 μM	ﬂuorometry	[74]
45	*Carica papaya*	blue	Cr^3+^, Cr^6+^	ground, tap and well water	0.708 ppb	10–1000 ppb	ﬂuorometry	[83]
46	kelp	blue	Co^2+^	river water	0.39 μM	1–200 μM	fluorometry	[153]
47	denatured sour milk	blue	Au^3+^	drinking water	0.95 μM	10–150 μM	ﬂuorometry	[19]
48	waste expanded polystyrene	blue	Au^3+^	mineral water, tap water, pond, lake and sea water	53 nM	0 to 35 µM	ﬂuorometry	[154]
49	prickly pear cactus	blue	As^3+^/ClO^−^	pond, river and industrial waste water	2.3 nM/ 0.016 μM	2–25 nM/ 10–200 μM	fluorometry	[156]
50	quince fruit (*Cydoniaoblon*)	blue	As^3+^	tap water	0.04 μg·mL^−1^	0.1 to 2 μg·L^−1^	ﬂuorometry	[155]
51	*Cryptococcus Podzolicus*	blue	Ag^+^/ (2,4-DNP)/ (4-NP)	tap water, river water and waste water	113.57 nM/ 73.36 nM/ 86.47 nM	0–15 μM/ 0–30 μM/ 0–37.5 μM	ﬂuorometry	[158]

### Applications in bioimaging, detection, sensing, and viability studies

Agriculture is a backbone of various industries and aimed to feed people and animals. Plant pathogens result in huge economic losses by deteriorating the quality and quantity of the produce. To devise the best disease management strategies under greenhouse and open field conditions, accurate and rapid identification of plant pathogens is the first important step. Traditional disease diagnostic methods and a few latest technologies, such as quantitative polymerase chain reaction (Q-PCR), are expensive, time-consuming, and lack high sensitivity. In recent years, engineered nanoparticles are getting more attention due to their wide application spectra [164]. CDs, due to their smaller size, non-toxicity, biocompatibility, water-solubility [165], synthesized from organic waste matter are extensively applied in different sectors such as wastewater sensors, switches, agro-fertilizers [166], as bactericides, fungicides, and nanoscale fertilizers [167]. Anand et al. published a mini-review in which they highlighted the utilization and potential of CDs in biosensing, nanomedicine, photo-catalysis, bioimaging, and as antimicrobial agents [168].

Recent reports on the utilization of CDs in bioimaging [26,123], sensing [57,106], and catalysis [27,96] have gained the attention of the scientific community. The use of fluorescence techniques in imagining microorganisms has gained much popularity to study the structure and state of microorganisms. This study is helpful for microbial quantification, viability testing, and Gram-type identification of microorganisms [43]. Various recent reports are available on the successful utilization of green CDs in bioimaging of bacterial strains such as *Escherichia coli*, *Staphylococcus aureus* [169], *Pseudomonas aeruginosa* and fungal cells of *Fusarium avenaceum* [170], *Bacillus subtilis* and fungus *Aspergillus aculeatus* [171], and other fungal agents such as *Fomitopsis* spp. [48] and yeast cells [94].

Kasibabu et al. [170] prepared fluorescent CDs from pomegranate fruits and successfully used them in bioimaging of *P. aeruginosa* and *F. avenaceum*. In a similar study, Atchudan et al. [94] synthesized N-CDs from *Chionanthus retusus* fruit extract and used them as a biological probe to study *Candida albicans* and *Cryptococcus neoformans* under a fluorescence microscope. Wang et al. [15] synthesized CDs from papaya and used them as a probe for fluorescence sensing of *Escherichia coli* bacteria. Amphiphilic CDs are used for microbial monitoring. A research study [172] successfully manipulated these CDs for the detection of bacteria.

Microbial viability testing is carried out in microbial monitoring, detection, and antibiotic development. Usually, the plate count method is employed, which is laborious and time-consuming. Newly developed technologies such as surface-enhanced Raman scattering [173] and Fourier-transform infrared spectroscopy [174] are more accurate than conventional plate counting. However, they are costly, time-consuming, and require more expertise. In contrast, the use of CDs as probes for microbial viability testing has opened new research dimensions. Bacteria-derived and yeast extract-based CDs probes have been successfully employed in microbial viability testing, which has broadened their applications in bio-medical sciences [141–142].

Gram staining is a very basic technique used to identify the bacterial types Gram-positive and Gram-negative. This is important to choose which type of antibiotics can be used to treat a bacterial infection. The traditional Gram staining procedure is laborious and can also produce false-positive results [175–176]. The use of fluorescent CDs in Gram-type identification is more accurate and rapid. In a study, vancomycin-conjugated CDs were employed to identify Gram-positive *S. aureus* and many other bacteria [177].

Atchudan et al. [94] utilized the hydrothermal carbonization technique to prepare N-CDs from the fruit extract of *Chionanthus retusus*. The N-CDs showed strong fluorescence properties and low cytotoxicity and were used in metal ion detection. The prepared N-CDs, due to their water solubility, cell permeability, high fluorescence, and low cytotoxicity, were employed in various biomedical applications. Furthermore, these N-CDs were used as biological probes to obtain fluorescent microscopy images of yeast infection in living samples. Bhamore et al. [48] obtained multicolor emitting CDs from *Manilkara zapota* fruits. Due to no cytotoxicity and strong biocompatibility, these CDs were effectively employed in the bioimaging of various bacterial and fungal cells.

In a similar study, Pal et al. [92] prepared surface-passivated CDs (CDPs) from the curcumin plant. Due to their smaller cytotoxicity, these CDP were successfully used in the biolabeling of cancer cells. The prepared CDPs were also applied in the bioimaging of Zebrafish embryos. Fluorescent N-CDs were prepared from the extracts of *Hylocereus undatus* and were characterized by using different methods [93]. The prepared N-CDs displayed less cytotoxicity and good biocompatibility on L-929 and MCF-7 cells and showed promising catalytic potential towards the reduction of methylene blue. Luminous CDs displaying less cytotoxicity and strong biocompatibility were prepared from apple juice and were successfully employed in the bioimaging of various bacterial and fungal cells [178]. In another study of Zhang et al. [116], urine-based CDs (UCDs) and hydrothermally treated urine CDs (HUCDs) were synthesized by utilizing a simple sephadex filtration procedure and a hydrothermal reaction approach. The prepared UCDs and HUCDs were used for in vivo and in vitro bioimaging of cells and displayed good biocompatibility and no toxicity to normal rat kidney cells.

CDs have become an attractive materials class for killing pathogenic microbes, including bacteria [179–181], and viruses [182]. Positively charged CDs interact with the negatively charged surface of bacteria, resulting in bacterial cell death by damaging the bacterial cell surface. In addition to these CDs, antimicrobial cationic CDs and negatively charged CDs also showed antibacterial potential [144]. Fungus-derived photosensitizer-conjugated CDs such as MCDs were successfully employed to kill bacterial agents [138].

Despite great potential, only a few reports are available on the extensive application of nanoscale carbon materials in plant disease control. In a research study by Han et al. [28], cow milk-derived CDs were synthesized, which showed promising antibacterial efficacy against *Staphylococcus aureus* and *E. coli* bacteria. Surendran et al. [101] prepared fluorescent CDs from natural honey by using a hydrothermal method. The spherical shape of the CDs was determined by XRD and HRTEM, while the presence of nitrogen and sulfur atoms was confirmed using FTIR and XPS analysis. For the first time, self-defocusing nonlinearity and strong nonlinear characteristics were identified by using the Z-scan. These CDs showed antimicrobial potential against foodborne pathogens in vitro. Similarly, CdS CDs were prepared from the leaf extract of *Camellia sinensis* and found to possess strong antibacterial potential against different bacterial strains [183]. These CdS-CDs also showed cytotoxicity against A549 cancer cells and the results were comparable to those of the standard drug cisplatin. These CDs inhibited the cancer cell growth by encountering with the cells during the S phase of the cell cycle and they also produced high-contrast fluorescence images of A549 cancer cells.

Recently, Das et al. [14] utilized jute industry waste to prepare fluorescent surface-quaternized CDs (JB-CDs) with high water solubility and photostability. These were successfully used in the biosensing of chromium ions in water. Furthermore, JB-CDs displayed significant growth inhibition of *E. coli* and *S. aureus*. These JB-CDs also exhibited a pH-responsive release of ciprofloxacin at pH 7.4. In another study of Boobalan et al. [157], fluorescent blue/green CDs were synthesized from *Pleurotus* species and were reported to display the potential of metal ion detection. These CDs showed promising applications as a fluorescent probe for DNA detection and showed effective antibacterial potential against *S. aureus*, *K. pneumoniae*, and *P. aeruginosa*. These CDs also displayed anticancer potential against breast cancer cells.

In a similar study, CDs were prepared from *Lawsonia inermis* plant by employing a low-cost hydrothermal method and these were found to be effective antibacterial agents against various Gram-positive and Gram-negative bacterial strains [184]. In another study of Yallappa et al. [185], nanoscale carbons (NCs) with strong fluorescent potential and biocompatibility were prepared from the waste groundnut shells. The biocidal potential of these NCs was tested against *Escherichia coli*, *Chromobacterium violaceum,* and *Bacillus cereus.* The NCs were found to possess strong antibacterial potential. Another study highlighted the promising bioimaging and antibacterial potential of fluorescent CDs [186]. CDs were prepared from sugarcane bagasse and were characterized by employing various techniques. The findings showed excellent antibacterial efficacy of the CDs against numerous Gram-positive and Gram-negative bacterial strains.

Das et al. [14] prepared CDs (KLB-CDs) from seaweed-derived κ-carrageenan and lemon juice, which showed promising water solubility, upconversion photoluminescence, and photostability. These KLB-CDs were successfully used as a biosensor for the detection of chromium and showed antibacterial potential against *E. coli*. Devi et al. [67] employed a one-step pyrolysis approach for the preparation of CDs from *Aloe vera*. The CDs displayed excellent biosensing and antibacterial potential. CDs derived from *Artemisia argyi* leaves showed selective antibacterial efficacy [187]. These CDs showed remarkable biocidal activity against Gram-negative bacteria by disturbing the bacterial enzymatic secondary structures and activities of cell wall-related enzymes, whereas they were less effective against Gram-positive bacteria.

### Applications in controlling phytopathogens and plant growth promotion

CDs with high hydrophilicity and high cell permeability are frequently applied in various biological applications. The antimicrobial application of CDs from green synthesis is less explored. Recently, CDs prepared from pomegranate and watermelon peel were evaluated against *Fusarium oxysporum*, *Staphylococcus aureus*, *Pseudomonas aeruginosa*, *Bacillus subtilis*, and *Escherichia coli*. The pomegranate-based CDs showed antibacterial and antifungal potential [188]. In another relevant study of Wang et al. [189], N-CDs synthesized from the roots of *Moringa oleifera* showed inhibitory effects against *Corynespora cassiicola* and *Phytophtora nicotianae*. In recent years, antimicrobial potential of CDs prepared from green synthesis were reported by many other researchers [190–192]. This could reduce the dependency on toxic synthetic compounds to control the pathogens [79].

CDs have been successfully employed in agriculture to enhance plant growth and produce of the crop. It was revealed that CDs being structurally similar to plant-hormones increased the activity of RuBisCO and liberated CO_2_. The CDs served as a fuel for anabolic photosynthetic pathways in rice plants [193]. It was revealed that the application of carbon-based nanomaterials enhanced the cell culture growth in tobacco and increase seed germination and plant growth in tomato seedlings [194].

In a similar study, the effect of water-soluble CDs on wheat plants was tested both under dark and light conditions. The application of these CDs supports the uptake of nutrients and water and significantly enhances the root and shoot growth [195]. Growth promotion behavior of CDs was also studied well in Rome lettuce [196], *Morus alba* [197], soybeans (*Glycine max*), and tomatoes (*Solanum lycopersicum*). The CDs have a wide application window and low toxicity effects and, hence, can be employed to get better crop production. The role of nanomaterials in microbial bioimaging, detection, sensing, viability testing, microbial control, and plant growth promotion is presented in Table 7.

**Table 7:** Green sources, methods of preparation, and applications of CDs synthesized from different sources.

S. No	Source	Method of preparation	Application	Ref

1	*Escherichia coli*	hydrothermal synthesis process	imaging and detection of p-nitrophenol	[198]
2	tulsi leaves	one step hydrothermal process	Cr sensor, bioimaging and as a patterning agent	[152]
3	apple juice	one step hydrothermal process	bioimaging of mycobacterium and fungal cells	[178]
4	*Cannabis sativa*	one-step pyrolysis method	antibacterial activity	[199]
5	wheat straw	hydrothermal treatment	labeling, imaging, and sensing	[200]
6	*Lycii fructus*	one step hydrothermal process	detection of phoxim in environmental and fruit samples	[201]
7	cow milk	hydrothermal treatment	antibacterial activity	[28]
8	coriander leaves	one-step hydrothermal treatment	antioxidants, biosensors and bioimaging	[26]
9	tamarind and calf thymus DNA	–	antimicrobial potential, cytotoxicity, and DNA binding	[202]
10	rapeseed pollen	one-step hydrothermal treatment	bioimaging and plant growth promotion	[196]
11	natural honey	hydrothermal treatment	photonic and antibacterial activity	[101]
12	*Chionanthus retusus*	hydrothermal-carbonization method	metal ion sensing and biological activities	[94]
13	mushroom	hydrothermal-carbonization method	metal ion detection, antibacterial and anticancer activities	[157]
14	nitrogenase extracted from Azotobacter	–	improvements in nitrogen fixation ability of *Azotobacter chroococcum*	[203]
15	*Actinidia deliciosa*	one-step hydrothermal treatment	catalytic activity and cytotoxicity applications	[204]
16	chicken egg whites	one-step heating reaction	sensing of bacteria and curcumin	[169]

### Critical appraisal, future perspectives, and challenges

(1) Inconsistencies were observed in different synthesis procedures for CDs regarding size distribution and PL properties. Furthermore, low yield and prolonged purification restraint the applications. Effective synthesis strategies need to be devised to elevate the quantum yields with small size distribution. A further investigation into the non-uniformity of physical properties of CDs is required to unveil their true nature and explore their potentiality.

(2) The synthesis and purification methods are time-consuming, and the CDs exhibit rather high detection limits and low sensitivity, which need to be addressed. Some of the functional groups on the surface of CDs interfere with their sensitivity towards various metal ions and, hence, may show response towards more than one metal ion, rendering them less sensitive towards a specific metal ion.

(3) A complete understanding of the PL phenomena (multiphoton emissions) calls for attention, which would pave way for multifaceted applications. The PL quenching mechanisms are not clear and have been assigned mostly based on assumptions and hypotheses due to incomplete information. An approach based on theoretical calculations coupled with experimental results could help in getting a clearer picture of the actual phenomena.

(4) Efforts are required to tune the PL emissions to higher wavelengths than green and blue. So far, mostly blue and green emissions in green source-derived CDs have been reported.

(5) To promote stability and in attempt to achieve emission at longer wavelengths, doping with various reagents is performed. This increases cytotoxicity and compromises the biocompatibility, rendering the CDs less suitable for biological applications. Alternative ways to achieve the aforementioned objectives need to be investigated.

(6) Green synthesis of CDs has opened many ways to explore their application in a variety of fields. So far, these green source CDs, due to their cost-effectiveness, biocompatibility, and low toxicity, are extensively employed in the detection of metal ions, biosensing, bioimaging, and as antibacterial and antifungal agents in biomedical sciences. In other major sectors such as agriculture, the potential role of green CDs has not yet been explored and only a few studies are available. Crops are attacked by various notorious phytopathogens including viruses, bacteria, fungi, and nematodes. These phytopathogens could result in devastating crop yield and economic losses. The extensive application of synthetic pesticides is a major threat to humans, animals, and the environment. Extensive testing and utilization of green source CDs in phytopathogen detection, bioimaging, and as biocontrol agents could provide an environmentally friendly alternative to synthetic agrochemicals.

(7) Very limited information is available on the interaction mechanisms and impact of CDs on the soilborne microbes beneficial for plants. A few reports have highlighted the positive impact of CDs on the soil microbiome. However, exposure to CDs may also harm the beneficial microbes and their nutrient recycling activities. However, the mechanism of toxicity is not well elaborated. It is important to explore the fate and effects of CDs under the relevant environmental conditions to incorporate them into agricultural systems.

(8) Easy, efficient, inexpensive, and sustainable strategies need to be explored with main focus on biocompatibility and low cytotoxicity for biomedical diagnostics and therapeutic applications.

(9) Structural characteristics of the CDs need to be unfolded to fully understand and exploit their applications in bioimaging and other biological applications.

(10) CDs derived from green sources mostly have low cytotoxicity. Therefore, their potential applications in areas such as drug delivery, radiation prevention, and early detection of illnesses need to be extensively investigated, as there is little to no work available. Similarly, no work is reported on the use of CDs in photodynamic and photothermal therapy.

## Conclusion

Since the discovery of carbon dots in 2004, there has been extensive research on the synthesis, modifications, properties, and applications, because of their distinctive and tunable physicochemical, optical, and electronic properties. We have attempted to review the recent advances in the field focusing on the synthesis of CDs from various green sources, their applications as sensing probes, in addition to their biological applications. Carbon dots prepared through green synthesis processes display multifaceted characters and broad-spectrum applications. Due to inexpensiveness and the low toxicity, CDs are widely employed in sensing and various biological applications, such as microbial bioimaging, detection, and viability studies as well as in pathogen inhibition and plant growth promotion. Their features make CDs ideal to be used in biological and other disciplines such as bioimaging, cancer therapy, gene and drug delivery, sensors and biosensors, catalysts, and energy applications. Regarding the green synthesis of CDs, there is a dire need to improve and standardize the synthesis mechanisms to obtain a high yield of CDs. Also mechanisms of quenching need to be investigated more. So far, applications of CDs in phytopathology are very limited. Wide experimental testing and research is needed to explore their natural potential in controlling notorious plant pathogens and plant growth promotion. Moreover, in medical science, there is a broad window to improve their efficiency and utilization in the early detection of diseases and in maintaining public health.

## References

[1] Xu X, Ray R, Gu Y, Ploehn H J, Gearheart L, Raker K, Scrivens W A (2004). J Am Chem Soc.

[2] Sun Y-P, Zhou B, Lin Y, Wang W, Fernando K A S, Pathak P, Meziani M J, Harruff B A, Wang X, Wang H (2006). J Am Chem Soc.

[3] Iravani S, Varma R S (2020). Environ Chem Lett.

[4] Qu Y, Yu L, Zhu B, Chai F, Su Z (2020). New J Chem.

[5] Sharma V, Singh S K, Mobin S M (2019). Nanoscale Adv.

[6] Jaiswal A, Ghosh S S, Chattopadhyay A (2012). Chem Commun.

[7] Wang W, Li Y, Cheng L, Cao Z, Liu W (2014). J Mater Chem B.

[8] Vedamalai M, Periasamy A P, Wang C-W, Tseng Y-T, Ho L-C, Shih C-C, Chang H-T (2014). Nanoscale.

[9] Fang L, Zhang L, Chen Z, Zhu C, Liu J, Zheng J (2017). Mater Lett.

[10] Schneider J, Reckmeier C J, Xiong Y, von Seckendorff M, Susha A S, Kasák P, Rogach A L (2017). J Phys Chem C.

[11] Liu F, Zhang W, Chen W, Wang J, Yang Q, Zhu W, Wang J (2017). Chem Eng J.

[12] Shinde D B, Pillai V K (2012). Chem – Eur J.

[13] Joseph J, Anappara A A (2017). ChemPhysChem.

[14] Das P, Ganguly S, Bose M, Ray D, Ghosh S, Mondal S, Aswal V K, Das A K, Banerjee S, Das N C (2019). New J Chem.

[15] Wang N, Wang Y, Guo T, Yang T, Chen M, Wang J (2016). Biosens Bioelectron.

[16] Lu M, Duan Y, Song Y, Tan J, Zhou L (2018). J Mol Liq.

[17] Li Z, Zhang Y, Niu Q, Mou M, Wu Y, Liu X, Yan Z, Liao S (2017). J Lumin.

[18] Sharma N, Yun K (2020). Dyes Pigm.

[19] Arumugam S S, Xuing J, Viswadevarayalu A, Rong Y, Sabarinathan D, Ali S, Agyekum A A, Li H, Chen Q (2020). J Photochem Photobiol, A.

[20] Singh A, Eftekhari E, Scott J, Kaur J, Yambem S, Leusch F, Wellings R, Gould T, Ostrikov K, Sonar P (2020). Sustainable Mater Technol.

[21] Jayanthi M, Megarajan S, Subramaniyan S B, Kamlekar R K, Veerappan A (2019). J Mol Liq.

[22] Wan Y, Wang M, Zhang K, Fu Q, Gao M, Wang L, Xia Z, Gao D (2019). Microchem J.

[23] Pourreza N, Ghomi M (2019). Mater Sci Eng, C.

[24] Sun Y-P, Wang X, Lu F, Cao L, Meziani M J, Luo P G, Gu L, Veca L M (2008). J Phys Chem C.

[25] Shi J, Ni G, Tu J, Jin X, Peng J (2017). J Nanopart Res.

[26] Sachdev A, Gopinath P (2015). Analyst.

[27] Gu J, Zhang X, Pang A, Yang J (2016). Nanotechnology.

[28] Han S, Zhang H, Xie Y, Liu L, Shan C, Li X, Liu W, Tang Y (2015). Appl Surf Sci.

[29] Sun X, He J, Yang S, Zheng M, Wang Y, Ma S, Zheng H (2017). J Photochem Photobiol, B.

[30] Başoğlu A, Ocak Ü, Gümrükçüoğlu A (2020). J Fluoresc.

[31] Aji M P, Susanto, Wiguna P A, Sulhadi (2017). J Theor Appl Phys.

[32] De B, Karak N (2017). J Mater Chem A.

[33] Wang R, Lu K-Q, Tang Z-R, Xu Y-J (2017). J Mater Chem A.

[34] Yuan F, Li S, Fan Z, Meng X, Fan L, Yang S (2016). Nano Today.

[35] Rani U A, Ng L Y, Ng C Y, Mahmoudi E (2020). Adv Colloid Interface Sci.

[36] Shi X, Wei W, Fu Z, Gao W, Zhang C, Zhao Q, Deng F, Lu X (2019). Talanta.

[37] Pirsaheb M, Mohammadi S, Salimi A (2019). TrAC, Trends Anal Chem.

[38] Zuo P, Lu X, Sun Z, Guo Y, He H (2016). Microchim Acta.

[39] Zheng X T, Ananthanarayanan A, Luo K Q, Chen P (2015). Small.

[40] Zhao A, Chen Z, Zhao C, Gao N, Ren J, Qu X (2015). Carbon.

[41] Liu M L, Chen B B, Li C M, Huang C Z (2019). Green Chem.

[42] Xu Q, Kuang T, Liu Y, Cai L, Peng X, Sreenivasan Sreeprasad T, Zhao P, Yu Z, Li N (2016). J Mater Chem B.

[43] Sharma V, Tiwari P, Mobin S M (2017). J Mater Chem B.

[44] Tejwan N, Saha S K, Das J (2020). Adv Colloid Interface Sci.

[45] Lin X, Xiong M, Zhang J, He C, Ma X, Zhang H, Kuang Y, Yang M, Huang Q (2021). Microchem J.

[46] Meng W, Bai X, Wang B, Liu Z, Lu S, Yang B (2019). Energy Environ Mater.

[47] Bag P, Maurya R K, Dadwal A, Sarkar M, Chawla P A, Narang R K, Kumar B (2021). ChemistrySelect.

[48] Bhamore J R, Jha S, Park T J, Kailasa S K (2019). J Photochem Photobiol, B.

[49] Ma H, Sun C, Xue G, Wu G, Zhang X, Han X, Qi X, Lv X, Sun H, Zhang J (2019). Spectrochim Acta, Part A.

[50] Liu M L, Yang L, Li R S, Chen B B, Liu H, Huang C Z (2017). Green Chem.

[51] Chauhan P, Dogra S, Chaudhary S, Kumar R (2020). Mater Today Chem.

[52] Komalavalli L, Amutha P, Monisha S (2020). Mater Today: Proc.

[53] Sun D, Liu T, Wang C, Yang L, Yang S, Zhuo K (2020). Spectrochim Acta, Part A.

[54] Zulfajri M, Abdelhamid H N, Sudewi S, Dayalan S, Rasool A, Habib A, Huang G G (2020). Biosensors.

[55] Liu S, Tian J, Wang L, Zhang Y, Qin X, Luo Y, Asiri A M, Al-Youbi A O, Sun X (2012). Adv Mater (Weinheim, Ger).

[56] Bhamore J R, Jha S, Singhal R K, Park T J, Kailasa S K (2018). J Mol Liq.

[57] Wei X, Li L, Liu J, Yu L, Li H, Cheng F, Yi X, He J, Li B (2019). ACS Appl Mater Interfaces.

[58] Wang C, Shi H, Yang M, Yan Y, Liu E, Ji Z, Fan J (2020). Mater Res Bull.

[59] Murugan N, Sundramoorthy A K (2018). New J Chem.

[60] Hoan B T, Tam P D, Pham V-H (2019). J Nanotechnol.

[61] Sahoo N K, Jana G C, Aktara M N, Das S, Nayim S, Patra A, Bhattacharjee P, Bhadra K, Hossain M (2020). Mater Sci Eng, C.

[62] Vasimalai N, Vilas-Boas V, Gallo J, de Fátima Cerqueira M, Menéndez-Miranda M, Costa-Fernández J M, Diéguez L, Espiña B, Fernández-Argüelles M T (2018). Beilstein J Nanotechnol.

[63] Vandarkuzhali S A A, Jeyalakshmi V, Sivaraman G, Singaravadivel S, Krishnamurthy K R, Viswanathan B (2017). Sens Actuators, B.

[64] Eskalen H, Uruş S, Cömertpay S, Kurt A H, Özgan Ş (2020). Ind Crops Prod.

[65] Li L-S, Jiao X-Y, Zhang Y, Cheng C, Huang K, Xu L (2018). Sens Actuators, B.

[66] Zhai H, Zheng B, Yang F, Wang M, Xiao D (2018). Anal Methods.

[67] Devi P, Thakur A, Bhardwaj S K, Saini S, Rajput P, Kumar P (2018). J Mater Sci: Mater Electron.

[68] Kavitha T, Kumar S (2018). Sci Rep.

[69] Devi P, Kaur G, Thakur A, Kaur N, Grewal A, Kumar P (2017). Talanta.

[70] Athinarayanan J, Periasamy V S, Alatiah K A, Alshatwi A A (2020). Sustainable Chem Pharm.

[71] Ramanarayanan R, Swaminathan S (2020). Mater Today: Proc.

[72] Pandey S C, Kumar A, Sahu S K (2020). J Photochem Photobiol, A.

[73] Chaudhary P, Maurya D K, Yadav S, Pandey A, Tripathi R K, Yadav B C (2021). Sens Actuators, B.

[74] Qiu Y, Gao D, Yin H, Zhang K, Zeng J, Wang L, Xia L, Zhou K, Xia Z, Fu Q (2020). Sens Actuators, B.

[75] Shekarbeygi Z, Farhadian N, Khani S, Moradi S, Shahlaei M (2020). Microchem J.

[76] Al-Hashimi B, Omer K M, Rahman H S (2020). Arabian J Chem.

[77] Ding S, Gao Y, Ni B, Yang X (2021). Inorg Chem Commun.

[78] Li Z, Wang Q, Zhou Z, Zhao S, Zhong S, Xu L, Gao Y, Cui X (2021). Microchem J.

[79] Atchudan R, Jebakumar Immanuel Edison T N, Shanmugam M, Perumal S, Somanathan T, Lee Y R (2021). Phys E (Amsterdam, Neth).

[80] Sharma N, Sharma I, Bera M K (2022). J Fluoresc.

[81] Atchudan R, Edison T N J I, Perumal S, Vinodh R, Sundramoorthy A K, Babu R S, Lee Y R (2022). Colloids Surf, A.

[82] Zhang Q, Tian F, Zhou Q, Zhang C, Tang S, Jiang L, Du S (2022). Inorg Chem Commun.

[83] D P, Singh L, Thakur A, Kumar P (2019). Sens Actuators, B.

[84] Ansi V A, Renuka N K (2018). Sens Actuators, B.

[85] Gupta D A, Desai M L, Malek N I, Kailasa S K (2020). J Mol Struct.

[86] Long R, Guo Y, Xie L, Shi S, Xu J, Tong C, Lin Q, Li T (2020). Food Chem.

[87] Hu G, Ge L, Li Y, Mukhtar M, Shen B, Yang D, Li J (2020). J Colloid Interface Sci.

[88] Mary Alex A, Kiran M D, Hari G, Krishnan A, Jayan J S, Saritha A (2020). Mater Today: Proc.

[89] Xie Y, Cheng D, Liu X, Han A (2019). Sensors.

[90] Bandi R, Dadigala R, Gangapuram B R, Guttena V (2018). J Photochem Photobiol, B.

[91] Varisco M, Zufferey D, Ruggi A, Zhang Y, Erni R, Mamula O (2017). R Soc Open Sci.

[92] Pal T, Mohiyuddin S, Packirisamy G (2018). ACS Omega.

[93] Arul V, Edison T N J I, Lee Y R, Sethuraman M G (2017). J Photochem Photobiol, B.

[94] Atchudan R, Edison T N J I, Chakradhar D, Perumal S, Shim J-J, Lee Y R (2017). Sens Actuators, B.

[95] Arul V, Sethuraman M G (2019). ACS Omega.

[96] Atchudan R, Edison T N J I, Aseer K R, Perumal S, Karthik N, Lee Y R (2018). Biosens Bioelectron.

[97] Abdullah Issa M, Abidin Z Z, Sobri S, Abdul-Rashid S, Mahdi M A, Ibrahim N A, Pudza M Y (2020). Chin J Chem Eng.

[98] Issa M A, Abidin Z Z, Sobri S, Rashid S, Mahdi M A, Ibrahim N A, Pudza M Y (2019). Nanomaterials.

[99] Putro P A, Roza L, Isnaeni I (2019). Indonesien J Sci Educ.

[100] Tu Y, Wang S, Yuan X, Wei Y, Qin K, Zhang Q, Chen X, Ji X (2020). Dyes Pigm.

[101] Surendran P, Lakshmanan A, Priya S S, Balakrishnan K, Rameshkumar P, Kannan K, Geetha P, Hegde T A, Vinitha G (2020). Nano-Struct Nano-Objects.

[102] Xu X, Cai L, Hu G, Mo L, Zheng Y, Hu C, Lei B, Zhang X, Liu Y, Zhuang J (2020). J Lumin.

[103] Atchudan R, Edison T N J I, Aseer K R, Perumal S, Lee Y R (2018). Colloids Surf, B.

[104] Huang Q, Li Q, Chen Y, Tong L, Lin X, Zhu J, Tong Q (2018). Sens Actuators, B.

[105] Godavarthi S, Mohan Kumar K, Vázquez Vélez E, Hernandez-Eligio A, Mahendhiran M, Hernandez-Como N, Aleman M, Martinez Gomez L (2017). J Photochem Photobiol, B.

[106] Yadav P K, Singh V K, Chandra S, Bano D, Kumar V, Talat M, Hasan S H (2019). ACS Biomater Sci Eng.

[107] Bi X, Hou X, Zhang X, Liu W, Ren G, Xu S, Wang H, Wei W (2021). ChemistrySelect.

[108] Gu D, Zhang P, Zhang L, Liu H, Pu Z, Shang S (2018). Opt Mater (Amsterdam, Neth).

[109] Singh A K, Singh V K, Singh M, Singh P, Khadim S R, Singh U, Koch B, Hasan S H, Asthana R K (2019). J Photochem Photobiol, A.

[110] Wei Z, Wang B, Liu Y, Liu Z, Zhang H, Zhang S, Chang J, Lu S (2019). New J Chem.

[111] Sabet M, Mahdavi K (2019). Appl Surf Sci.

[112] Li L, Wang X, Fu Z, Cui F (2017). Mater Lett.

[113] Lin R, Cheng S, Tan M (2022). Food Funct.

[114] Dehvari K, Liu K Y, Tseng P-J, Gedda G, Girma W M, Chang J-Y (2019). J Taiwan Inst Chem Eng.

[115] Zhao C, Jiao Y, Hu F, Yang Y (2018). Spectrochim Acta, Part A.

[116] Zhang X-D, Li J, Niu J-N, Bao X-P, Zhao H-D, Tan M (2019). Methods.

[117] Chakraborty D, Sarkar S, Das P K (2018). ACS Sustainable Chem Eng.

[118] Zhou Y, Liu Y, Li Y, He Z, Xu Q, Chen Y, Street J, Guo H, Nelles M (2018). RSC Adv.

[119] Jia J, Lin B, Gao Y, Jiao Y, Li L, Dong C, Shuang S (2019). Spectrochim Acta, Part A.

[120] Kaur N, Sharma V, Tiwari P, Saini A K, Mobin S M (2019). Sens Actuators, B.

[121] Khan Z M S H, Rahman R S, Shumaila, Islam S, Zulfequar M (2019). Opt Mater (Amsterdam, Neth).

[122] Yu C, Xuan T, Yan D, Lou S, Hou X, Chen Y, Wang J, Li H (2017). Sens Actuators, B.

[123] Soni H, Pamidimukkala P S (2018). Mater Res Bull.

[124] Chen W, Li D, Tian L, Xiang W, Wang T, Hu W, Hu Y, Chen S, Chen J, Dai Z (2018). Green Chem.

[125] Wen X, Shi L, Wen G, Li Y, Dong C, Yang J, Shuang S (2015). Sens Actuators, B.

[126] Zhang Z, Sun W, Wu P (2015). ACS Sustainable Chem Eng.

[127] Chen W, Hu C, Yang Y, Cui J, Liu Y (2016). Materials.

[128] Ma X, Dong Y, Sun H, Chen N (2017). Mater Today Chem.

[129] Pires N R, Santos C M W, Sousa R R, De Paula R C M, Cunha P L R, Feitosa J P A (2015). J Braz Chem Soc.

[130] Wu X, Ma C, Liu J, Liu Y, Luo S, Xu M, Wu P, Li W, Liu S (2019). ACS Sustainable Chem Eng.

[131] Wang L, Zhu S-J, Wang H-Y, Qu S-N, Zhang Y-L, Zhang J-H, Chen Q-D, Xu H-L, Han W, Yang B (2014). ACS Nano.

[132] Song Y, Zhu S, Zhang S, Fu Y, Wang L, Zhao X, Yang B (2015). J Mater Chem C.

[133] Essner J B, Kist J A, Polo-Parada L, Baker G A (2018). Chem Mater.

[134] Righetto M, Privitera A, Fortunati I, Mosconi D, Zerbetto M, Curri M L, Corricelli M, Moretto A, Agnoli S, Franco L (2017). J Phys Chem Lett.

[135] Roy P, Chen P-C, Periasamy A P, Chen Y-N, Chang H-T (2015). Mater Today.

[136] Li X, Lau S P, Tang L, Ji R, Yang P (2014). Nanoscale.

[137] Mahmood A (2015). Handbook of Nanoparticles.

[138] Venkatesan G, Rajagopalan V, Chakravarthula S N (2019). J Environ Chem Eng.

[139] Thakur A, Devi P, Saini S, Jain R, Sinha R K, Kumar P (2019). ACS Sustainable Chem Eng.

[140] Raja D, Sundaramurthy D (2021). Mater Today: Proc.

[141] Picard M, Thakur S, Misra M, Mohanty A K (2019). RSC Adv.

[142] Liu G, Jia H, Li N, Li X, Yu Z, Wang J, Song Y (2019). Microchem J.

[143] Tadesse A, Hagos M, RamaDevi D, Basavaiah K, Belachew N (2020). ACS Omega.

[144] Kumari A, Kumar A, Sahu S K, Kumar S (2018). Sens Actuators, B.

[145] Praneerad J, Thongsai N, Supchocksoonthorn P, Kladsomboon S, Paoprasert P (2019). Spectrochim Acta, Part A.

[146] Murugan N, Prakash M, Jayakumar M, Sundaramurthy A, Sundramoorthy A K (2019). Appl Surf Sci.

[147] Zainal Abidin N H, Wongso V, Hui K C, Cho K, Sambudi N S, Ang W L, Saad B (2020). J Water Process Eng.

[148] Athika M, Prasath A, Duraisamy E, Sankar Devi V, Selva Sharma A, Elumalai P (2019). Mater Lett.

[149] V R, Misra S, Santra M K, Ottoor D (2019). J Photochem Photobiol, A.

[150] Das P, Maruthapandi M, Saravanan A, Natan M, Jacobi G, Banin E, Gedanken A (2020). ACS Appl Nano Mater.

[151] Luo B, Yang H, Zhou B, Ahmed S M, Zhang Y, Liu H, Liu X, He Y, Xia S (2020). ACS Omega.

[152] Bhatt S, Bhatt M, Kumar A, Vyas G, Gajaria T, Paul P (2018). Colloids Surf, B.

[153] Zhao C, Li X, Cheng C, Yang Y (2019). Microchem J.

[154] Ramanan V, Siddaiah B, Raji K, Ramamurthy P (2018). ACS Sustainable Chem Eng.

[155] Ramezani Z, Qorbanpour M, Rahbar N (2018). Colloids Surf, A.

[156] Radhakrishnan K, Panneerselvam P (2018). RSC Adv.

[157] Boobalan T, Sethupathi M, Sengottuvelan N, Kumar P, Balaji P, Gulyás B, Padmanabhan P, Selvan S T, Arun A (2020). ACS Appl Nano Mater.

[158] Ji X, Yuan X, Nian H, Song P, Xiang Y, Wei Y, Wang S, Qin K, Zhang Q, Tu Y (2020). Dyes Pigm.

[159] Shariati-Rad M, Mohseninasab T, Parno F (2018). RSC Adv.

[160] Man Y, Li Z, Kong W-L, Li W, Dong W, Wang Y, Xie F, Zhao D, Qu Q, Zou W-S (2020). Microchem J.

[161] Arumugham T, Alagumuthu M, Amimodu R G, Munusamy S, Iyer S K (2020). Sustainable Mater Technol.

[162] Chaudhary N, Gupta P K, Eremin S, Solanki P R (2020). J Environ Chem Eng.

[163] Kumar A, Chowdhuri A R, Laha D, Mahto T K, Karmakar P, Sahu S K (2017). Sens Actuators, B.

[164] Lin F, Bao Y-W, Wu F-G (2019). C–Open Access Carbon Res J.

[165] Dinç S, Kara M (2018). J Apither Nat.

[166] Peralta-Videa J, Sreenivasan S T, Narayan M (2020). Processes.

[167] Elmer W, White J C (2018). Annu Rev Phytopathol.

[168] Anand A, Manavalan G, Mandal R P, Chang H-T, Chiou Y-R, Huang C-C (2020). Curr Pharm Des.

[169] Baig M M F, Chen Y-C (2017). J Colloid Interface Sci.

[170] Kasibabu B S B, D'souza S L, Jha S, Singhal R K, Basu H, Kailasa S K (2015). Anal Methods.

[171] Kasibabu B S B, D’souza S L, Jha S, Kailasa S K (2015). J Fluoresc.

[172] Nandi S, Ritenberg M, Jelinek R (2015). Analyst.

[173] Zhou H, Yang D, Ivleva N P, Mircescu N E, Schubert S, Niessner R, Wieser A, Haisch C (2015). Anal Chem (Washington, DC, U S).

[174] Gu Y, Hu Y, Zhao X, Chen X, Wang P, Zheng Z (2018). Opt Express.

[175] Hua X-W, Bao Y-W, Wang H-Y, Chen Z, Wu F-G (2017). Nanoscale.

[176] Song Y, Li H, Lu F, Wang H, Zhang M, Yang J, Huang J, Huang H, Liu Y, Kang Z (2017). J Mater Chem B.

[177] Zhong D, Zhuo Y, Feng Y, Yang X (2015). Biosens Bioelectron.

[178] Mehta V N, Jha S, Basu H, Singhal R K, Kailasa S K (2015). Sens Actuators, B.

[179] Li H, Huang J, Song Y, Zhang M, Wang H, Lu F, Huang H, Liu Y, Dai X, Gu Z (2018). ACS Appl Mater Interfaces.

[180] Yang J, Gao G, Zhang X, Ma Y-H, Chen X, Wu F-G (2019). Carbon.

[181] Travlou N A, Algarra M, Alcoholado C, Cifuentes-Rueda M, Labella A M, Lázaro-Martínez J M, Rodríguez-Castellón E, Bandosz T J (2018). ACS Appl Bio Mater.

[182] Ting D, Dong N, Fang L, Lu J, Bi J, Xiao S, Han H (2018). ACS Appl Nano Mater.

[183] Shivaji K, Mani S, Ponmurugan P, De Castro C S, Lloyd Davies M, Balasubramanian M G, Pitchaimuthu S (2018). ACS Appl Nano Mater.

[184] Shahshahanipour M, Rezaei B, Ensafi A A, Etemadifar Z (2019). Mater Sci Eng, C.

[185] Yallappa S, Deepthi D R, Yashaswini S, Hamsanandini R, Chandraprasad M, Ashok Kumar S, Hegde G (2017). Nano-Struct Nano-Objects.

[186] Pandiyan S, Arumugam L, Srirengan S P, Pitchan R, Sevugan P, Kannan K, Pitchan G, Hegde T A, Gandhirajan V (2020). ACS Omega.

[187] Wang H, Zhang M, Ma Y, Wang B, Shao M, Huang H, Liu Y, Kang Z (2020). J Mater Chem B.

[188] Muktha H, Sharath R, Kottam N, Smrithi S P, Samrat K, Ankitha P (2020). Bionanosci.

[189] Wang Z, Liu Q, Leng J, Liu H, Zhang Y, Wang C, An W, Bao C, Lei H (2021). J Saudi Chem Soc.

[190] Meela M M, Mdee L K, Masoko P, Eloff J N (2019). S Afr J Bot.

[191] Abdel-Rahman F A, Rashid I A S, Shoala T (2020). J Plant Prot Res.

[192] Khayal A, Dawane V, Amin M A, Tirth V, Yadav V K, Algahtani A, Khan S H, Islam S, Yadav K K, Jeon B-H (2021). Polymers (Basel, Switz).

[193] Li H, Huang J, Lu F, Liu Y, Song Y, Sun Y, Zhong J, Huang H, Wang Y, Li S (2018). ACS Appl Bio Mater.

[194] Lahiani M H, Dervishi E, Ivanov I, Chen J, Khodakovskaya M (2016). Nanotechnology.

[195] Tripathi S, Sarkar S (2015). Appl Nanosci.

[196] Zheng Y, Xie G, Zhang X, Chen Z, Cai Y, Yu W, Liu H, Shan J, Li R, Liu Y (2017). ACS Omega.

[197] Waghmare R D, Gore A H, Anbhule P V, Sohn D, Kolekar G B (2020). Data Brief.

[198] Qin K, Zhang D, Ding Y, Zheng X, Xiang Y, Hua J, Zhang Q, Ji X, Li B, Wei Y (2020). Analyst.

[199] Raina S, Thakur A, Sharma A, Pooja D, Minhas A P (2020). Mater Lett.

[200] Yuan M, Zhong R, Gao H, Li W, Yun X, Liu J, Zhao X, Zhao G, Zhang F (2015). Appl Surf Sci.

[201] Zheng M, Wang C, Wang Y, Wei W, Ma S, Sun X, He J (2018). Talanta.

[202] Jhonsi M A, Ananth D A, Nambirajan G, Sivasudha T, Yamini R, Bera S, Kathiravan A (2018). Spectrochim Acta, Part A.

[203] Wang H, Li H, Zhang M, Song Y, Huang J, Huang H, Shao M, Liu Y, Kang Z (2018). ACS Appl Mater Interfaces.

[204] Arul V, Sethuraman M G (2018). Opt Mater (Amsterdam, Neth).

